# Prd1 associates with the clathrin adaptor α-Adaptin and the kinesin-3 Imac/Unc-104 to govern dendrite pruning in *Drosophila*

**DOI:** 10.1371/journal.pbio.2004506

**Published:** 2018-08-24

**Authors:** Wenhui Zong, Yan Wang, Quan Tang, Heng Zhang, Fengwei Yu

**Affiliations:** 1 Temasek Life Sciences Laboratory and Department of Biological Sciences, 1 Research Link, National University of Singapore, Singapore; 2 NUS Graduate School for Integrative Sciences and Engineering, Centre for Life Sciences, Singapore; 3 Neuroscience and Behavioral Disorder Program, Duke-NUS Graduate Medical School Singapore, Singapore; Oregon Health and Science University, United States of America

## Abstract

Refinement of the nervous system depends on selective removal of excessive axons/dendrites, a process known as pruning. *Drosophila* ddaC sensory neurons prune their larval dendrites via endo-lysosomal degradation of the L1-type cell adhesion molecule (L1-CAM), Neuroglian (Nrg). Here, we have identified a novel gene, *pruning defect 1* (*prd1*), which governs dendrite pruning of ddaC neurons. We show that Prd1 colocalizes with the clathrin adaptor protein α-Adaptin (α-Ada) and the kinesin-3 immaculate connections (Imac)/Uncoordinated-104 (Unc-104) in dendrites. Moreover, Prd1 physically associates with α-Ada and Imac, which are both critical for dendrite pruning. Prd1, α-Ada, and Imac promote dendrite pruning via the regulation of endo-lysosomal degradation of Nrg. Importantly, genetic interactions among *prd1*, *α-adaptin*, and *imac* indicate that they act in the same pathway to promote dendrite pruning. Our findings indicate that Prd1, α-Ada, and Imac act together to regulate discrete distribution of α-Ada/clathrin puncta, facilitate endo-lysosomal degradation, and thereby promote dendrite pruning in sensory neurons.

## Author summary

During the maturation of the nervous system, some neurons can selectively eliminate their unnecessary connections, including dendrites and axons, to retain specific connections. In *Drosophila*, a class of sensory neurons lose all their larval dendrites during metamorphosis, when they transition from larvae to adults. We previously showed that these neurons prune their dendrites via lysosome-mediated degradation of a cell-adhesion protein, Neuroglian. In this paper, we identified a previously uncharacterized gene, *pruning defect 1* (*prd1*), which plays an important role in dendrite pruning. We show that Prd1 is localized and complexed with α-Adaptin and Imac, two other proteins that are also essential for dendrite pruning. Moreover, Prd1, α-Adaptin, and Imac act in a common pathway to promote dendrite pruning by down-regulating Neuroglian protein. Thus, our study highlights a mechanism whereby Prd1, α-Adaptin, and Imac act together to regulate distribution of α-Adaptin/clathrin puncta, facilitate lysosome-dependent protein degradation, and thereby promote dendrite pruning in *Drosophila* sensory neurons.

## Introduction

Neuronal remodeling is a pivotal step in the formation of mature nervous systems during animal development. Developing neurons often outgrow superfluous axonal or dendritic branches at early developmental stages. Selective elimination of the unneeded branches without the death of parent neurons, referred to as pruning, is crucial for the refinement of neuronal circuits at late stages [1–3]. Neuronal pruning is a naturally occurring process in mammals and insects. In the central and peripheral nervous systems of mammals, many neurons often prune their unwanted or inappropriate neurites in order to establish proper and functional neuronal connections [4–6]. In insects, such as *Drosophila*, the nervous system is drastically remodeled during metamorphosis [7–9]. In the central nervous system, mushroom body (MB) γ neurons prune their larval axonal/dendritic branches and extend their adult-specific processes to be integrated into the adult brains prior to eclosion [10]. In the peripheral nervous system, dendritic arborization (da) neurons undergo either apoptosis or pruning during early metamorphosis. For example, in the dorsal cluster, class IV da neurons (ddaC) and class I da neurons (ddaD and ddaE) selectively eliminate their larval dendrites whereas their axons remain intact [11,12], while class III da neurons (ddaF) are apoptotic [12]. The dendrite-specific pruning event involves the formation of swellings and retracting bulbs [12], morphologically resembling the axon/dendrite degenerative process associated with brain injury and neurodegenerative diseases. Thus, understanding the mechanisms of developmental pruning would provide insight into neurodegeneration in pathological conditions.

**Fig 1:**
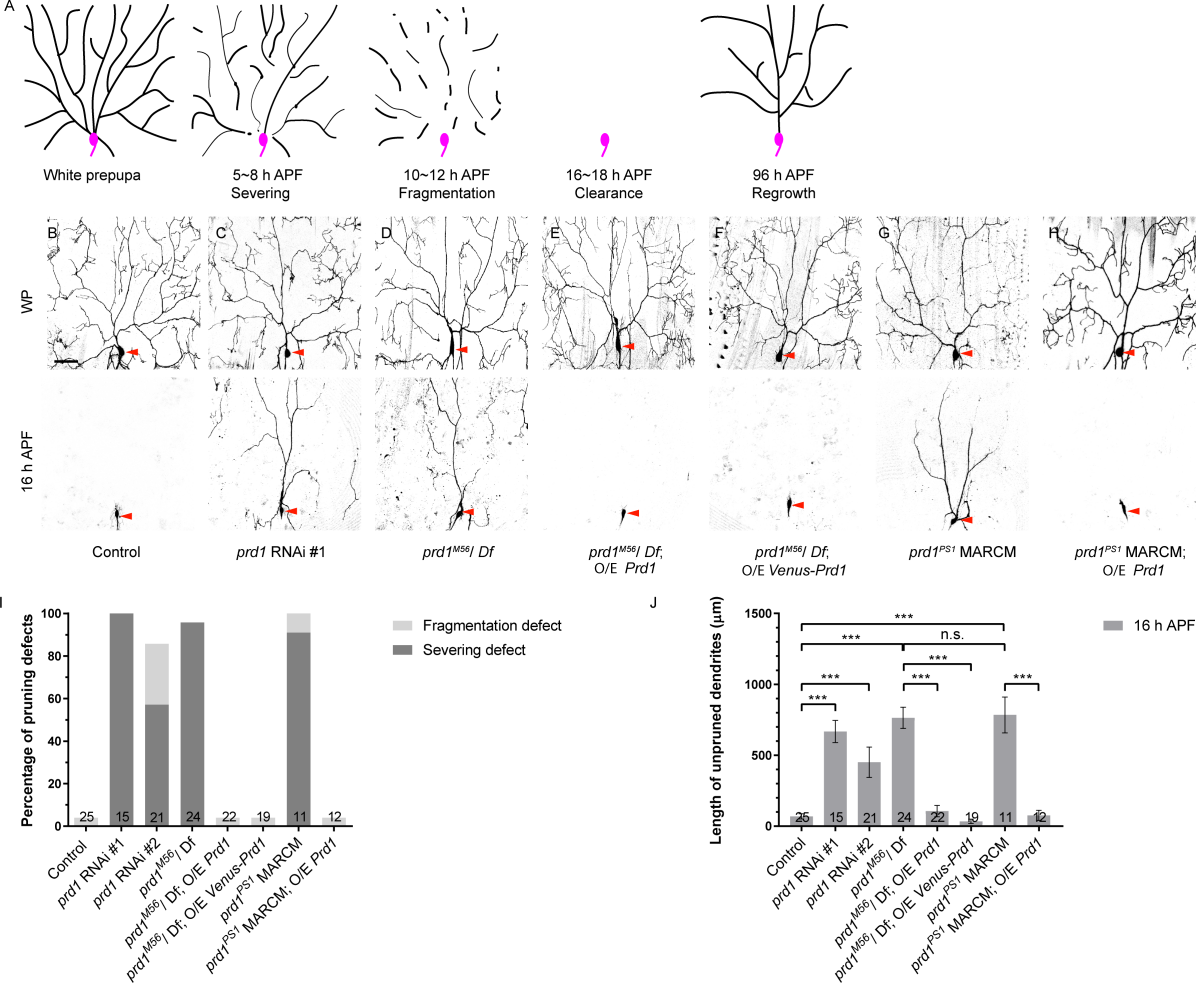
*prd1* is required for dendrite pruning in sensory neurons. (A) A schematic representation of dendrite pruning and regrowth in ddaC neurons during early metamorphosis. Soma and axon are depicted in pink while dendrites are in black. (B–H) Dendrites of control (B), *prd1* RNAi #1 (C), *prd1*^*M56*^*/Df(3R)Exel7310* (D), *prd1*^*M56*^ rescue using full-length Prd1 (E), or Venus-Prd1 (F), *prd1*^*PS1*^ MARCM (G) and *prd1*^*PS1*^ MARCM rescue using full-length Prd1 (H) ddaC neurons at WP and 16 h APF. Red arrowheads point to the ddaC somas. (I) Quantification of severing defect and fragmentation defect in control and mutant ddaC neurons at 16 h APF. (J) Quantification of total length of unpruned ddaC dendrites at 16 h APF. The number of samples (*n*) in each group is shown on the bars. Error bars represent SEM. Scale bar in (B) represents 50 μm. ***p < 0.001 as assessed by one-way ANOVA test. The individual numerical values for panels I and J can be found in S1 Data. The genotypes can be found in S1 Text. APF, after puparium formation; MARCM, mosaic analysis with a repressible cell marker; n.s., not significant; O/E, overexpression; *prd1*, *pruning defect 1*; RNAi, RNA interference; WP, white prepupal.

Dendrite pruning of *Drosophila* ddaC sensory neurons has emerged as an attractive paradigm to elucidate the molecular and cellular mechanisms of neuronal pruning. In response to a late larval pulse of the steroid-molting hormone 20-hydroxyecdysone (ecdysone), the dendrites of ddaC neurons are severed at their proximal region at 5–8 h after puparium formation (APF), and subsequently the detached dendrites are rapidly fragmented and undergo phagocytosis-mediated degradation by 16–18 h APF (Fig 1A) [11,12]. It has been well documented that ecdysone and its nuclear receptors are required to induce the expression of several major downstream targets to initiate dendrite pruning [13,14].

Clathrin-mediated endocytosis (CME) is a major entry route that regulates the surface expression of transmembrane proteins and turnover of lipid membrane in eukaryotic cells [15]. The process is mediated by the highly conserved heterotetrameric Adaptor protein-2 (AP-2) protein complex composed of α, β, μ, and σ subunits [16]. AP-2 is a CME-specific clathrin-associated adaptor that recruits clathrin to the plasma membrane-specific lipid phosphatidylinositol 4,5-bisphosphate (PtdIns(4,5)P_2_), leading to the formation of clathrin-coated pits. The invaginated pits are cleaved by the small GTPase dynamin to form clathrin-coated endocytic vesicles. Newly formed endocytic vesicles can fuse with early endosomes, a process mediated by the key GTPase Rabaptin-5 (Rab5) [17]. Among several downstream routes, early endosomes can mature into multivesicular bodies in an endosomal sorting complexes required for transport (ESCRT)-dependent manner and subsequently fuse with lysosomes to degrade their protein and membrane components, a process known as endo-lysosomal maturation and degradation pathway [18]. It has been reported that CME regulates axon growth/guidance, dendrite extension/branching, and synaptic vesicle trafficking in vertebrate and invertebrate neurons [19,20].

We and others have also reported that endocytosis as well as endo-lysosomal degradation pathway play critical roles in ddaC dendrite pruning and MB γ axon pruning in *Drosophila* [21–23]. Rab5 and ESCRT complexes, two key regulators of the endo-lysosomal degradation pathway, promote dendrite pruning in ddaC neurons by facilitating lysosomal degradation of the *Drosophila* L1-type cell adhesion molecule (L1-CAM) Neuroglian (Nrg) [23]. Nrg is drastically endocytosed and degraded in dendrites, axons, and soma of ddaC neurons prior to pruning [23]. A parallel study also showed that Rab5/dynamin-dependent endocytosis appears to predominantly occur at the proximal regions of dendrites, leading to dendritic thinning and compartmentalized Ca^2+^ transients in ddaC neurons [22]. In MB γ neurons, PI3K-cIII/dynamin-dependent endo-lysosomal degradation pathway down-regulates the Hedgehog receptor Patched to promote axon pruning [21]. These studies highlight a general requirement of endocytosis and endo-lysosomal degradation for regulating distinct modes of neuronal pruning. However, the regulatory mechanism that promotes endocytosis and endo-lysosomal degradation pathway during neuronal pruning remains poorly understood.

Here, we identified the critical role of a novel *Drosophila SKIP*-related gene, *pruning defect 1 (prd1)*, in regulating dendrite pruning of ddaC sensory neurons. Mammalian *Salmonella* induced filament A (SifA) and Kinesin-interacting protein (SKIP, also known as PLEKHM2) was originally identified as a target of the *Salmonella* effector protein SifA [24]. In uninfected mammalian cells, SKIP regulates the distribution of late endosomes and lysosomes [25,26]. However, the in vivo roles of SKIP and its related homologues during animal development are unknown. We show that Prd1 colocalizes with the endocytic components α-Adaptin (α-Ada) and clathrin in the dendrites of ddaC neurons. It forms a protein complex with α-Ada, the α subunit of the AP-2 complex (also known as AP-2α), but not with the endosomal GTPase Rab5. Similar to Prd1, α-Ada and other subunits of AP-2 complex are all required for dendrite pruning in sensory neurons. Moreover, we show that Prd1 colocalizes and associates with the kinesin-3 immaculate connections (Imac)/Uncoordinated-104 (Unc-104). Importantly, both *imac* mutants and dominant-negative constructs exhibited severe dendrite pruning defects in ddaC sensory neurons. Prd1, α-Ada, and Imac facilitate endo-lysosomal degradation of Nrg prior to dendrite pruning. Furthermore, genetic interactions among *prd1*, *α-ada*, and *imac* suggest that they participate in the same pathway to promote dendrite pruning. Thus, our data demonstrate that the Prd1/α-Ada/Imac pathway promotes dendrite pruning via regulating α-Ada/clathrin distribution and endo-lysosomal degradation of Nrg.

## Results

### *prd1* is required for dendrite pruning in sensory neurons

To identify novel players in dendrite pruning, we expressed a collection of RNA interference (RNAi) lines using a class IV da neuron driver *pickpocket-Gal4* (*ppk-Gal4*) to knock down gene function in ddaC neurons. Two independent RNAi lines, v108557 (#1) and v40070 (#2), were isolated with dendrite pruning defects. Both of RNAi lines target against a novel gene, *CG17360*, which we therefore named *pruning defect 1 (prd1)*. RNAi knockdown of *prd1* via *ppk-Gal4* caused prominent dendrite pruning defects in ddaC neurons at 16 h APF (v108557, *n* = 15, Fig 1C, 1I and 1J; v40070, *n* = 21, Fig 1I and 1J). In contrast, at the same time point, larval dendrites were completely removed in the control neurons (*n* = 25; Fig 1B, 1I and 1J). *prd1* encodes a previously uncharacterized protein with 1,354 amino acids, which contains a pleckstrin homology (PH) domain at its C-terminal portion (S1A Fig). Database searches revealed that in the *Drosophila* genome, Prd1 is most closely related to mammalian SKIP/PLEKHM2 (S1A Fig). They share amino acid sequence identity in their C-terminal portions, including their PH domains and the flanking regions (S1A Fig). SKIP was reported to regulate endosomal/lysosomal distribution in mammalian cells [25,26]. However, the function of *Drosophila* Prd1 was completely unknown.

To further verify the requirement of *prd1* for dendrite pruning, we took advantage of a *prd1*^*M56*^ mutant allele that was previously generated via flippase (FLP)-mediated recombination between two flippase recognition target (FRT)-containing P-element insertions (S1B Fig). It deletes the majority of the *prd1* coding region (aa329–1,354) (S1B Fig) and hence is a strong hypomorphic allele. Mutants hemizygous for *prd1*^*M56*^ and a small deletion *Df(3R)Exel7310* (deleting the entire *prd1* gene and its neighboring genes) died at the pharate adult stage and exhibited prominent dendrite pruning defects in ddaC neurons at 16 h APF. A total of 96% of those hemizygous mutant neurons exhibited dendrite severing defects and retained their larval dendrites with the attachment to their cell bodies by 16 h APF (*n* = 24; Fig 1D, 1I and 1J). These dendrite pruning defects in the mutant pupae hemizygous for *prd1*^*M56*^ and *Df(3R)Exel7310* were fully rescued by ectopic expression of full-length Prd1 (*n* = 22; Fig 1E, 1I and 1J) or Venus-tagged Prd1 (*n* = 19; Fig 1F, 1I and 1J). By mosaic analysis with a repressible cell marker (MARCM) analyses in ddaC neurons, another allele, *prd1*^*PS1*^ (S1B Fig), phenocopied *prd1*^*M56*^/*Df(3R)Exel7310* mutants. All *prd1*^*PS1*^ mutant ddaC neurons failed to prune away their larval dendrites (*n* = 11; Fig 1G, S1D Fig) and exhibited 91% of severing defects and 9% of fragmentation defects (Fig 1I). The length of unpruned dendrites in *prd1*^*PS1*^ mutants is comparable to that in hemizygous mutants of *prd1*^*M56*^ and *Df(3R)Exel7310* (Fig 1J). Importantly, ectopic expression of full-length Prd1 also fully rescued their dendrite pruning defects in *prd1*^*PS1*^ mutant ddaC clones (*n* = 12; Fig 1J), confirming that the dendrite pruning defects in *prd1*^*PS1*^ mutant neurons are caused by loss of *prd1* function.

The number of the primary and secondary dendrites in *prd1*^*M56*^/*Df(3R)Exel7310* (20.6 ± 0.33, *n* = 10; Fig 1D) remained similar to that of the control at the white prepupal (WP) stage (21.7 ± 0.16, *n* = 10; Fig 1B). *prd1*^*M56*^ MARCM ddaC clones showed slightly simplified dendrite arbors (S1C Fig). In addition to ddaC neurons, wild-type ddaD/E sensory neurons also completely pruned away their larval dendrites by 20 h APF (*n* = 12; S2A Fig). *prd1*^*M56*^ ddaD/E clones retained some of their larval dendrites attached to their soma (89%, *n* = 9; S2A Fig). Moreover, wild-type ddaF neurons are apoptotic during early metamorphosis. Interestingly, ddaF neurons derived from *prd1*^*M56*^ MARCM clones were eliminated (*n* = 5; S2B Fig), similar to wild type (*n* = 3; S2B Fig), suggesting that *prd1* is dispensable for ddaF apoptosis.

Taken together, Prd1 is cell-autonomously required for dendrite pruning but dispensable for neuronal apoptosis in sensory neurons during early metamorphosis.

### Prd1 colocalizes with α-Ada/clathrin puncta in dendrites

**Fig 2:**
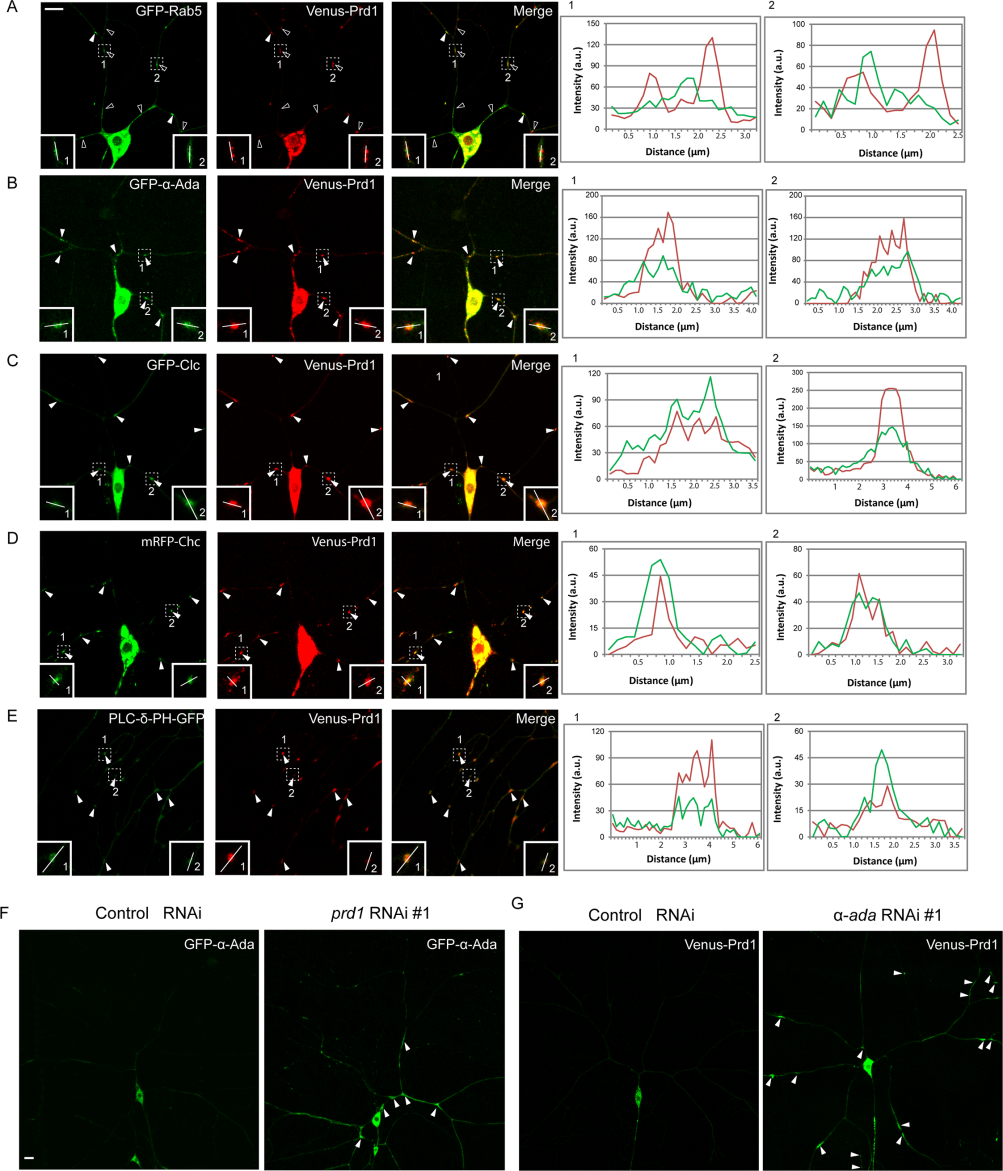
Prd1 colocalizes with α-Ada and clathrin in dendrites. (A) Distribution of Venus-Prd1 and GFP-Rab5 in ddaC neurons. Venus-Prd1 primarily localized adjacent to GFP-Rab5 (open arrowheads) (insets) and occasionally colocalized with GFP-Rab5 (arrowheads) in the dendrites. (B) Distribution of GFP-α-Ada and Venus-Prd1 in ddaC neurons. Venus-Prd1 colocalized with GFP-α-Ada in the dendrites (arrowheads). (C) Distribution of Venus-Prd1 and GFP-Clc in ddaC neurons. Venus-Prd1 colocalized with GFP-Clc in the dendrites (arrowheads). (D) Distribution of Venus-Prd1 and mRFP-Chc in ddaC neurons. Venus-Prd1 colocalized with mRFP-Chc in the dendrites (arrowheads). (E) Distribution of Venus-Prd1 and PLC-δ-PH-GFP in ddaC neurons. Venus-Prd1 colocalized with PLC-δ-PH-GFP in the dendrites (arrowheads). Line profiles show the arbitrary fluorescence intensity along the white lines. (F) Distribution of GFP-α-Ada in control and *prd1* RNAi ddaC neurons. (G) Distribution of Venus-Prd1 in control and α*-ada* RNAi ddaC neurons. Dorsal is up in all images. Scale bars in (A) and (F) represent 10 μm. The individual numerical values for panels A, B, C, D, and E can be found in S1 Data. The genotypes can be found in S1 Text. α-Ada, α-Adaptin; a.u., arbitrary unit; Chc, Clathrin heavy chain; Clc, Clathrin light chain; GFP, green fluorescent protein; mRFP, monomeric red fluorescent protein; PLC-δ-PH, phospholipase C-δ-pleckstrin homology; Prd1, pruning defect 1; Rab5, Rabaptin-5; RNAi, RNA interference.

To understand the functions of Prd1 in dendrite pruning, we examined its subcellular localization in ddaC neurons. Several antibodies were raised against three different portions of Prd1 (S3A Fig). Given that ddaC neurons are sandwiched between the epidermis and body wall muscles, endogenous Prd1 signals in the dendrites were masked by its ubiquitous expression in the surrounding tissues. We therefore generated the transgenes expressing Prd1 tagged with Venus fluorescent protein at its N-terminus (Venus-Prd1). The expression of Venus-Prd1, which was detected by the anti-Prd1 antibody (*n* = 11; S3B Fig), fully rescued dendrite pruning defects in *prd1*^*M56*^*/Df(3R)Exel7310* mutants (Fig 1F, 1I and 1J), suggesting that Venus-Prd1 functionally substitutes for endogenous Prd1. Venus-Prd1 was distributed in the soma, dendrites, and axons of ddaC neurons (*n* = 11; Fig 2G). Importantly, Venus-Prd1 also localized as some discrete puncta along the dendrites (Fig 2G). Mammalian SKIP/PLEKHM2 functions in the proper distribution of endosomes/lysosomes [25,26]. We then investigated whether Prd1-positive puncta represent endosomes or endocytic vesicles. To this end, we co-expressed Venus-Prd1 with various endocytic markers green fluorescent protein (GFP)-Rab5, GFP-α-Ada, and GFP-Clathrin light chain (Clc)/monomeric red fluorescent protein (mRFP)-Clathrin heavy chain (Chc) in ddaC neurons. Interestingly, Venus-Prd1 primarily localized adjacent to GFP-Rab5 (89%, *n* = 123 puncta, open arrowheads) (insets) and occasionally colocalized with GFP-Rab5 (11%, arrowheads) in the dendrites (Fig 2A). This result suggests that the majority of Prd1 puncta juxtapose to Rab5-positive early endosomes. Interestingly, Venus-Prd1 colocalized with GFP-α-Ada (88%, *n* = 116 puncta; Fig 2B, insets), GFP-Clc (89%, *n* = 102 puncta; Fig 2C, insets), and mRFP-Chc (93%, *n* = 88 puncta; Fig 2D, insets) in the dendrites of ddaC neurons (arrowheads). These data suggest that Prd1 may be a component of α-Ada/clathrin-positive structures. Moreover, in the dendrites of ddaC neurons, Venus-Prd1 puncta were also enriched with phospholipase C-δ-pleckstrin homology (PLC-δ-PH)-GFP (94%, *n* = 394 puncta, Fig 2E), a PtdIns(4,5)P_2_ sensor that indicates membrane regions with highly active endocytosis [27]. As a control, Venus-Prd1 puncta localized distinctly from the Golgi marker ADP ribosylation factor 79F fused with enhanced green fluorescent protein (Arf79F-EGFP) in the dendrites of ddaC neurons (81%, *n* = 194 puncta; S3C Fig, insets). Thus, Prd1 and α-Ada/clathrin colocalize at punctate spots in the dendrites where endocytosis appears to be highly active.

We next examined whether Prd1 regulates α-Ada localization in the dendrites. When *prd1* was knocked down via RNAi, GFP-α-Ada formed prominent aggregates in the dendrites of mutant neurons (*n* = 11, 45%; Fig 2F), compared with the control neurons (*n* = 11). Likewise, Venus-Prd1 often accumulated on several enlarged cellular aggregates in the dendrites of *α-ada* RNAi mutant neurons (*n* = 11; Fig 2G, arrowheads), compared with the control neurons (*n* = 11; Fig 2G). These results suggest that Prd1 and α-Ada are mutually required for their distributions in the dendrites. We previously reported that the expression of Rab5^DN^ or Vacuolar protein sorting-associated protein 4 (Vps4)^DN^ led to robust accumulation of ubiquitinated protein on enlarged endosomes in ddaC neurons [23]. Interestingly, Venus-Prd1 signals were also enriched on enlarged ubiquitin-positive endosomes in Rab5^DN^- or Vps4^DN^-expressing ddaC neurons (*n* = 12 and 12, respectively; S4A and S4B Fig), in contrast to the control (*n* = 8; S4A and S4B Fig). Similar to Prd1, α-Ada and Clc also accumulated on the aberrant endosomes in *Rab5*^*DN*^ (*n* = 14 and 15, respectively) or *Vps4*^*DN*^ (*n* = 12 and 8, respectively) mutant ddaC neurons (S4A and S4B Fig). As controls, overexpression of either Rab5^DN^ or Vps4^DN^ did not affect the distribution of mitochondria (Mito-GFP; *n* = 13 and 5, respectively), Golgi (GM130; *n* = 10 and 3, respectively), and endoplasmic reticulum (ER) (KDEL; *n* = 4 and 5, respectively) in ddaC neurons (S5 Fig).

Collectively, Prd1 predominantly colocalizes with α-Ada/clathrin-positive puncta in the dendrites and its distribution requires the endocytic regulators α-Ada, Rab5, and Vps4.

### Prd1 physically associates with α-Ada but not Rab5 in S2 cells

**Fig 3:**
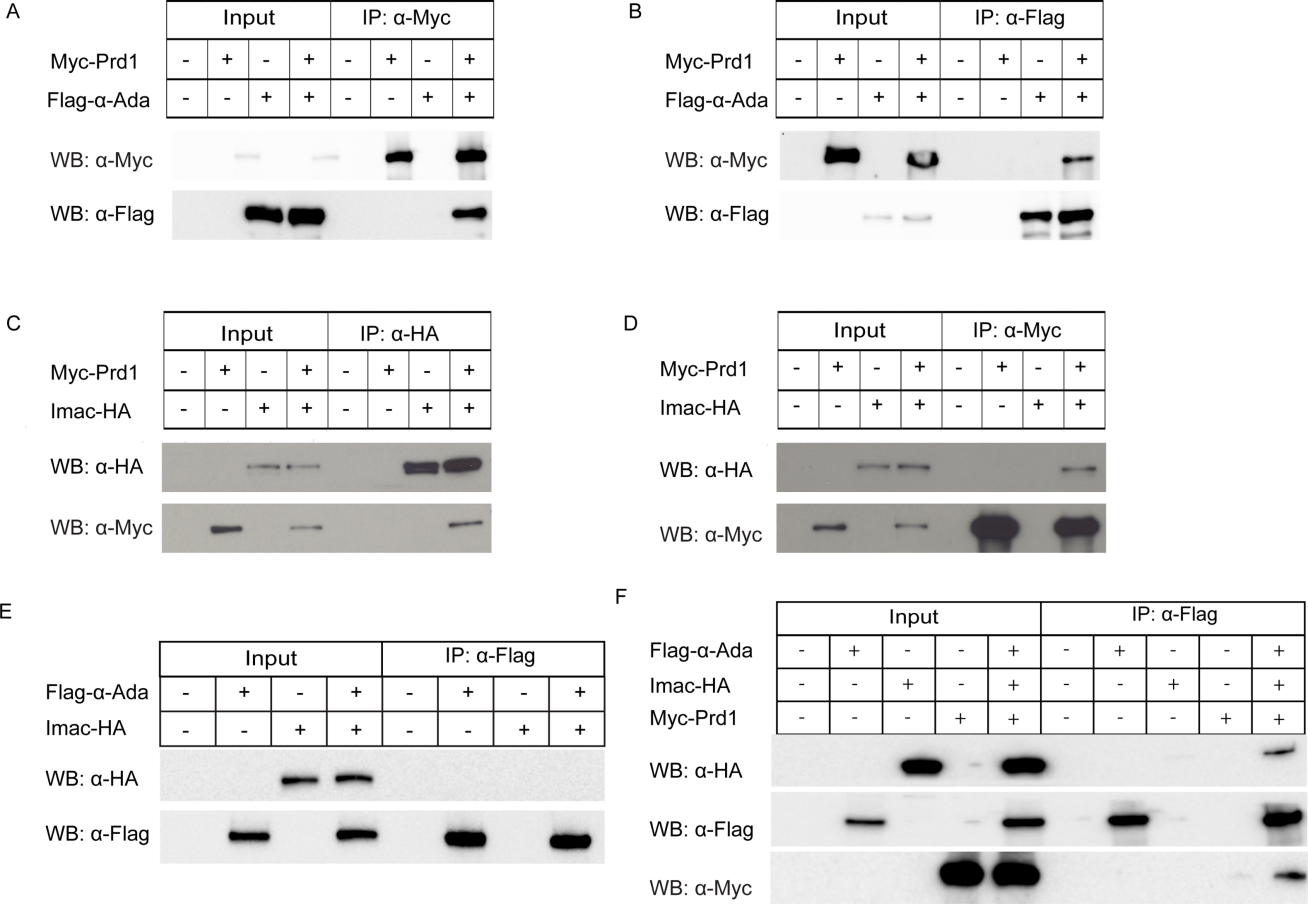
Prd1 forms a protein complex with α-Ada and Imac in S2 cells. (A-B) Co-IP between Prd1 and α-Ada. Prd1 and α-Ada associated with each other in S2 cells co-transfected with Myc-Prd1 and Flag-α-Ada. (C–D) Co-IP between Prd1 and Imac. Myc-Prd1 formed a protein complex with Imac-HA in reciprocal co-IP experiments when cell extracts were immunoprecipitated with anti-HA (C) or anti-Myc (D) antibody. (E) Co-IP between α-Ada and Imac. α-Ada did not associate with Imac in S2 cells co-transfected with Flag-α-Ada and Imac-HA. (F) Co-IP for Flag-α-Ada, Imac-HA, and Myc-Prd1. Imac was co-immunoprecipated by α-Ada when Flag-α-Ada, Imac-HA, and Myc-Prd1 were co-transfected in S2 cells. α-Ada, α-Adaptin; α-Flag, anti-Flag antibody; α-HA, anti-HA antibody; α-Myc, anti-Myc antibody; co-IP, co-immunoprecipitation; HA, HA tag; IP, immunoprecipitation; Myc, Myc tag; Prd1, pruning defect 1; WB, western blot.

Given their close localization patterns, we next attempted to examine potential protein–protein interactions between Prd1 and α-Ada/Rab5. To this end, we co-transfected S2 cells and conducted co-immunoprecipitation (co-IP) experiments. α-Ada was present specifically in the immune complex when Prd1 was immunoprecipitated using an anti-Myc antibody (Fig 3A). Reciprocally, Prd1 was also co-immunoprecipitated in the α-Ada complex using an anti-Flag antibody (Fig 3B). The β subunit of AP-2 is Bap (β-Adaptin, also known as *AP-2β* or *AP-1-2β*), which is probably shared between Adaptor protein-1 (AP-1) and AP-2 complexes in *Drosophila* [28]. Similar to the association with α-Ada, Prd1 also formed a complex with Bap in S2 cells co-transfected with Myc-Prd1 and Flag-Bap (S6A and S6B Fig). In contrast, Prd1 did not present in the same immune complex with Rab5 in both directions of co-IP experiments (S7A and S7B Fig). Thus, Prd1 forms a protein complex with α-Ada and Bap, rather than with Rab5.

### α-Ada, like Prd1, is critical for dendrite pruning

**Fig 4:**
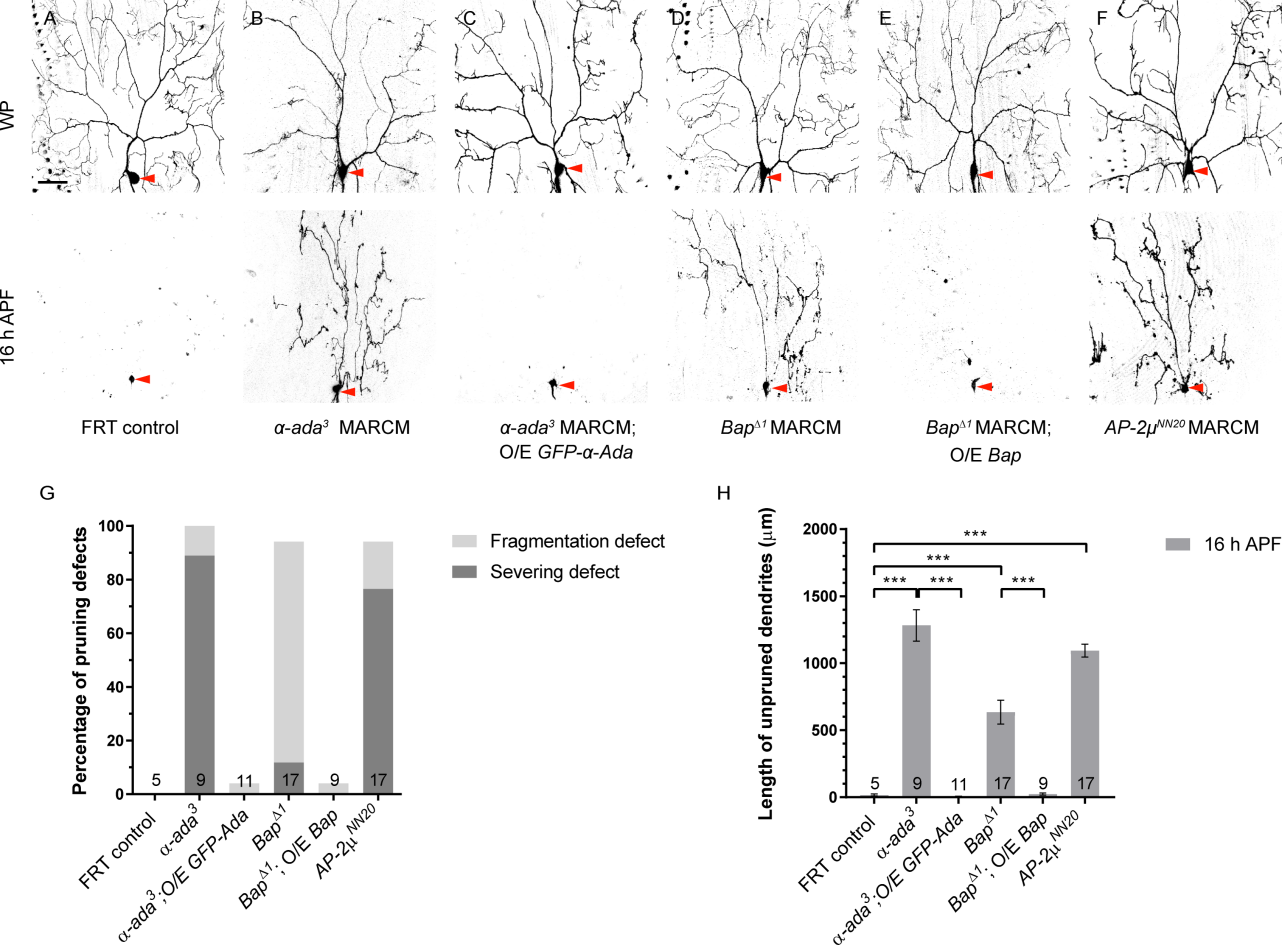
α-Ada and other AP-2 subunits are critical for dendrite pruning in ddaC neurons. (A–F) Live confocal images of control (A), *α-ada*^*3*^ MARCM (B), *α-ada*^*3*^ MARCM rescue (C), *Bap*^*Δ1*^ MARCM (D), *Bap*^*Δ1*^ MARCM rescue (E), and *AP-2μ*^*NN20*^ MARCM (F) ddaC neurons at WP and 16 h APF. ddaC somas are indicated by red arrowheads. (G) Quantification of percentage of severing defect and fragmentation defect in control and mutant ddaC neurons at 16 h APF. (H) Quantification of total length of unpruned dendrites at 16 h APF. The number of samples (*n*) in each group is shown on the bars. Error bars represent SEM. Scale bar (A) represents 50 *μ*m. ***p < 0.001 as assessed by one-way ANOVA test. The individual numerical values for panels G and H can be found in S1 Data. The genotypes can be found in S1 Text. α-Ada, α-Adaptin; α-*ada*^*3*^, α-*adaptin*^*3*^; *AP-2μ*^*NN20*^, *adaptor protein-2 μ subunit*^*NN20*^; APF, after puparium formation; *Bap*^*Δ1*^, *β-adaptin*^*Δ1*^; FRT, flippase recognition target; GFP, green fluorescent protein; MARCM, mosaic analysis with a repressible cell marker; O/E, overexpression; WP, white prepupal.

To assess a functional link between α-Ada and Prd1, we then investigated whether α-Ada, like Prd1, plays a role in dendrite pruning. We first knocked down *α-ada* gene function in ddaC neurons by *ppk-Gal4* driver via RNAi. Two *α-ada* RNAi lines, v15566 (#1) and BL#32866 (#2), caused consistent dendrite pruning defects in ddaC neurons by 16 h APF (*n* = 13 and 30, respectively; S8 Fig). Second, using a previously reported null allele α-*adaptin*^*3*^ (*α-ada*^*3*^) [29], we generated its homozygous MARCM clones to verify its requirement for dendrite pruning. Importantly, all *α-ada*^*3*^ mutant ddaC clones exhibited strong dendrite pruning defects, including 89% of severing defect at 16 h APF (*n* = 9; Fig 4B, 4G and 4H), in contrast to the control neurons (*n* = 5; Fig 4A, 4G and 4H). *α-ada*^*3*^ mutant neurons also showed notable dendrite morphology defects and their dendrite arbors were simplified at the WP stage (*n* = 9; Fig 4B), similar to that reported in *α-ada* RNAi knockdown [20]. These dendritic defects were fully rescued by the expression of GFP-α-Ada under the control of *ppk-Gal4* (*n* = 11; Fig 4C, 4G and 4H). Similarly, ddaD/E MARCM clones homozygous for *α-ada*^*3*^ also failed to prune their larval dendrites by 20 h APF (89%, *n* = 9; S9A Fig). ddaF neurons derived from *α-ada*^*3*^ mutants were eliminated (*n* = 2; S9B Fig), similar to control neurons (*n* = 3; S9B Fig). We observed that α-Ada was expressed in both ddaF and ddaC neurons of wild-type larvae (*n* = 8; S9D Fig). Thus, α-Ada, like Prd1, is required for dendrite pruning of sensory neurons but not for neuronal apoptosis.

We next examined other AP-2 subunits for their potential involvement in dendrite pruning. We generated *Bap*^*Δ1*^, an imprecise excision allele for *Bap* (S9C Fig) that encodes the β subunit of the AP-2 complex. *Bap*^*Δ1*^ deletes a small C-terminal part of the coding region (aa861–921) as well as the whole 3′-UTR region, suggesting a hypomorphic allele. Mutant clones homozygous for *Bap*^*Δ1*^ showed mild pruning defects (*n* = 17; Fig 4D, 4G and 4H), probably because of a weak allele or perdurance of the wild-type protein in mutant clones. These pruning defects were fully rescued by the expression of an upstream activating sequence (*UAS*)*-Bap* transgene (*n* = 9; Fig 4E, 4G and 4H). Importantly, AP-2μ, the μ subunit of AP-2 complex, is also important for ddaC dendrite pruning. ddaC clones homozygous for *AP-2*μ^*NN20*^, a null allele with the M1I mutation [30], showed severe dendrite pruning defects with many larval dendrites attached (*n* = 17; Fig 4F, 4G and 4H), to an extent similar to *α-ada*^*3*^ mutant clones. Moreover, we examined the potential requirement of *AP-1* genes (*AP-1*μ and *AP-1γ)* for dendrite pruning using the loss-of-function allele *AP-1*μ^*SHE-11*^ [31] and *AP-1γ*^*B*^/*AP-1γ*^D^ mutants [32]. No dendrite pruning defects were observed in ddaC clones of *AP-1*μ^*SHE-11*^ and *AP-1γ*^*B*^/*AP-1γ*^D^ mutants (*n* = 6, 9, and 4, respectively; S10 Fig). Thus, these data suggest that Prd1 most likely regulates dendrite pruning via AP-2 but independently of AP-1.

Collectively, α-Ada and other AP-2 subunits are critical for dendrite pruning in sensory neurons, whereas α-Ada is dispensable for neuronal apoptosis. Therefore, α-Ada and Prd1 play similar roles in dendrite pruning but not in neuronal apoptosis during metamorphosis.

### Prd1 colocalizes with a neuronal kinesin Imac and links α-Ada to Imac

**Fig 5:**
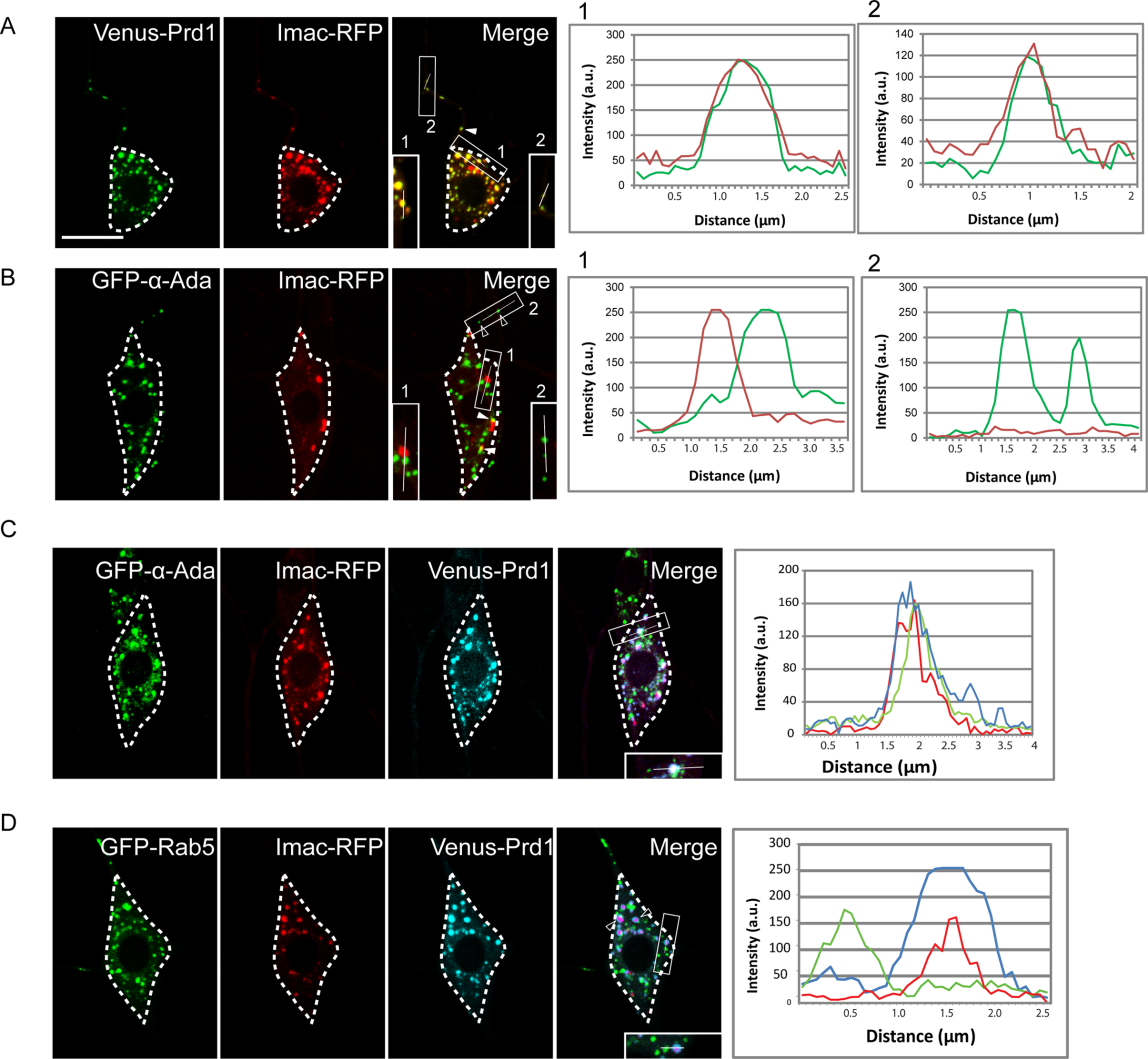
Prd1 colocalizes with Imac and brings α-Ada to Imac. (A) Distribution of Prd1 and Imac in ddaC neurons expressing Venus-Prd1 and Imac-RFP. Venus-Prd1 colocalized with Imac-RFP in the soma and dendrites (arrowheads). (B) Distribution of α-Ada and Imac in ddaC neurons expressing GFP-α-Ada and Imac-RFP. GFP-α-Ada did not overlap with Imac-RFP in the dendrites (open arrowheads). (C) Distribution of Prd1, α-Ada, and Imac in ddaC neurons expressing GFP-α-Ada, Venus-Prd1, and Imac-RFP. Venus-Prd1, GFP-α-Ada, and Imac-RFP colocalized in ddaC soma and dendrites. (D) Distribution of Prd1, Rab5, and Imac in ddaC neurons expressing GFP-Rab5, Venus-Prd1, and Imac-RFP. GFP-Rab5 spots were juxtaposed with Venus-Prd1/Imac-RFP puncta in ddaC neurons. Line profiles show the arbitrary fluorescence intensity along the white lines. ddaC somas are depicted by dashed lines. Scale bar (A) represents 10 μm. The individual numerical values for panels A, B, C, and D can be found in S1 Data. The genotypes can be found in S1 Text. α-Ada, α-Adaptin; a.u., arbitrary unit; GFP, green fluorescent protein; Prd1, pruning defect 1; Rab5, Rabaptin-5; RFP, red fluorescent protein.

Prd1-related mammalian SKIP/PLEKHM2 binds to kinesin-1 and activates it to regulate the distribution of endosomes/lysosomes [25,26]. To investigate whether Prd1 regulates the distribution of α-Ada puncta via a motor protein, we examined potential colocalization between Prd1 and various motor proteins, including kinesin-1, 2, and 3 and dynein. Venus-Prd1 was distributed in a punctate pattern, which is distinct from the GFP-tagged Kinesin heavy chain (Khc-GFP) puncta (*n* = 11; S11A Fig), suggesting that unlike SKIP, Prd1 may function independently of kinesin-1 in *Drosophila*. Neither did Prd1 colocalize with Kinesin associated protein 3 (Kap3), a subunit of the heterotrimeric kinesin-2 motor (*n* = 24; S11B Fig). Remarkably, when Venus-Prd1 was co-expressed with red fluorescent protein (RFP)-tagged Imac (Imac-RFP), a key neuronal kinesin-3, Prd1 fully colocalized with Imac-RFP in the dendrites (arrowheads) and soma of ddaC neurons (94%, *n* = 462 puncta; Fig 5A). Moreover, we did not observe a similar distribution pattern between Prd1 and Dynein light intermediate chain (Dlic) or the dynein regulator Lis1 (*n* = 5 and 11, respectively; S11C and S11D Fig). Thus, Prd1 appears to specifically colocalize with kinesin-3, but not with kinesin-1 and 2 or dynein, in the dendrites of ddaC sensory neurons.

Because Venus-Prd1 colocalized with GFP-α-Ada and Imac-RFP in ddaC neurons, respectively, we expected a colocalization of GFP-α-Ada with Imac-RFP. Surprisingly, GFP-α-Ada did not colocalize with Imac-RFP in the dendrites (open arrowheads), although their puncta occasionally juxtaposed in the soma (arrowheads) (7%, *n* = 99 puncta; Fig 5B). One possible explanation is that under the above experimental condition, in which both GFP-α-Ada and Imac-RFP were overexpressed, the low level of endogenous Prd1 protein is insufficient to bring GFP-α-Ada and Imac-RFP together. Remarkably, when Venus-Prd1 was co-expressed with GFP-α-Ada and Imac-RFP in ddaC neurons, GFP-α-Ada colocalized with Venus-Prd1 and Imac-RFP (79%, *n* = 527 puncta; Fig 5C). This result indicates that Prd1 may mediate the association between α-Ada and Imac in ddaC neurons. Likewise, Venus-Prd1 also colocalized with GFP-α-Ada and Imac-RFP in the axons (S12A–S12D Fig). By contrast, overexpressed Venus-Prd1 failed to affect Rab5 localization in ddaC neurons, which was juxtaposed with Imac/Prd1 puncta (91%, *n* = 207 puncta; Fig 5D, open arrowheads).

Thus, our results support the conclusion that Prd1 mediates the association of α-Ada with Imac in ddaC sensory neurons.

### Imac plays a crucial role in dendrite pruning

**Fig 6:**
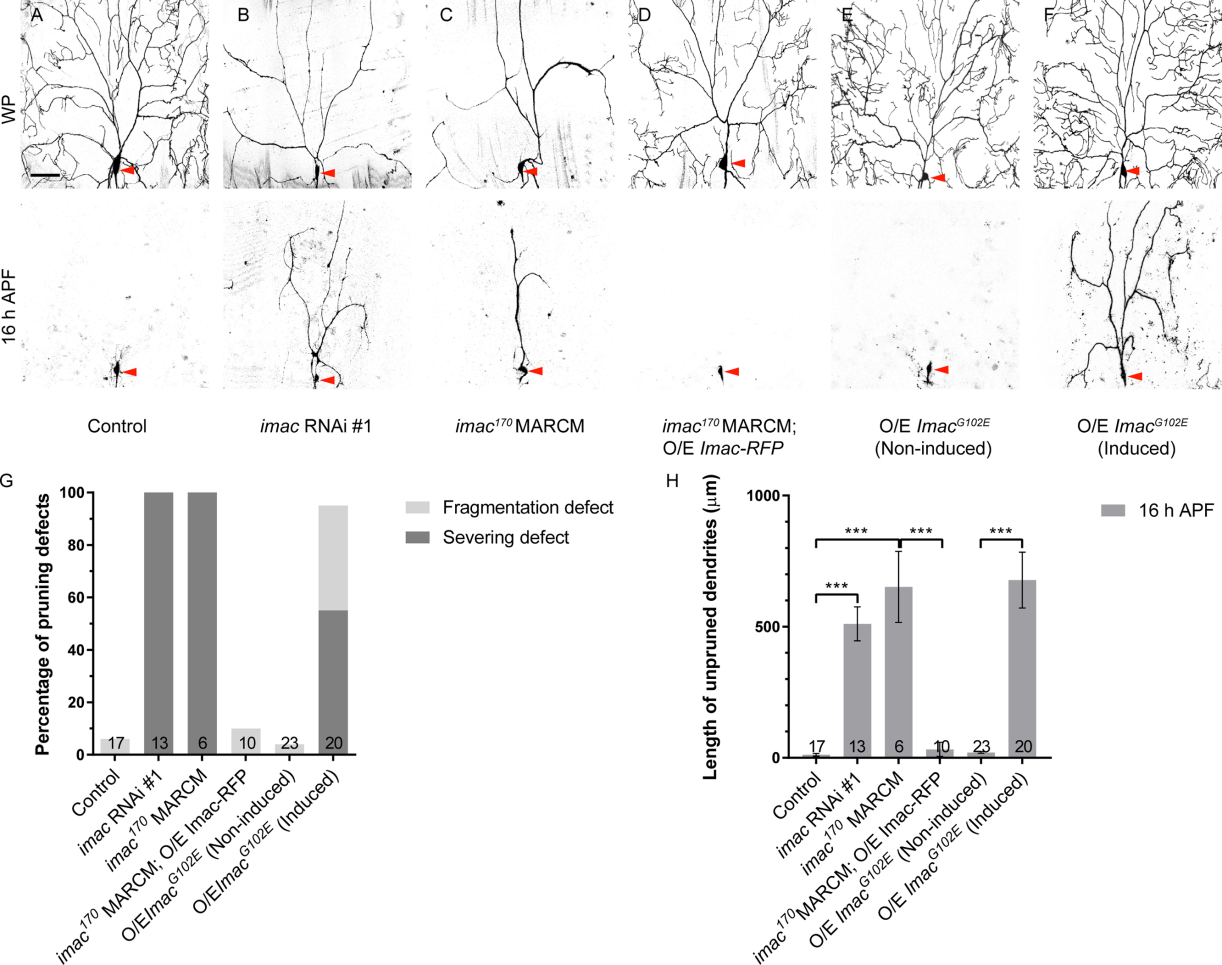
Imac plays a crucial role in dendrite pruning of ddaC neurons. (A–F) Live confocal images of control (A), *imac* RNAi #1 (B), *imac*^*170*^ MARCM (C), *imac*^*170*^ MARCM rescue (D), non-induced *Imac*^*G102E*^ (E), and induced *Imac*^*G102E*^ (F) ddaC neurons at WP and 16 h APF. ddaC somas are marked by red arrowheads. (G) Quantification analysis of percentage of severing defect and fragmentation defect in control and *imac* mutant ddaC neurons at 16 h APF. (H) Quantification of total length of unpruned dendrites at 16 h APF. The number of samples (*n*) in each group is shown on the bars. Error bars represent SEM. Scale bar (A) represents 50 μm. ***p < 0.001 as assessed by one-way ANOVA test. The individual numerical values for panels G and H can be found in S1 Data. The genotypes can be found in S1 Text. APF, after puparium formation; *imac*, *immaculate connections*; MARCM, mosaic analysis with a repressible cell marker; O/E, overexpression; RNAi, RNA interference; WP, white prepupal.

Because the role of Imac in dendrite pruning is unknown, we next explored whether knockdown or loss of *imac* function caused dendrite pruning defects. Two independent *imac* RNAi lines, v47171 (#1) and v23465 (#2), when expressed in ddaC neurons via *ppk-Gal4*, led to severe dendrite pruning defects in all ddaC neurons at 16 h APF (*n* = 13 and 5, respectively; Fig 6B, S14 Fig). In contrast, the control neurons showed normal dendrite pruning at 16 h APF (*n* = 17; Fig 6A). Moreover, using the previously reported null allele *imac*^*170*^ [33], we generated their ddaC mutant clones and analyzed their defects in dendrite pruning at 16 h APF. ddaC clones homozygous for *imac*^*170*^ exhibited strong dendrite pruning defects by 16 h APF, with full penetrance (*n* = 6, respectively; Fig 6C, 6G and 6H). Similar to that reported in a previous RNAi study [34], we also observed severe dendrite arborization defects in *imac* RNAi neurons and mutant clones. Only major dendrites were still present in the vicinity of mutant ddaC soma at the WP stage (*n* = 11 and 6, respectively; Fig 6B and 6C). Both dendrite morphology and pruning defects in *imac*^*170*^ mutant neurons were fully rescued by the expression of Imac-RFP (*n* = 10; Fig 6D, 6G and 6H), suggesting that Imac-RFP functionally substitutes for endogenous Imac. Thus, Imac is required for both pruning and growth of dendrites in ddaC neurons.

Like nematode Unc-104, Imac contains a motor domain, three coiled coil domains, a forkhead-associated domain, and a PH domain (S13 Fig). We identified a conserved ATP-binding sequence (GQTGAGKS) within the motor domain of the Imac protein and generated two mutant forms, GQTGAEKS (Imac^G102E^) and GQTGAAAA (Imac^AAA^) (S13 Fig), which were reported to disrupt the motor activity of kinesins and myosins in other organisms [35]. We found that both *Drosophila* Imac^G102E^ and Imac^AAA^ behaved as dominant-negative forms, because their overexpression phenocopied *imac* loss-of-function mutants in terms of both dendrite arborization and pruning defects (*n* = 13 and 7, respectively; S14 Fig compared to Fig 6B and 6C). These data support the conclusion that the kinesin motor activity of Imac plays a crucial role in dendrite arborization and pruning.

To rule out the possibility that the *imac*-associated dendrite pruning defects are secondary to the initial dendrite arborization defect, we conducted the Gene-Switch experiments by inducing the expression of these two dominant-negative forms at the early third instar larval stage (72 h after egg laying [AEL]). The Gene-Switch manipulations enabled mutant ddaC neurons to arborize mature and complex larval dendrites, as shown at the WP stage (*n* = 12 and *n* = 8, respectively; Fig 6F, S15 Fig), similar to their respective controls (*n* = 9; Fig 6E, S15 Fig). Importantly, dendrite pruning defects were consistently observed upon the expression of Imac^G102E^ (*n* = 20; Fig 6F) or Imac^AAA^ (*n* = 22; S15 Fig) in ddaC neurons via the Gene-Switch driver *GSG2295-Gal4* at 16 h APF, in contrast to no pruning defect observed in either non-induced (*n* = 23; Fig 6E) or induced controls (*n* = 12; S15 Fig). Thus, the Gene-Switch experiments highlight that *imac*-associated dendrite pruning defects are not a secondary effect of dendrite arborization defect.

Taken together, multiple lines of genetic evidence demonstrate that the *Drosophila* kinesin-3 Imac is a crucial motor protein regulating dendrite pruning in ddaC sensory neurons.

### Imac regulates α-Ada/clathrin distribution and endo-lysosomal degradation

**Fig 7:**
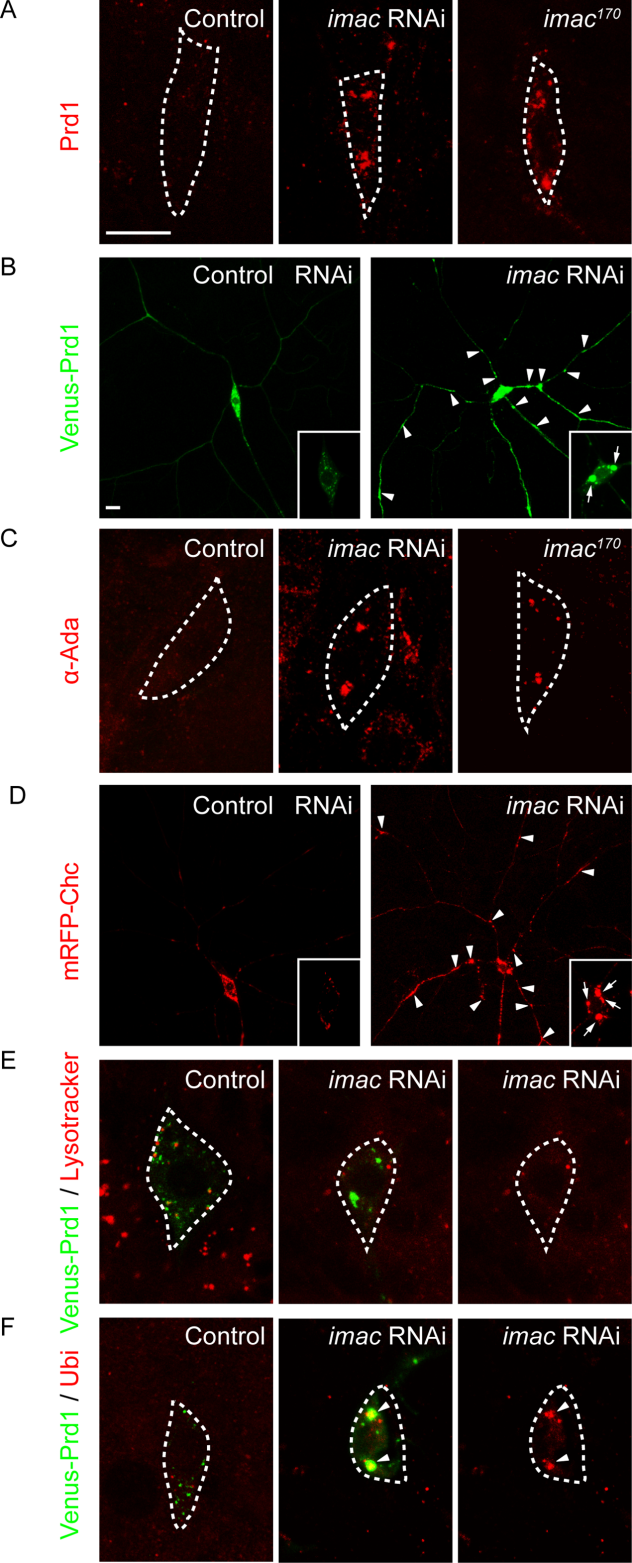
Imac regulates α-Ada/clathrin distribution and endo-lysosomal degradation. (A) Distribution of endogenous Prd1 (in red) in control, *imac* RNAi, and *imac*^*170*^ MARCM ddaC neurons. (B) Distribution of Venus-Prd1 (in green) in control and *imac* RNAi (#1) ddaC neurons expressing Venus-Prd1. (C) Distribution of endogenous α-Ada (in red) in control, *imac* RNAi (#1), and *imac*^*170*^ MARCM ddaC neurons. (D) Distribution of mRFP-Chc (in red) in control and *imac* RNAi (#1) ddaC neurons expressing mRFP-Chc. (E) Distribution of LysoTracker (in red) and Venus-Prd1 (in green) in control and *imac* RNAi (#1) ddaC neurons. (F) Distribution of ubiquitinated proteins (anti-Ubiquitin) (in red) and Venus-Prd1 (in green) in control and *imac* RNAi (#1) ddaC neurons. Somas of ddaC are marked by dashed lines. Scale bars in (A) and (B) represent 10 μm. The genotypes can be found in S1 Text. α-Ada, α-Adaptin; Chc, Clathrin heavy chain; *imac*, *immaculate connections*; MARCM, mosaic analysis with a repressible cell marker; mRFP, monomeric red fluorescent protein; Prd1, pruning defect 1; RNAi, RNA interference; Ubi, ubiquitinated proteins.

We next examined whether *imac* is important for the distributions of Prd1, α-Ada, and clathrin in ddaC neurons. First, we examined the distribution of the endogenous Prd1 protein with an anti-Prd1 antibody in *imac* knockdown or mutant neurons. In contrast to weak punctate signals of Prd1 in the control soma (*n* = 11; Fig 7A), Prd1 accumulated on several bright aggregates in either *imac* RNAi or *imac*^*170*^ mutant neurons (*n* = 11 and 7, respectively; Fig 7A), suggesting that Imac promotes discrete distribution of Prd1 puncta in ddaC neurons. To better visualize its distribution in the dendrites, we expressed Venus-Prd1 and compared its distributions in control and *imac* RNAi ddaC neurons. In *imac* RNAi ddaC neurons, Venus-Prd1 strongly accumulated on many aggregates in the dendrites (#1, *n* = 21; control, *n* = 10; Fig 7B). Similar to the endogenous Prd1 protein (Fig 7A), Venus-Prd1 was also observed to form several large aggregates in the soma of *imac* RNAi neurons (#1, *n* = 21, Fig 7B, insets; #2, *n* = 10, S16 Fig), compared to the control neurons (*n* = 10; Fig 7B, S16 Fig). Likewise, using a previously reported anti-α-Ada antibody [29], we observed that endogenous α-Ada also aggregated on several large puncta in *imac* RNAi or *imac*^*170*^ mutant soma (*n* = 9 and 9, respectively; control: *n* = 12; Fig 7C). When overexpressed in *imac* RNAi ddaC neurons (#1 and #2), GFP-α-Ada, like the endogenous protein, strongly accumulated on the aggregates (*n* = 8 and 12, respectively; S16 Fig). Moreover, mRFP-Chc also accumulated as more aggregates in the dendrites and soma of *imac* RNAi ddaC neurons (*n* = 13, Fig 7D), compared to its distribution with small discrete puncta in control RNAi neurons. In contrast, we did not observe any obvious aggregates of Venus-Prd1 and α-Ada in *khc* RNAi ddaC neurons (*n* = 6 and 19, respectively; S17 Fig). These findings indicate that the kinesin-3 Imac, rather than kinesin-1, plays an important role in distributing Prd1, α-Ada, and clathrin in the dendrites of ddaC neurons.

Given that Imac regulates the distributions of α-Ada/clathrin puncta, we further examined whether loss of *imac* function impairs endosomal distribution and lysosomal degradation. In *imac* RNAi ddaC neurons, the Venus-Prd1 aggregates were enriched with the endosomal markers anti–hepatocyte growth factor-regulated tyrosine kinase substrate (Hrs) (*n* = 6; S18A and S18D Fig) and Rab5-GFP (*n* = 8; S18B and S18D Fig) in the dendrites and soma, suggesting that these Venus-Prd1 structures are aberrant endosomes. We next examined whether these aberrant endosomes can fuse with lysosomes to undergo protein degradation. To this end, we utilized the LysoTracker dye to label highly acidified lysosomal compartments that normally fuse with endosomes to degrade the ubiquitinated proteins. The LysoTracker dye labeled discrete lysosomes in control ddaC neurons (*n* = 16; Fig 7E). However, the Lysotracker dye was not enriched on aberrant endosomes in *imac* RNAi mutant neurons (*n* = 10; Fig 7E), suggesting a compromise in endosomal acidification and maturation. Consistently, ubiquitinated proteins, which normally exhibited weak punctate structures in control neurons (*n* = 12, Fig 7F), were strongly enriched on Venus-Prd1 aggregates in *imac* RNAi neurons (*n* = 8; Fig 7F), suggesting impaired endo-lysosomal degradation. As controls, in *imac* RNAi ddaC neurons, the secretory vesicle marker Sec15 did not accumulate as aggregates (*n* = 4; S18C and S18D Fig) and the Golgi marker β1,4-galactosyltransferase (GalT)-GFP appeared to be normal in size and distribution in the soma and dendrites (*n* = 6; S18E Fig).

The L1-CAM Nrg is endocytosed and down-regulated via the endo-lysosomal degradation pathway, leading to dendrite pruning in ddaC neurons [23]. We next examined whether Prd1, α-Ada, and Imac regulate endo-lysosomal degradation of Nrg before the onset of dendrite pruning. In wild-type ddaC neurons, Nrg protein levels were significantly decreased in the somas, dendrites, and axons (*n* = 23; S19A and S19E Fig) at 6 h APF. Importantly, the Nrg protein accumulated dramatically in the somas, dendrites, and axons of *prd1*^*M56*^ /*Df(3R)Exel7310* (*n* = 23; S19B and S19E Fig), *α-ada*^*3*^ MARCM (*n* = 13, S19C and S19E Fig), or *Imac*^*G102E*^ (*n* = 9; S19D and S19E Fig) ddaC neurons, similar to those in *Rab5*^*DN*^ mutant neurons. These data indicate that Prd1, α-Ada, and Imac are required to promote Nrg endo-lysosomal degradation prior to dendrite pruning. Moreover, the expression of an *nrg* RNAi line, which has been shown to efficiently knock down its protein [23], significantly suppressed the pruning defects of *prd*^*M56*^/*prd1*^*PS2*^ (*n* = 25; S20A Fig), *α-ada*^*3*^ MARCM (*n* = 13, S20B Fig), or *imac RNAi* (*n* = 11; S20C Fig) mutant ddaC neurons. Thus, Prd1, α-Ada, and Imac act to promote dendrite pruning at least partly through global endo-lysosomal degradation of Nrg. Moreover, a previous study has reported that local endocytosis leads to the thinning of proximal dendrites and compartmentalized calcium transients [22]. We next investigated whether Prd1/Imac/Unc-104 regulates local calcium transients at 6.5 h APF. While compartmentalized Ca^2+^ transients were present in the vast majority of control neurons (*n* = 21; S21 Fig), the percentage of ddaC neurons with Ca^2+^ transients at 6 h APF was drastically reduced in *prd1*^*M56*^*/Df(3R)Exel7310* (*n* = 20) and *Imac*^*G102E*^ (*n* = 13) ddaC neurons (S21 Fig). These data suggest that Prd1 and Imac are also required to regulate compartmentalized calcium transients before the onset of dendrite pruning.

Taken together, Imac is required for proper distributions of Prd1, α-Ada, and clathrin in the dendrites and facilitates lysosomal degradation of Nrg in ddaC sensory neurons.

### Prd1 forms a protein complex with Imac but not Khc in S2 cells

To further explore the mechanisms whereby Prd1 and Imac regulate dendrite pruning, we conducted co-IP experiments to assess their potential physical association. In S2 cells co-transfected with Myc-Prd1 and Imac-HA, Prd1 was pulled down when Imac was immunoprecipitated using an anti-HA antibody (Fig 3C). Reciprocally, Imac was also co-immunoprecipitated in the Prd1 immune complex (Fig 3D). These co-IP data, together with the colocalization results (Fig 5A), strongly support a functional link between *prd1* and *imac* during dendrite pruning. Moreover, in S2 cells co-transfected with Flag-α-Ada and Imac-HA, the association between Imac and α-Ada was not detectable (Fig 3E). Importantly, when Myc-Prd1 was co-transfected with Flag-α-Ada and Imac-HA, Imac was co-immunoprecipated by α-Ada (Fig 3F). Thus, α-Ada and Imac form a protein complex in the presence of Prd1 in S2 cells. As a control, consistent with distinct localizations of Prd1 and the kinesin-1 Khc, we did not observe their physical association in the reciprocal co-IP experiments (S22A and S22B Fig). These data suggest the selectivity of the interaction between Prd1 and the kinesin-3 Imac. Furthermore, Imac was not co-immunoprecipitated with Rab5 in reciprocal co-IP experiments (S22C and S22D Fig). Taken together, our biochemical and cell biological data imply that Prd1 might interact with Imac and recruit α-Ada to Imac in vivo to facilitate endo-lysosomal degradation in ddaC sensory neurons.

### *prd1*, *α-ada*, and *imac* genetically interact during dendrite pruning

**Fig 8:**
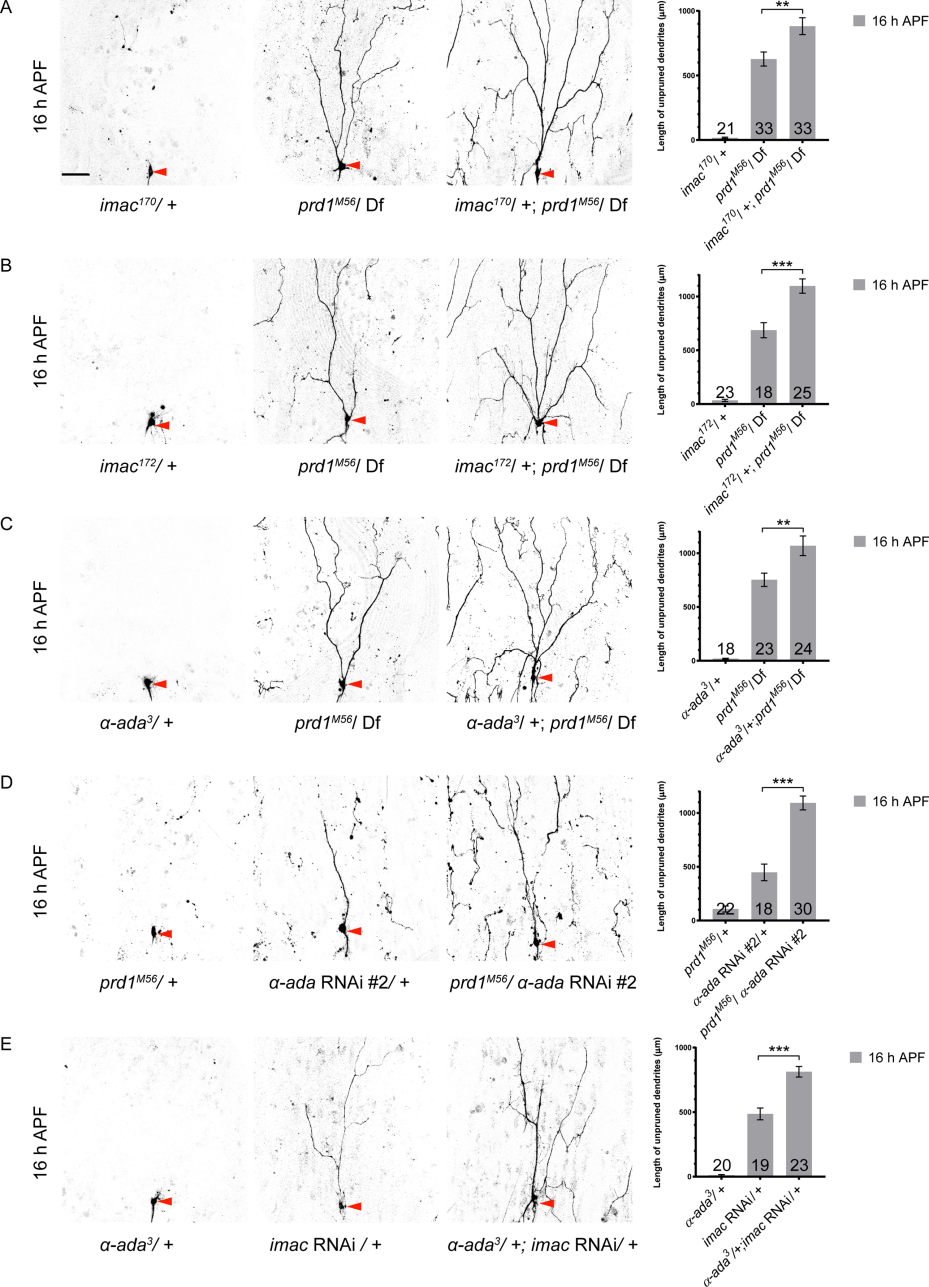
prd1, α-ada, and imac genetically interact during dendrite pruning in ddaC sensory neurons. (A) Dendrites of *imac*^*170*^*/+*, *prd1*^*M56*^*/Df(3R)Exel7310*, and *imac*^*170*^*/+; prd1*^*M56*^*/Df(3R)Exel7310* ddaC neurons at 16 h APF. (B) Dendrites of *imac*^*172*^*/+*, *prd1*^*M56*^*/Df(3R)Exel7310*, and *imac*^*172*^*/+; prd1*^*M56*^*/ Df(3R)Exel7310* ddaC neurons at 16 h APF. (C) Dendrites of *α-ada*^*3*^*/+*, *prd1*^*M56*^*/Df(3R)Exel7310*, and *α-ada*^*3*^*/+; prd1*^*M56*^*/Df(3R)Exel7310* ddaC neurons at 16 h APF. (D) Dendrites of *prd1*^*M56*^*/+*, *α-ada RNAi #2/+*, and *prd1*^*M56*^*/α-ada RNAi #2* ddaC neurons at 16 h APF. (E) Dendrites of *α-ada*^*3*^*/+*, *imac RNAi/+*, and *α-ada*^*3*^*/+; imac RNAi/+* ddaC neurons at 16 h APF. Quantification of total length of unpruned dendrites at 16 h APF. The number of samples (*n*) in each group is shown on the bars. Error bars represent SEM. Scale bar (A) represents 50 μm. **p < 0.01; ***p < 0.001. Dorsal is up in all images. The individual numerical values for panels A, B, C, D, and E can be found in S1 Data. The genotypes can be found in S1 Text. *α-ada*, *α-adaptin*; APF, after puparium formation; Df, deficiency; *imac*, *immaculate connections*; *prd1*, *pruning defect 1*; *RNAi*, RNA interference.

To further strengthen the functional link among Prd1, Imac, and α-Ada in dendrite pruning, we conducted various combinations of genetic interaction assays. At 16 h APF in ddaC neurons, while heterozygous *imac*^*170*^ or *imac*^*172*^ had no adverse effect on dendrite pruning (*n* = 21 and 23, respectively; Fig 8A and 8B), they significantly enhanced the pruning defects of *prd1*^*M56*^*/Df(3R)Exel7310* mutant neurons (*n* = 33 and 25, respectively; Fig 8A and 8B). More larval dendrite branches persisted in the vicinity of their soma than those in *prd1*^*M56*^*/Df(3R)Exel7310* mutants alone (*n* = 33 and 18, respectively; Fig 8A and 8B). By contrast, removal of one copy of *mical* or *cullin-1*, which are known to regulate dendrite pruning by a distinct mechanism [13,36], did not enhance the dendrite-pruning phenotypes of *prd1*^*M56*^*/Df(3R)Exel7310* mutants (S23A and S23B Fig).

Next, we explored the genetic interaction between *prd1* and *α-ada*. While the heterozygous *α-ada*^*3*^ allele did not show pruning defects (*n* = 18; Fig 8C), removal of one copy of *α-ada* (*α-ada*^*3*^*/+*) significantly enhanced the pruning phenotypes of *prd1*^*M56*^*/Df(3R)Exel7310* mutants (*n* = 24; Fig 8C). Moreover, one copy of *prd1*^*M56*^ allele (*prd1*^*M56*^*/+)*, which did not show notable pruning defects in the wild-type background (*n* = 22; Fig 8D), drastically enhanced the pruning phenotypes of *α-ada* RNAi mutants (*n* = 30; Fig 8D). Finally, removal of one copy of *α-ada* (*α-ada*^*3*^*/+*), which showed no pruning defect in wild-type background (*n* = 20), also caused a significant enhancement of the dendrite pruning defects in *imac* RNAi ddaC neurons (*n* = 23; Fig 8E). Thus, these genetic interaction results strongly support that Prd1, α-Ada, and Imac act in the same genetic pathway to promote dendrite pruning.

In summary, multiple lines of genetic, biochemical, and cell biological evidence support the model that Prd1, α-Ada, and Imac act in the same pathway to regulate discrete distribution of α-Ada/clathrin puncta, facilitate endo-lysosomal degradation of Nrg, and thereby promote dendrite pruning in sensory neurons (in S24 Fig).

## Discussion

Previous studies have demonstrated that endocytosis plays important roles in governing dendrite and axon pruning in ddaC and MB γ neurons, respectively [21–23]. However, the regulatory mechanism of endocytosis required for neuronal pruning is poorly understood. Moreover, kinesin motor proteins that regulate endocytosis during neuronal pruning are completely unknown. Here, we identified Prd1 and its associated proteins, the clathrin adaptor α-Ada and the kinesin-3 Imac, which all play important roles in dendrite pruning of ddaC neurons. Prd1 physically associates with α-Ada and Imac and colocalized with them in the dendrites. Similar to that in *prd1* mutant, loss of either *α-ada* or *imac* resulted in prominent dendrite pruning defects in ddaC neurons. Moreover, loss of *imac* function caused the formation of aberrant Prd1/α-Ada/clathrin aggregates in the dendrites as well as impaired endo-lysosomal degradation. Finally, genetic interaction results further support that *prd1*, *α-ada*, and *imac* act in the same pathway to promote dendrite pruning. Our study demonstrates that a clathrin adaptor–associated protein, Prd1, acts together with α-Ada and a kinesin-3 to regulate proper distribution of α-Ada/clathrin puncta and facilitate endo-lysosomal degradation during neuronal pruning.

### Prd1 regulates dendrite pruning

Growing evidence indicates that mammalian SKIP is involved in proper distribution of endosomes and lysosomes in mammalian cells. In infected cells, SKIP interacts with kinesin-1 to recruit the motor on the bacteria’s replicative vacuole, leading to the formation and distribution of late endosomes or lysosomes [37]. In uninfected cells, SKIP binds to the small GTPase Arf-like GTPase 8 (Arl8) through its RUN domain or to the late endosomal GTPase Rab9 via its PH domain to regulate lysosomal distribution in a kinesin-1-dependent fashion [25,26]. Moreover, a mutation in human PLEKHM2 caused aberrant accumulation of both early and late endosomes in patients’ fibroblast cells [38]. However, the physiological function of SKIP/PLEKHM2 remained unknown. In this study, we demonstrate that a previously uncharacterized *Drosophila SKIP*-related gene, *prd1*, plays a critical role in dendrite pruning.

Several lines of evidence support the notion that Prd1 regulates dendrite pruning via AP-2 complex. First, Prd1 colocalized with the AP-2 α subunit α-Ada and clathrin in the dendrites. Second, Prd1 physically associated with α-Ada but not Rab5. Third, Prd1 and α-Ada are mutually required for their proper distributions in the dendrites of ddaC neurons. Fourth, Prd1 distribution also requires the endocytic regulators Rab5 or Vps4. Prd1, similar to clathrin and the clathrin adaptor AP-2, was enriched on enlarged endosomes in *Rab5*^*DN*^ and *Vps4*^*DN*^ mutant neurons, in contrast to its discrete distribution in wild type. Moreover, both *prd1* and *α-ada* are important for endo-lysosomal degradation of Nrg. Finally, they genetically interacted during dendrite pruning. Reduction of *prd1* gene dose (*prd1*^*M56*^*/+*) dominantly enhanced the pruning phenotypes of *α-ada* RNAi mutants. The functional link between Prd1 and α-Ada suggests that Prd1 and α-Ada act together to regulate endo-lysosomal degradation. Similar to Prd1, in a previous elegant study, Numb-associated kinase (Nak), a *Drosophila* Actin-related kinase (Ark) family member, was identified as another binding partner of the clathrin adaptor protein AP-2 [20]. Nak distributes clathrin puncta in higher-order dendrites to promote dendritic growth [20]. However, we did not observe prominent dendrite pruning defects using a null *nak*^*2*^ mutant (*n* = 29), suggesting that AP-2-associated Nak is dispensable for ddaC dendrite pruning. Thus, it is conceivable that Prd1 acts as a novel binding protein of AP-2 to facilitate endo-lysosomal degradation of Nrg in the dendrites and promote dendrite pruning in sensory neurons.

### Imac regulates α-Ada/clathrin distribution and endo-lysosomal degradation of the L1-CAM Nrg

Kinesin motor proteins transport intracellular cargos along microtubule tracks [39]. Imac/Unc-104 belongs to the evolutionarily conserved kinesin-3 family. Imac/Unc-104 was reported to regulate synapse formation and synaptic vesicle transport in axons in fly and worm [33,40–43]. In mammals, two Imac homologues, namely kinesin (KIF)1A and KIF1Bβ, transport synaptic vesicle precursors in axons [44,45]. Here, we provided multiple lines of genetic evidence using RNAi knockdown, loss-of-function mutants, and dominant negative approaches, unambiguously demonstrating that Imac is required for dendrite pruning, consistent with a recent report on *imac* RNAi knockdown phenotypes in ddaC neurons [46]. More importantly, for the first time we provide mechanistic insight into how the *Drosophila* kinesin-3 Imac regulates the distribution of α-Ada/clathrin puncta and promotes dendrite pruning of sensory neurons. *imac* mutant ddaC neurons showed severe defects in dendrite pruning and initial dendrite arborization, resembling *α-ada* mutants. *imac* displayed significant genetic interaction with *α-ada*. Moreover, Imac appeared to colocalize with the clathrin adaptor α-Ada and its interacting protein Prd1 in the dendrites. Interestingly, in *imac* mutant neurons, clathrin, α-Ada, and Rab5/Hrs accumulated into numerous aberrant aggregates, suggesting aberrant endosomal formation/distribution. The aberrant aggregates were also rich in ubiquitin but lacked LysoTracker signals, indicative of defective endo-lysosomal degradation. Furthermore, the L1-CAM Nrg protein accumulated dramatically in the somas, dendrites, and axons of *Imac*^*G102E*^ ddaC neurons (this study), whereas Nrg was drastically degraded via endo-lysosomal degradation pathway in wild-type neurons [23]. Therefore, our study demonstrates a novel role of Imac in promoting endo-lysosomal maturation/degradation in sensory neurons. Similarly, *Caenorhabditis elegans* Unc-104 regulates autophagosome formation and maturation in neurons [47]. Therefore, it is possible that the primary role of *imac* is to facilitate the formation or fusion of early endosomes and thereby lysosome-mediated degradation of the L1-CAM Nrg during dendrite pruning.

### Prd1 recruits α-Ada-positive puncta to the kinesin-3 Imac in sensory neurons

Our cell biological, biochemical, and genetic data reveal a functional link between Prd1 and the kinesin-3 Imac in dendrite pruning. First, we show that Prd1 colocalizes with Imac but not with kinesin-1/2 or dynein. Second, Imac is required for the normal distribution of Prd1 and endocytic components α-Ada/clathrin, presumably endocytic vesicles, in dendrites. Third, Prd1 forms a protein complex with α-Ada and Imac but not with Rab5 or kinesin-1. Finally, *prd1* and *imac* are required for dendrite pruning in the same genetic pathway.

How does *imac* regulate endo-lysosomal degradation of Nrg to promote dendrite pruning? Our previous findings demonstrated that Rab5-dependent endocytosis regulates dendrite pruning via endo-lysosomal degradation of the L1-CAM Nrg [23]. We show here that Imac colocalized with the clathrin adaptor α-Ada via Prd1 in the dendrites but not with Rab5. Moreover, co-IP experiments suggested that Prd1 formed a protein complex with α-Ada but not with Rab5. One possibility is that the Prd1/α-Ada/Imac pathway might function at the internalization step of endocytosis, preceding the function of Rab5, to facilitate the formation/fusion of early endosomes and thereby endo-lysosomal maturation/degradation during dendrite pruning. We envision that upon the cleavage of clathrin-coated vesicles, Prd1 associates with the clathrin adaptor protein AP-2 to recruit the newly formed vesicles to the kinesin-3 Imac, which in turn delivers them for the formation and fusion of early endosomes; early endosomes undergo endo-lysosome maturation and degradation, leading to dendrite pruning. However, in the established model of CME, AP-2/clathrin-coated endocytic vesicles are uncoated after budding; clathrin and AP-2 are released back into the cytoplasm to participate in another round of CME. The possible speculation is that Imac might deliver the α-Ada/clathrin-coated vesicles before their uncoating. Alternatively, α-Ada/AP-2 might play a noncanonical role in regulating endo-lysosomal degradation after the formation of early endosomes. Growing studies have shown noncanonical functions of the adaptor proteins AP-1/AP-2 and clathrin in vitro. In cultured cells, clathrin or AP-1/AP-2-associated vesicles can be directly distributed by kinesin or dynein motors [48,49]. Moreover, noncanonical function of AP-2 has been also reported in a recent study, in which AP-2 acts as an adaptor that links autophagosomes to a dynein motor for proper vesicular distribution in cultured neurons [50]. Further investigations are required to distinguish these two possible mechanisms whereby the Prd1/α-Ada/Imac pathway regulates endo-lysosomal degradation of Nrg to promote dendrite pruning.

In summary, we identified a novel protein Prd1 and two associated proteins, including the clathrin adaptor α-Ada and the kinesin-3 Imac, which all play crucial roles in regulating dendrite pruning of sensory neurons. Mechanistically, Prd1, α-Ada, and Imac act in the same pathway to regulate discrete distribution of α-Ada/clathrin puncta, facilitate endo-lysosomal degradation of the L1-CAM Nrg, and thereby promote dendrite pruning in sensory neurons.

## Materials and methods

### Fly strains

Fly strains used in this study include *α-ada*^*3*^ (H. Jackle) [29], *AP-2μ*^*NN20*^ (D. Bilder) [30], *Rab5*^*2*^, *UAS-Rab5*^*DN*^, *UAS-GFP*-*Rab5* (M. Gonzalez-Gaitan) [51], *imac*^*170*^, *imac*^*172*^, *UAS-Imac-RFP* (T.L. Schwarz) [33], *UAS-Vps4*^*DN*^ (H. Stenmark) [52], *UAS-GFP-α-Ada* (B. Lu) [53], *UAS-GFP-Clc*, *UAS-mRFP-Chc*, *Cul1*^*Ex*^ (C.T. Chien) [20,54], *mical*^*15256*^ (Yu lab) [13], *SOP-flp (#42)*, *UAS-Dlic*::*EGFP* (T. Uemura) [55], *ppk-Gal4* on II and III chromosome (Y. Jan) [56], *UAS-mRFP-Lis1* (A. Moore) [57], *UAS-Kap3-RFP* (K. Ray) [58], *UAS-Khc-GFP* (Yu lab), *UAS-Imac*^*G102E*^, *UAS-Imac*^*AAA*^, *UAS-Venus-Prd1*, *UAS-Prd1*, *UAS-Bap* (this study), *UAS-Sec15-GFP* (H. Bellen) [59], *AP-1μ*^*SHE-11*^ (R.L. Borgne) [31], and *UAS-Arf79F-EGFP* (T.J. Harris) [60].

The following stocks were obtained from Bloomington Stock Center (BSC): *Gal4*^*109(2)80*^, *elav-Gal4*, *ppk-CD4-tdGFP* (BL#35843), *GSG2295-Gal4* (BL#40266), *CG17360M56* (BL#37744), *Df(3R)PS1* (BL#37741), *Df(3R)PS2* (BL#37742), *Df(3R)Exel7310* (BL#7965), *α-ada* RNAi #2 (BL#32866), *khc* RNAi (BL#25898), *P{EPgy2}EY01200* (BL#15065), *Dp(1;Y)BSC140* (BL#30461), *UAS-GalT-GFP* (BL#30902), *UAS-mito-HA-GFP* (BL#8442), *UAS-PLC-δ-PH-GFP* (BL#39693), *AP-1γ*^*B*^ (BL#57051), *AP-1γ*^*D*^ (BL#57052), *nrg* RNAi (BL#38215), *UAS-GCaMP3* (BL#32116), and *UAS-GCaMP6* (BL#42746).

The following stocks were obtained from Vienna *Drosophila* RNAi Centre (VDRC): *prd1* RNAi #1 (v108557), *prd1* RNAi #2 (v40070), *α-ada* RNAi #1 (v15566), *imac* RNAi #1 (v47171), *imac* RNAi #2 (v23465), and control RNAi (v36355).

### Generation of *prd1*, *Bap*, and *imac* transgenes

*prd1* and *Bap* full-length cDNAs were PCR from EST LP07755 and LP17054 (DGRC) into Topo Entry vector (Life Tech, Carlsbad, CA). The GATEWAY *pTW* or *pTVW* vectors containing the respective fragment of the cDNAs were constructed by LR reaction (Life Tech, Carlsbad, CA).

The variants of Imac were generated by G102E and GKS102-104AAA site mutagenesis (Agilent Tech) using *pUAST-imac-HA* as a template, respectively. The respective cDNA fragments were amplified by PCR and subcloned into *pTWH* vector (DGRC). The transgenic lines were established by the Bestgene.

### Generation of *Bap* mutant

*P{EPgy2}EY01200* P-element insertion flies were crossed with the fly strain carrying the Δ2–3 transposase to induce imprecise excision events. Nearly 600 independent lines were established based on a loss of the *w+* marker. One of lethal lines was found to be rescued by the duplication line *Dp(1;Y)BSC140*. Subsequent genomic PCR and DNA sequencing analysis indicated that this mutant *Bap*^*Δ1*^ harbors a 1,381-bp deletion.

### Generation of Prd1 antibody

cDNA fragments corresponding to aa151–350 (antigen 1), aa300–575 (antigen 2), and aa 591–724 (antigen 3) of *prd1* isoform A were amplified from EST LP07755 by Expand High Fidelity PCR System (Roche) and verified by DNA sequencing. The resultant products were expressed by the GST expression vector (*pGEX4T-1*, Pharmacia). After protein purification, the purified protein was used to immunize various guinea pigs and rats to generate polyclonal antibodies against Prd1. The specificity of the antibody was verified in ddaC neurons expressing both *prd1* RNAi and *imac* RNAi lines.

### Live imaging analysis

To image *Drosophila* da neurons at the wandering third instar (wL3) or WP stage, larvae or pupae were first washed in PBS buffer briefly, followed by immersion with 90% glycerol. For imaging da neurons at 16 h APF, pupal cases were carefully removed before they were mounted with 90% glycerol. Dendrite images were acquired on Leica TSC SP2. Subcellular localization images were acquired on Leica TCS SP8 STED 3× super-resolution microscope.

### MARCM analysis of da sensory neurons

MARCM analysis, dendrite imaging, and quantification were carried out as previously described [13]. ddaC or other da clones were selected and imaged at the WP stage according to their location and morphology. The ddaC or other da neurons were examined for dendrite pruning defects at 16 h or 20 h APF.

### RU486/mifepristone treatment for the Gene-Switch system

Embryos of appropriate genotype were collected at 6-h intervals and reared on standard food to the early third instar larva stage. The larvae were transferred to the standard culture medium, which contains 240 μg/mL mifepristone (Sigma Aldrich M8046). Neither puparium formation onset nor adult eclosion was affected by RU486 treatment. White prepupae with appropriate genotype were picked up, subject to dissection and phenotypic analysis at 16 h APF.

### Immunohistochemistry and antibodies

The following primary and secondary antibodies were used for immuno-histochemistry at the indicated dilution: rat anti-Prd1 (1:200) (this study), rabbit anti-α-Ada (1:200), guinea pig anti-Avl (1:500; Yu lab), guinea pig anti-Hrs (1:300) [61], mouse anti-Ubiquitin (1:500; FK2, Enzo Life Sciences), rabbit anti-GFP (1:500, Invitrogen), mouse anti-GPF (1:500, Yu lab), guinea pig anti-Sec15 (1:200), rabbit anti-GM130 (1:200, Abcam), mouse anti-KDEL (1:200; 10C3, Abcam), mouse anti-Nrg (1:20, BP104, DSHB). Cy5-conjugated goat anti-HRP was used at 1:200 dilutions, Cy3 or fluorescein isothiocyanate (FITC) conjugated secondary antibodies were used at 1:500 dilutions, and Cy5 conjugated secondary antibodies were used at 1:200 dilutions. For immune-staining assays, wild-type and mutant pupae or larvae were dissected in cold PBS and fixed in 4% formaldehyde for 12 min. The samples within the same group of experiments were stained in the same tube and mounted in VectaShield mounting medium, and the samples were directly visualized by Leica TCS SP8 STED 3 × super-resolution microscope and processed in parallel. Data analysis and statistics were performed via Excel (Microsoft), and Imaris software. Based on the intensity profiles, the localization patterns were divided into three categories: colocalization, adjacent localization, and non-colocalization, as shown in the representative images (S25 Fig).

### Quantification of ddaC dendrites

Live confocal images of da neurons expressing *mCD8-GFP* under the control of *ppk-Gal4* or *elav-Gal4* were shown at WP, 16 h APF and 20 h APF. For wild-type or mutant ddaC neurons, the percentages of fragmentation defect and severing defect were quantified in a 275 μm × 275 μm region of the dorsal dendritic field, originating from the abdominal segments 2–5. The severing defect was defined by the presence of dendrites that remain attached to the soma at 16 h APF, whereas dendrite fragmentation defect is referred to as the presence of dendrite branches near the ddaC territory that have been severed from their proximal parts at 16 h APF [11–13]. Total length of unpruned dendrites was measured in a 275 μm × 275 μm region of the dorsal dendritic field using ImageJ. The number of samples (*n*) in each group is shown on the bars. Statistical significance was determined using either two-tailed Student *t* test (two samples) or one-way ANOVA and Bonferroni test (multiple samples) (*p < 0.05, **p < 0.01, ***p < 0.001, n.s., not significant). Error bars represent SEM. Dorsal is up in all images.

### Co-IP assay

S2 cell culture and western blotting were carried out as described below. Myc-Prd1, Flag-α-Ada, Flag-Rab5, Khc-Flag, and Imac-HA expression vectors were generated by Gateway cloning. S2 cells were cultured at 25°C in Express Five SFM (Gibco) medium supplemented with 1% L-glutamine. For transfection, S2 cells were plated at a density of 2 × 10^6^ cells per 35-mm dish 1 d before transfection. After 24 h culture, around 0.1–0.5 μg of each expression plasmid was transfected into the S2 cells using Effectene Transfection Reagent (Qiagen); cells were harvested 48 h post-transfection. Transfected S2 cells were homogenized with lysis buffer (25 mM Tris pH 8/27.5 mM NaCl/20 mM KCl/25 mM sucrose/10 mM EDTA/10 mM EGTA/1 mM DTT/10% [v/v] glycerol/0.5% Nonidet P40) with protease inhibitors (Complete, Boehringer; PMSF 10 mg/mL, sodium orthovanadate 10 mg/mL). The supernatants were used for IP with anti-Myc, anti-Flag, or anti-HA overnight at 4°C followed by incubation with protein A/G beads (Pierce Chemical Co.) for 2 h. Protein A/ G beads were washed four times using cold PBS. Bound proteins were separated by SDS-PAGE and analyzed by western blotting with anti-Myc, anti-Flag, and anti-HA HRP-conjugated antibody. Co-IP experiments were repeated three times.

### Calcium imaging

Calcium imaging was performed with Olympus FV3000 using 60× Oil lens. Calcium images from 6.5 h APF ddaC neurons were acquired for 400–500 frames at 1 frame per 1.8–2.25 s and analyzed using Metamorph (Molecular Devices) and ImageJ software.

## Supporting information

S1 Fig *Drosophila* Prd1 shares sequence similarity with mammalian SKIP and its mutant shows reduced dendrite termini. (A) Protein structures of *Drosophila* Prd1 and mammalian SKIP. The C-terminal portions of Prd1 and SKIP contain a PH domain and share sequence identity (27% and 24%). (B) A schematic diagram of *prd1* gene locus and mutants. Those *prd1* mutants are derived from three P-element insertion lines, namely *PBac{RB}prd1*^*e02295*^, *PBac{WH}prd1*^*f05458*^, and *P{XP}I(3)neo38*^*d03325*^. (C) Live confocal images of *FRT82B* control and *prd1*^*M56*^ ddaC MARCM clones at wL3 stage as well as quantification of number of dendrite termini in control and *prd1*^*M56*^ ddaC MARCM clones. (D) Dendrites of control and *prd1*^*PS1*^ MARCM ddaC neurons showing fragmentation defect at 16 h APF. Red arrowheads point to the ddaC somas. Open red arrowhead points to the severed dendrite branches. *p < 0.05 as assessed by two-tailed Student *t* test. Error bars represent SEM. Scale bars represent 50 μm. The individual numerical values for panel C can be found in S1 Data. The genotypes can be found in S1 Text. APF, after puparium formation; MARCM, mosaic analysis with a repressible cell marker; PH, pleckstrin homology; Prd1, pruning defect 1; SKIP, SifA and Kinesin-interacting protein; wL3, wandering third instar.
(TIF)

S2 Fig Prd1 is required for ddaD/E dendrite pruning but not for ddaF apoptosis. (A) Live confocal images of ddaD/E MARCM clones labeled by *UAS-mCD8-GFP* at WP and 20 h APF. ddaD/E somas are marked by blue arrowheads. Control ddaD/E MARCM clones pruned all their dendrites at 20 h APF, whereas larval dendrites were still attached to the somas of *prd1*^*M56*^ ddaD/E MARCM clones. (B) Live confocal images of ddaF MARCM clones at WP and 16 h APF. ddaF somas are marked by green arrowheads. *prd1*^*M56*^ ddaF MARCM clones underwent apoptosis, similar to FRT82B control clones. Scale bar (A) represents 50 μm. The genotypes can be found in S1 Text. APF, after puparium formation; MARCM, mosaic analysis with a repressible cell marker; *prd1*, *pruning defect 1*; WP, white prepupal.
(TIF)

S3 Fig Venus-Prd1 expression is detected by the anti-Prd1 antibody in soma, dendrites, and axons of ddaC neurons. (A) A schematic diagram of Prd1 protein with three antigens indicated in red lines. Antigens 1–3 contain aa151–350, aa300–575, and aa591–724, respectively. (B) Distribution of Venus-Prd1 and anti-Prd1 staining in ddaC neurons expressing Venus-Prd1. Venus-Prd1 colocalized with anti-Prd1 signals in soma, dendrites (arrowheads), and axon (arrowhead). (C) Distribution of Venus-Prd1 and Arf79F-EGFP in ddaC neurons. Venus-Prd1 puncta localized distinctly from Arf79F-EGFP (insets) in dendrites of ddaC neurons. Dorsal is up in all images. Scale bars in (B) and (C) represent 10 μm. The genotypes can be found in S1 Text. Arf79F-EGFP, ADP ribosylation factor 79F fused with enhanced green fluorescent protein; Prd1, Pruning defect 1.
(TIF)

S4 Fig Prd1 is enriched on enlarged endosomes in *Rab5^DN^* or *Vps4^DN^* ddaC neurons. (A–B) Confocal images of control or mutant ddaC neurons at wL3 stage. ddaC somas are marked by dashed lines. Compared with control, Venus-Prd1 enriched on aberrant endosomes in *Rab5*^*DN*^ (A) and *Vps4*^*DN*^ (B) ddaC neurons. Similarly, GFP-α-Ada and GFP-Clc also accumulated on aberrant ubiquitin-positive endosomes in *Rab5*^*DN*^ (A) and *Vps4*^*DN*^ (B) ddaC neurons. Scale bar (A) represents 10 μm. The genotypes can be found in S1 Text. GFP-α-Ada, green fluorescent protein fused with α-Adaptin; GFP-Clc, green fluorescent protein fused with Clathrin light chain; Prd1, Pruning defect 1; *Rab5*^*DN*^, Rabaptin-5 dominant-negative form; *Vps4*^*DN*^, Vacuolar protein sorting-associated protein 4 dominant negative form; wL3, wandering third instar.
(TIF)

S5 Fig Loss of *Rab5* or *ESCRT* function does not affect Mito-GFP, GM130, and KDEL distributions. Confocal images of control or mutant ddaC neurons at wL3 stage. Distributions of Mito-GFP, GM130 and KDEL were similar to control ddaC neurons and absent from the aberrant endosomes in *Rab5*^*DN*^ or *Vps4*^*DN*^ ddaC neurons. Quantifications with Pearson’s correlation coefficients indicate no colocalization between endosomes and these cellular markers. n.s., not significant, as assessed by one-way ANOVA test. ddaC somas are marked by dashed lines. Scale bar represents 10 μm. The individual numerical values for panels can be found in S1 Data. The genotypes can be found in S1 Text. *ESCRT*, *endosomal sorting complexes required for transport*; Mito-GFP, mitochondria-GFP; *Rab5*, *Rabaptin-5*; wL3, wandering third instar.
(TIF)

S6 Fig Prd1 forms a complex with Bap in S2 cells. (A–B) Co-IP between Prd1 and Bap. Prd1 and Bap associated with each other in S2 cells co-transfected with Myc-Prd1 and Flag-Bap in co-IP experiments. Bap, β-Adaptin; co-IP, co-immunoprecipitation; Myc-Prd1, Myc-tagged Prd1; Prd1, pruning defect 1.
(TIF)

S7 Fig Prd1 does not interact with Rab5 in S2 cells. (A–B) Co-IP between Prd1 and Rab5. No interaction between Prd1 and Rab5 was observed in S2 cells transfected with Myc-Prd1 and Flag-Rab5. co-IP, co-immunoprecipitation; Myc-Prd1, Myc-tagged Prd1; Prd1, pruning defect 1; Rab5, Rabaptin-5.
(TIF)

S8 Fig Knockdown of *α-ada* results in dendrite pruning defects. Live confocal images of ddaC neurons expressing mCD8-GFP at WP and 16 h APF. Red arrowheads indicate ddaC somas. All dendrites of control ddaC neurons are pruned at 16 h APF; however, dendrites failed to be pruned in *α-ada* RNAi ddaC neurons. The number of samples (*n*) in each group is shown on the bars. Scale bar represents 50 μm. Dorsal is up in all images. The individual numerical values for panels can be found in S1 Data. The genotypes can be found in S1 Text. *α-ada*, *α*-*adaptin*; APF, after puparium formation; mCD8-GFP, membrane-associated green fluorescent protein; RNAi, RNA interference; WP, white prepupal.
(TIF)

S9 Fig AP-2 complex is required for ddaD/E dendrite pruning but not for ddaF apoptosis. (A) Live confocal images of ddaD/E neurons at WP and 20 h APF. ddaD/E somas are marked by blue arrowheads. Control ddaD/E neurons pruned all dendrites, while some larval dendrites of *α-ada*^*3*^ ddaD/E clones remained attached to the somas at 20 h APF. (B) ddaF MARCM clones at WP and 16 h APF. ddaF somas are marked by green arrowheads. Similar to the control, *α-ada*^*3*^ ddaF MARCM clones undergo apoptosis during early metamorphosis. (C) A schematic diagram of the *Bap* gene and the deleted region of the *Bap*^*Δ1*^ mutant. *Bap*^*Δ1*^ was generated by the P-element insertion *P{EPgy2}EY01200*. (D) Expression of *α*-Ada protein in sensory neurons. ddaC and ddaF somas are marked by dashed lines. Scale bars (A) and (D) represent 50 μm and 10 μm, respectively. Dorsal is up in all images. The genotypes can be found in S1 Text. *α*-Ada, α-Adaptin; APF, after puparium formation; MARCM, mosaic analysis with a repressible cell marker; WP, white prepupal.
(TIF)

S10 Fig AP-1μ and AP-1*γ* are dispensable for dendrite pruning of ddaC neurons. Live confocal images of control, *AP-1*μ^*SHE-11*^, *AP-1γ*^*B*^, and *AP-1γ*^*D*^ ddaC neurons at WP and 16 h APF. ddaC somas are marked by red arrowheads. Quantification analysis of percentage of severing defect and fragmentation defect in control and mutant ddaC neurons at 16 h APF. The number of samples (*n*) in each group is shown on the bars. Scale bar represents 50 μm. The individual numerical values for panel can be found in S1 Data. The genotypes can be found in S1 Text. AP-1μ, Adaptor protein-1 μ subunit; AP-1γ, Adaptor protein-1 γ subunit; APF, after puparium formation; WP, white prepupal.
(TIF)

S11 Fig Distribution of Prd1 and kinesins/dyneins. (A–D) Distribution of Prd1 and kinesin/dynein motors. Khc-GFP (A), Kap3-RFP (B), Dlic-EGFP (C), or mRFP-Lis1 (D) showed distinct pattern with Venus-Prd1 in ddaC neurons. ddaC somas are marked by dashed lines. Scale bar (A) represents 10 μm. Dorsal is up in all images. The genotypes can be found in S1 Text. Dlic-EGFP, Dlic fused with enhanced green fluorescent protein; Kap3-RFP, Kinesin associated protein 3 fused with red fluorescent protein; Khc-GFP, Kinesin heavy chain fused with green fluorescent protein; mRFP-Lis1, monomeric red fluorescent protein fused with Lis1; Prd1, Pruning defect 1.
(TIF)

S12 Fig Prd1 colocalizes with α-Ada and Imac in the axons of ddaC neurons. (A) Distribution of GFP-α-Ada and Venus-Prd1 in ddaC axons. Venus-Prd1 colocalized with GFP-α-Ada in the axons (arrowhead). (B) Distribution of Venus-Prd1 and Imac-RFP in ddaC axons. Venus-Prd1 colocalized with Imac-RFP in the axons (arrowhead). (C) Distribution of GFP-α-Ada and Imac-RFP in ddaC axons. GFP-α-Ada puncta did not overlap with Imac-RFP in the axons. (D) Distribution of Venus-Prd1, GFP-α-Ada, and Imac-RFP in ddaC axons. Venus-Prd1, GFP-α-Ada, and Imac-RFP colocalized in ddaC axons (arrowhead). Scale bars represent 10 μm. The genotypes can be found in S1 Text. α-Ada, α-Adaptin; GFP, green fluorescent protein; Imac-RFP, immaculate connections fused with red fluorescent protein; Prd1, Pruning defect 1.
(TIF)

S13 Fig Schematic representation of two *Imac^DN^* variants as well as their effects on dendrite pruning and morphology. Imac contains a motor domain (blue), coiled coil domains 1–3 (yellow), FHA domain (pink), and a PH domain (green). FHA, forkhead-associated; *imac*, *immaculate connections*; PH, pleckstrin homology.
(TIF)

S14 Fig The expressions of *Imac^G102E^* or *Imac^AAA^* cause dendrite pruning defects. Live confocal images of ddaC neurons labeled by *mCD8-GFP* at WP and 16 h APF. ddaC somas are indicated by red arrowheads. Dendrites of *imac* RNAi #2, *Imac*^*G102E*^, or *Imac*^*AAA*^ mutant ddaC neurons remained attached to their somas at 16 h APF. Quantification of severing defect and fragmentation defect in *imac* mutant ddaC neurons at 16 h APF. The number of samples (*n*) in each group is shown on the bars. Scale bar represents 50 μm. Dorsal is up in all images. The individual numerical values for panels can be found in S1 Data. The genotypes can be found in S1 Text. APF, after puparium formation; *imac*, *immaculate connections*; RNAi, RNA interference; WP, white prepupal.
(TIF)

S15 Fig Inducible expression of *Imac^AAA^* causes dendrite pruning defects. Using the Gene-Switch system, inducible expression of *Imac*^*AAA*^ at the eL3 stage caused severe dendrite pruning defects at 16 h APF, whereas inducible expression of the control *UAS* transgene showed normal dendrite pruning. Quantification of severing defect and fragmentation defect in mutant ddaC neurons at 16 h APF. The number of samples (*n*) in each group is shown on the bars. Scale bar represents 50 μm. Dorsal is up in all images. The individual numerical values for panels can be found in S1 Data. The genotypes can be found in S1 Text. APF, after puparium formation; eL3, early third instar.
(TIF)

S16 Fig Venus-Prd1 and GFP-α-Ada aggregate in *imac* RNAi ddaC neurons. Distribution of Venus-Prd1 (in green) and GFP-α-Ada (in green) in control and *imac* RNAi (#2) ddaC neurons. ddaC somas are marked by dashed lines. Scale bar represents 10 μm. Dorsal is up in all images. The genotypes can be found in S1 Text. GFP-α-Ada, green fluorescent protein fused with α-Adaptin; *imac*, *immaculate connections*; RNAi, RNA interference; Venus-Prd1, Venus-Pruning defect 1.
(TIF)

S17 Fig Knockdown of *khc* function does not affect the distribution of Venus-Prd1 and endogenous α-Ada. Distribution of Venus-Prd1 (in green) and α-Ada (in red) in control and *khc* RNAi ddaC neurons. ddaC somas are marked by dashed lines. Scale bar represents 10 μm. Dorsal is up in all images. The genotypes can be found in S1 Text. α-Ada, α-Adaptin; *khc*, *kinesin heavy chain*; Prd1, Pruning defect 1; RNAi, RNA interference.
(TIF)

S18 Fig Imac regulates the distribution of the endosomal markers Rab5 and Hrs but not the secretory marker Sec15 nor the Golgi marker GalT-GFP. (A) Distribution of the endosomal marker Hrs (in red) in control and *imac* RNAi (#1) ddaC neurons overexpressing Venus-Prd1 (in green). (B) Distribution of the early endosomal marker GFP-Rab5 (in red) in control and *imac* RNAi (#1) ddaC neurons overexpressing Venus-Prd1 (in green). (C) Distribution of the secretory marker Sec15 (in red) in control and *imac* RNAi (#1) ddaC neurons overexpressing Venus-Prd1 (in green). (D) The table shows the colocalization ratios (Pearson’s correlation coefficients) of Venus-Prd1 with different vesicular markers in *imac* RNAi neurons. *n* represents the number of neurons examined in each group. (E) Distribution of GalT-GFP (in green) in control and *imac* RNAi (#1) ddaC neurons. ddaC somas are marked by dashed lines. Scale bar (A) represents 10 μm. Dorsal is up in all images. The individual numerical values for panels D and E can be found in S1 Data. The genotypes can be found in S1 Text. GalT-GFP, GalT fused with GFP; Hrs, hepatocyte growth factor-regulated tyrosine kinase substrate; Rab5, Rabaptin 5; RNAi, RNA interference.
(TIF)

S19 Fig Nrg protein accumulated dramatically in the somas, dendrites, and axons of *prd1*, *α-ada*, and *imac* mutant ddaC neurons prior to dendrite pruning. (A–D) The distribution of Nrg in control (A), *prd1*^*M56*^/*Df (3R)Exel7310* (B), ***α****-ada*^*3*^ MARCM (C), or *imac*^*G102E*^ (induced) (D) ddaC neurons at 6 h APF. ddaC somas are marked by dashed lines, axons by arrows, and proximal dendrites by curly brackets. ddaE somas are marked by asterisks. (E) Quantification of Nrg immunostaining intensity. The number of samples (*n*) in each group is shown on the bars. Error bars represent SEM. ***p < 0.001 as assessed by one-way ANOVA test. Dorsal is up in all images. The individual numerical values for panel E can be found in S1 Data. The genotypes can be found in S1 Text. *α-ada*, *α-adaptin*; APF, after puparium formation; *imac*, *immaculate connections*; MARCM, mosaic analysis with a repressible cell marker; Nrg, Neuroglian; *prd1*, *pruning defect 1*.
(TIF)

S20 Fig *nrg* knockdown suppressed dendrite pruning defects of *prd1*, *α-ada*, and *imac* mutant ddaC neurons. (A–C) Live confocal images of ddaC neurons expressing *UAS-mCD8-GFP* driven by *ppk-Gal4* at 16 h APF. *nrg* RNAi knockdown significantly rescued pruning defects of *prd1*^*M56*^/*prd1*^*PS2*^ (A), ***α****-ada*^*3*^ MARCM (B), or *imac* RNAi (C) ddaC neurons at 16 h APF. ddaC somas are marked by red arrowheads. Quantification analysis of percentage of severing defect and fragmentation defect in control and mutant ddaC neurons at 16 h APF. Quantification of total length of unpruned ddaC dendrites at 16 h APF. The number of samples (*n*) in each group is shown on the bars. Error bars represent SEM. ***p < 0.001 as assessed by one-way ANOVA test. Scale bar represents 50 μm. The individual numerical values for panels A, B, and C can be found in S1 Data. The genotypes can be found in S1 Text. *α-ada*, *α-adaptin*; APF, after puparium formation; *imac*, *immaculate connections*; MARCM, mosaic analysis with a repressible cell marker; *nrg*, *neuroglian*; *prd1*, *pruning defect 1*; RNAi, RNA interference.
(TIF)

S21 Fig Compartmentalized Ca^2+^ transients are impaired in *prd1* and *imac* mutant ddaC neurons. The percentage of neurons with dendritic calcium transients was reduced at 6.5 h APF in *prd1*^*M56*^/*Df(3R)Exel7310* and *Imac*^*G102E*^ mutant neurons, compared to the control neurons. The number of samples (*n*) in each group is shown on the bars. The individual numerical values for panels can be found in S1 Data. The genotypes can be found in S1 Text. APF, after puparium formation; *imac*, *immaculate connections*; *prd1*, *pruning defect 1*.
(TIF)

S22 Fig Prd1 does not interact with Khc, and Imac does not associate with Rab5 in S2 cells. (A–B) Co-IP between Prd1 and Khc. Prd1 did not interact with Khc when S2 cell extracts were immunoprecipitated with anti-Flag (A) or anti-Myc (B) antibody. (C–D) Co-IP between Imac and Rab5. In S2 cell extracts co-transfected with Imac-HA and Flag-Rab5, no interaction was detected when immunoprecipitated with anti-HA (C) or anti-Flag (D) antibody. co-IP, co-immunoprecipitation; HA, HA tag; Imac, Immaculate connections; Khc, Kinesin heavy chain; Myc, Myc tag; Prd1, Pruning defect 1; Rab5, Rabaptin 5.
(TIF)

S23 Fig *prd1* does not genetically interact with *mical* or *cul1*. (A) Dendrites of *mical*^*15256*^*/+*, *prd1*^*M56*^*/ Df(3R)Exel7310*, and *mical*^*15256*^, *prd1*^*M56*^*/ Df(3R)Exel7310* ddaC neurons at 16 h APF. (B) Dendrites of *cul1*^*Ex*^*/+*, *prd1*^*M56*^*/Df(3R)Exel7310*, and *cul1*^*Ex*^*/+; prd1*^*M56*^*/Df(3R)Exel7310* ddaC neurons at 16 h APF. Quantification of total length of unpruned dendrites at 16 h APF. The number of samples (*n*) in each group is shown on the bars. Error bars represent SEM. Scale bar (A) represents 50 μm. Dorsal is up in all images. The individual numerical values for panels A and B can be found in S1 Data. The genotypes can be found in S1 Text. APF, after puparium formation; *cul1*, *cullin1*; *mical*, *molecule interacting with CasL*; n.s., not significant; *prd1*, *pruning defect 1*.
(TIF)

S24 Fig A working model. We propose that Prd1, α-Ada, and Imac act in the same pathway to promote dendrite pruning via endo-lysosomal degradation of the L1-CAM Nrg. Down-regulation of Nrg is required to trigger dendrite pruning in ddaC neurons during early metamorphosis. α-Ada, α-Adaptin; Imac, Immaculate connections; L1-CAM, L1-type cell adhesion molecule; Nrg, Neuroglian; Prd1, Pruning defect 1.
(TIF)

S25 Fig Subcellular localization analyses. Based on the intensity profiles, the localization patterns were divided into three categories: (A) colocalization, (B) adjacent localization, (C) non-colocalization. The genotypes can be found in S1 Text.
(TIF)

S1 Text The genotypes of fly strains used in main figs and supporting figs. (DOCX)

S1 Data Data for Figs 1, 2, 4, 5, 6 and 8 and S1, S5, S8, S10, S14, S15, S18, S19, S20, S21, S23 and S25 Figs. (XLSX)

## Abbreviations

α-Ada: α-Adaptin
α-ada^3^: α-adaptin^3^
AEL: after egg laying
AP-1: Adaptor protein-1
AP-2: Adaptor protein-2
AP-2α: the α subunit of the AP-2 complex
APF: after puparium formation
Arf79F-EGFP: ADP ribosylation factor 79F fused with enhanced green fluorescent protein
Ark: Actin-related kinase
Arl8: Arf-like GTPase 8
Bap: β-Adaptin
BSC: Bloomington Stock Center
Chc: Clathrin heavy chain
Clc: Clathrin light chain
CME: Clathrin-mediated endocytosis
co-IP: co-immunoprecipitation
da: dendritic arborization
dda: dorsal dendrite arborization
Dlic: Dynein light intermediate chain
ecdysone: 20-hydroxyecdysone
eL3: early third instar
ER: endoplasmic reticulum
ESCRT: endosomal sorting complexes required for transport
FITC: fluorescein isothiocyanate
FLP: flippase
FRT: flippase recognition target
GalT: β1,4-galactosyltransferase
GFP: green fluorescent protein
Hrs: hepatocyte growth factor-regulated tyrosine kinase substrate
imac: immaculate connections
Kap3: Kinesin associated protein 3
Khc: Kinesin heavy chain
L1-CAM: L1-type cell adhesion molecule
MARCM: mosaic analysis with a repressible cell marker
MB: mushroom body
mRFP: monomeric red fluorescent protein
Nak: Numb-associated kinase
Nrg: Neuroglian
O/E: overexpression
PH: pleckstrin homology
PLC-δ-PH: phospholipase C-δ-pleckstrin homology
ppk-Gal4: pickpocket-Gal4
prd1: pruning defect 1
PtdIns(4,5)P2: phosphatidylinositol 4,5-bisphosphate
Rab5: Rabaptin-5
RFP: red fluorescent protein
RNAi: RNA interference
SifA: Salmonella induced filament A
SKIP: SifA and Kinesin-interacting protein
UAS: upstream activating sequence
UNC-104: Uncoordinated-104
VDRC: Vienna Drosophila RNAi Centre
Vps4: Vacuolar protein sorting-associated protein 4
wL3: wandering third instar
WP: white prepupal
