# Supplementary figures and images for: Prd1 associates with the clathrin adaptor α-Adaptin and the kinesin-3 Imac/Unc-104 to govern dendrite pruning in Drosophila
Source: PLoS Biol. 2018 Aug 24;16(8):e2004506. doi: 10.1371/journal.pbio.2004506 (PMC6126864; doi:10.1371/journal.pbio.2004506)

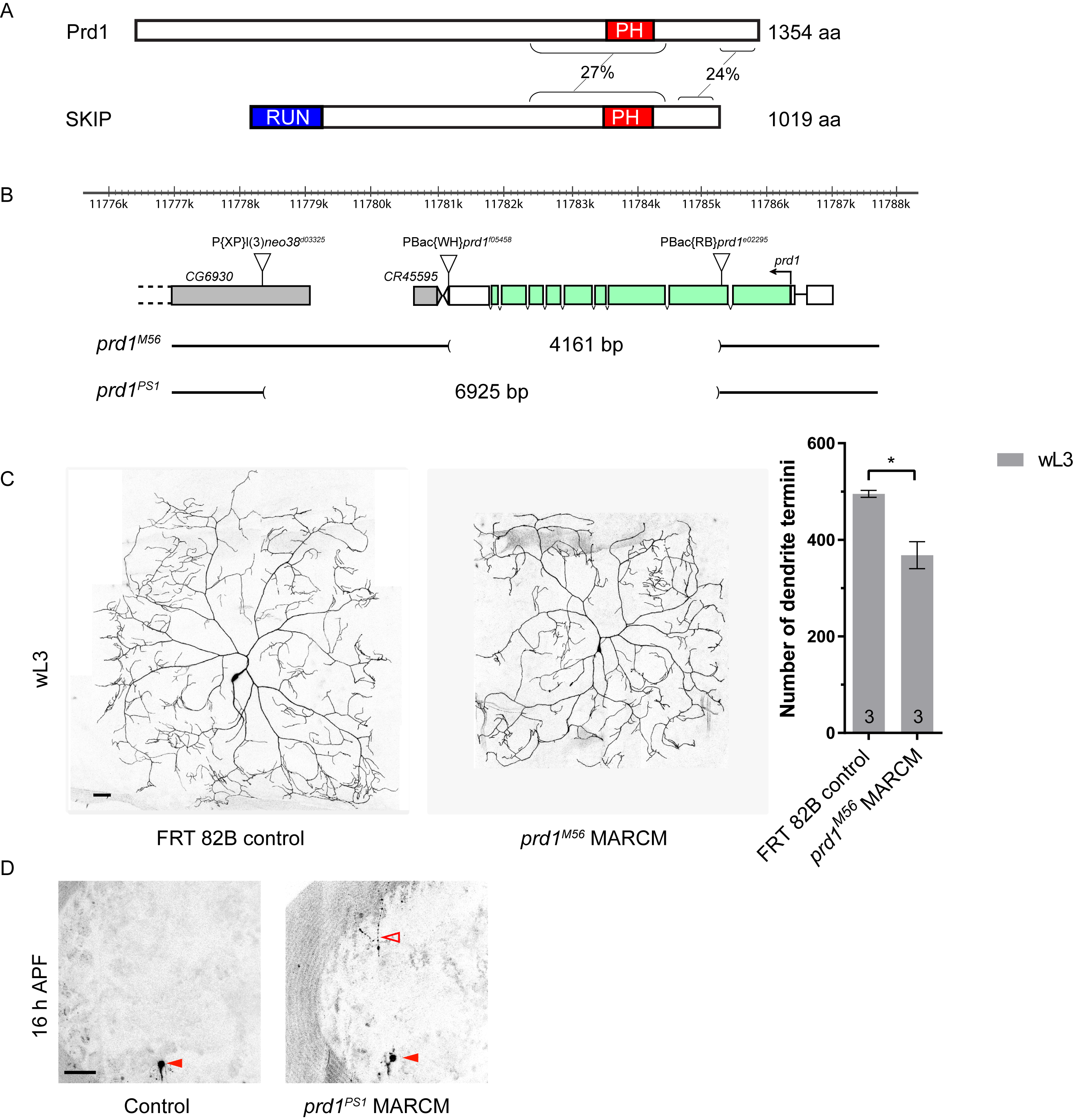

Supplement: S1 Fig — (A) Protein structures of Drosophila Prd1 and mammalian SKIP. The C-terminal portions of Prd1 and SKIP contain a PH domain and share sequence identity (27% and 24%). (B) A schematic diagram of prd1 gene locus and mutants. Those prd1 mutants are derived from three P-element insertion lines, namely PBac{RB}prd1e02295, PBac{WH}prd1f05458, and P{XP}I(3)neo38d03325. (C) Live confocal images of FRT82B control and prd1M56 ddaC MARCM clones at wL3 stage as well as quantification of number of dendrite termini in control and prd1M56 ddaC MARCM clones. (D) Dendrites of control and prd1PS1 MARCM ddaC neurons showing fragmentation defect at 16 h APF. Red arrowheads point to the ddaC somas. Open red arrowhead points to the severed dendrite branches. *p < 0.05 as assessed by two-tailed Student t test. Error bars represent SEM. Scale bars represent 50 μm. The individual numerical values for panel C can be found in S1 Data. The genotypes can be found in S1 Text. APF, after puparium formation; MARCM, mosaic analysis with a repressible cell marker; PH, pleckstrin homology; Prd1, pruning defect 1; SKIP, SifA and Kinesin-interacting protein; wL3, wandering third instar. (TIF) [file pbio.2004506.s001.tif]

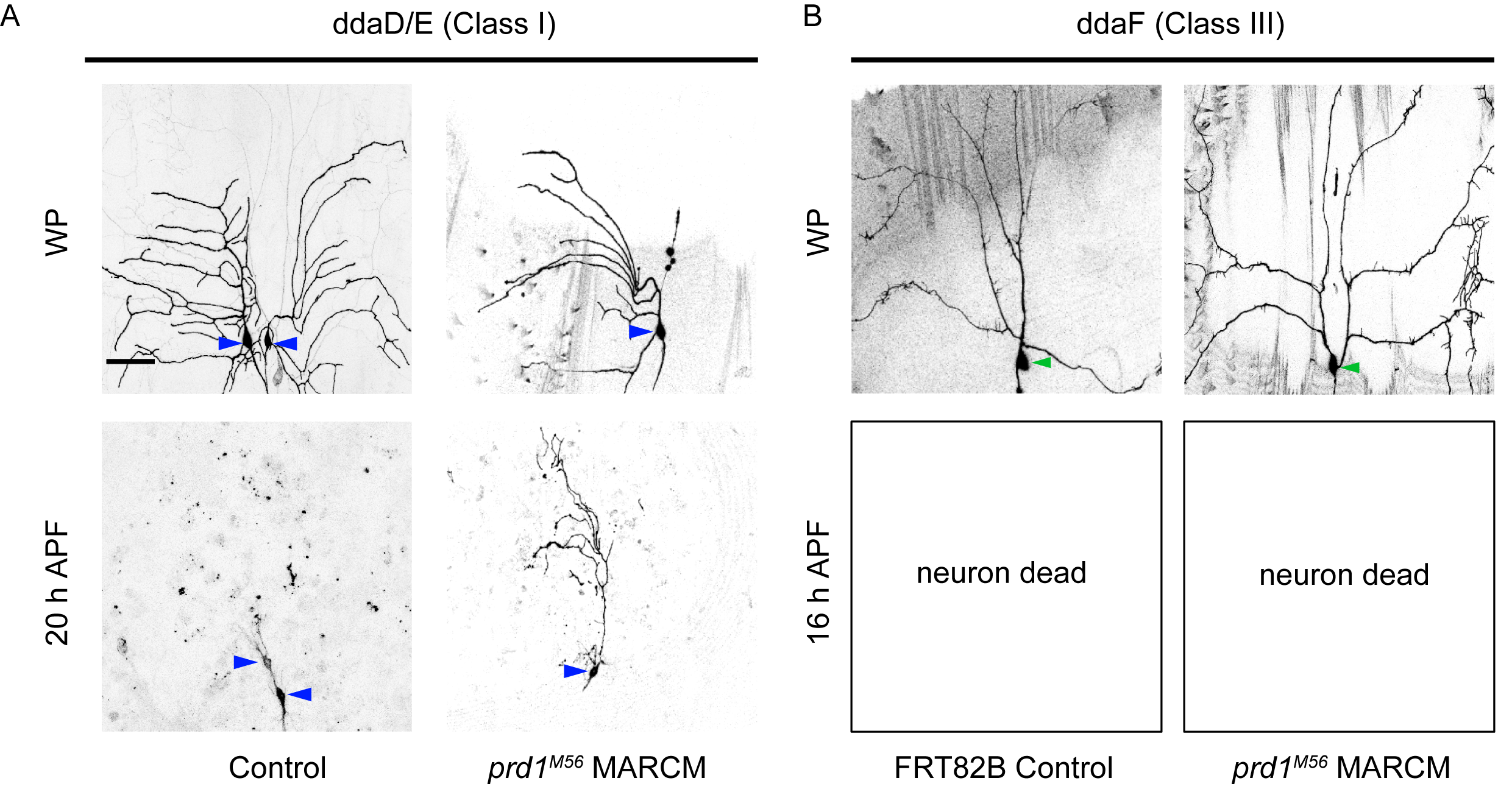

Supplement: S2 Fig — (A) Live confocal images of ddaD/E MARCM clones labeled by UAS-mCD8-GFP at WP and 20 h APF. ddaD/E somas are marked by blue arrowheads. Control ddaD/E MARCM clones pruned all their dendrites at 20 h APF, whereas larval dendrites were still attached to the somas of prd1M56 ddaD/E MARCM clones. (B) Live confocal images of ddaF MARCM clones at WP and 16 h APF. ddaF somas are marked by green arrowheads. prd1M56 ddaF MARCM clones underwent apoptosis, similar to FRT82B control clones. Scale bar (A) represents 50 μm. The genotypes can be found in S1 Text. APF, after puparium formation; MARCM, mosaic analysis with a repressible cell marker; prd1, pruning defect 1; WP, white prepupal. (TIF) [file pbio.2004506.s002.tif]

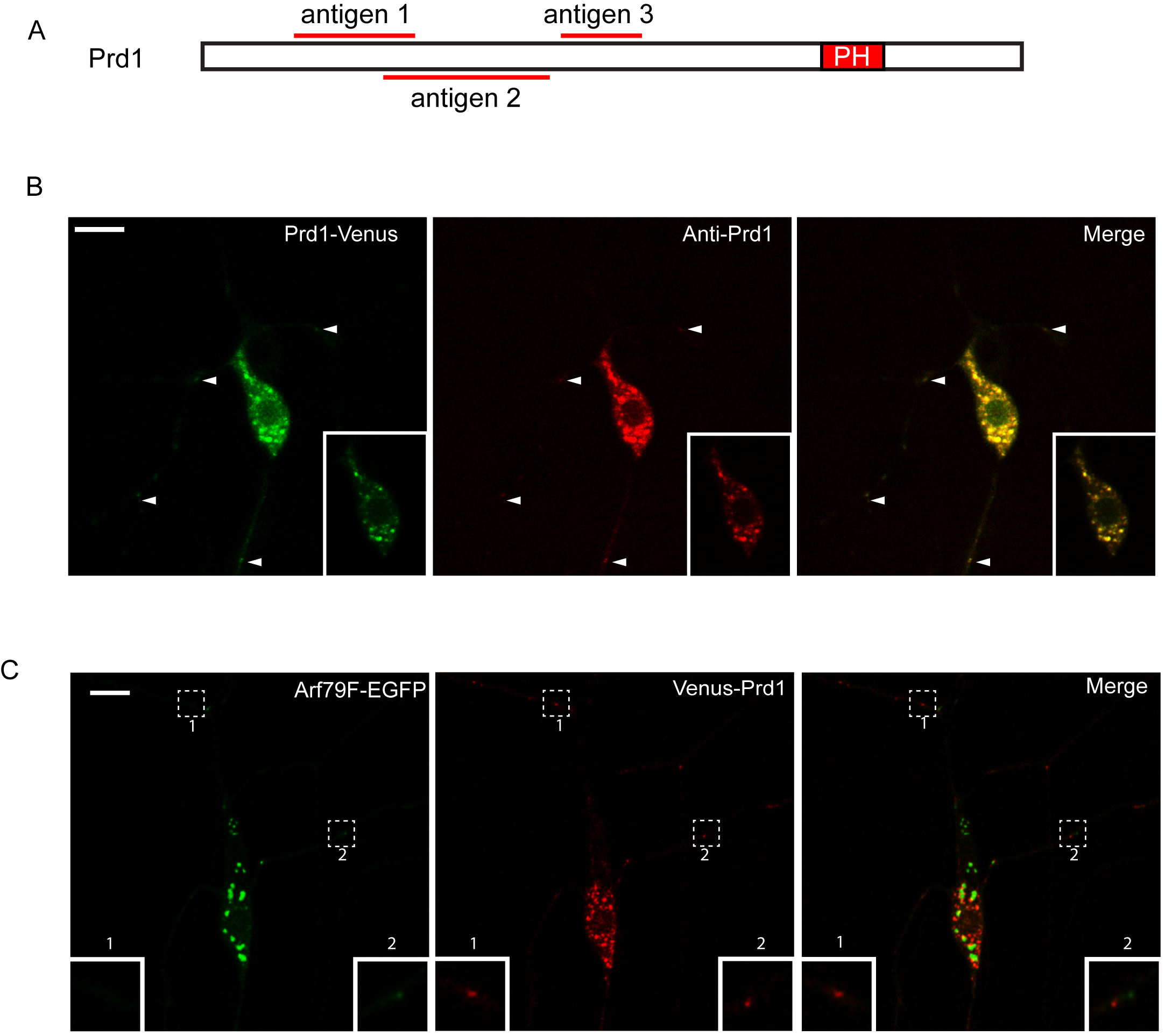

Supplement: S3 Fig — (A) A schematic diagram of Prd1 protein with three antigens indicated in red lines. Antigens 1–3 contain aa151–350, aa300–575, and aa591–724, respectively. (B) Distribution of Venus-Prd1 and anti-Prd1 staining in ddaC neurons expressing Venus-Prd1. Venus-Prd1 colocalized with anti-Prd1 signals in soma, dendrites (arrowheads), and axon (arrowhead). (C) Distribution of Venus-Prd1 and Arf79F-EGFP in ddaC neurons. Venus-Prd1 puncta localized distinctly from Arf79F-EGFP (insets) in dendrites of ddaC neurons. Dorsal is up in all images. Scale bars in (B) and (C) represent 10 μm. The genotypes can be found in S1 Text. Arf79F-EGFP, ADP ribosylation factor 79F fused with enhanced green fluorescent protein; Prd1, Pruning defect 1. (TIF) [file pbio.2004506.s003.tif]

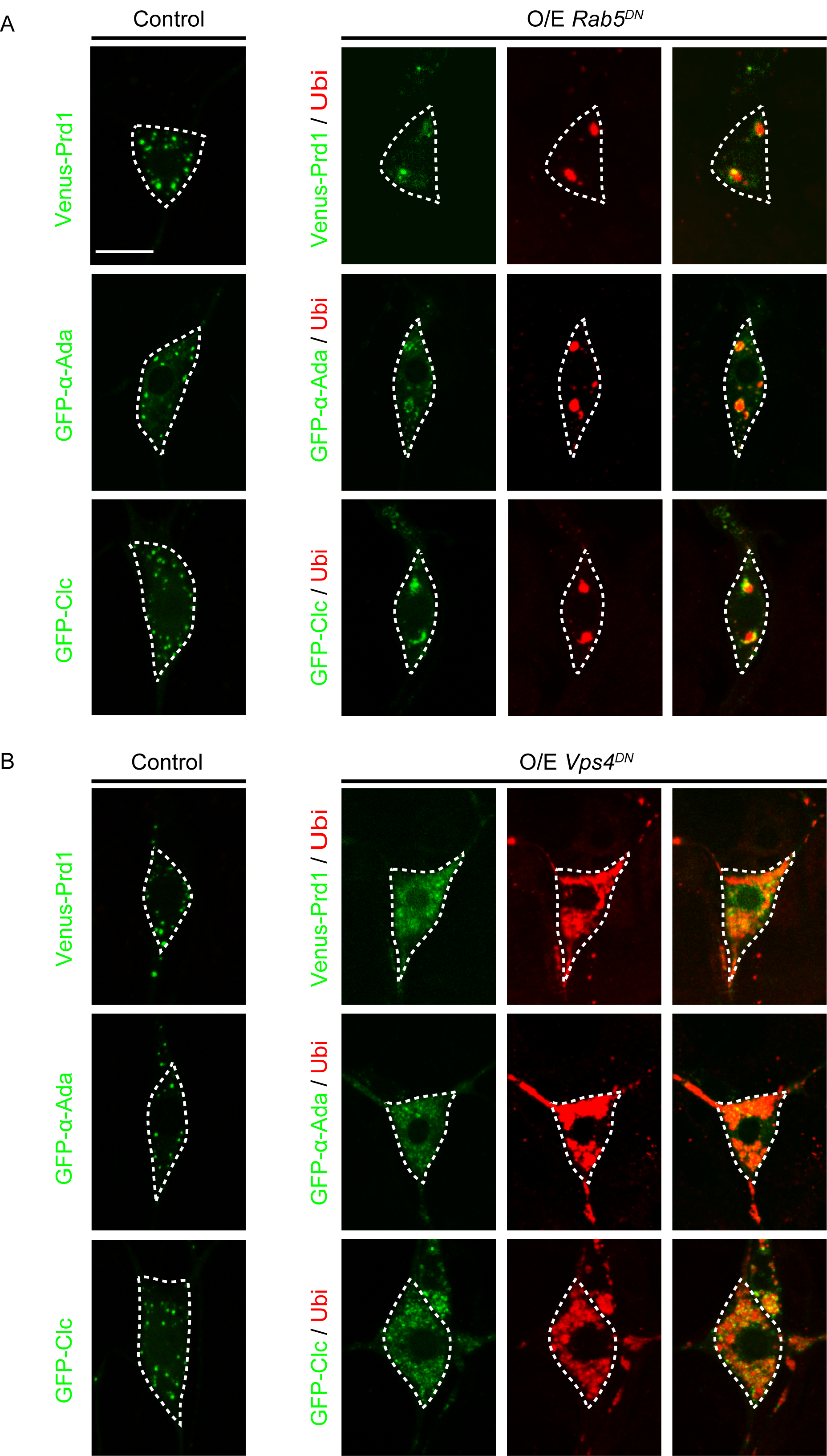

Supplement: S4 Fig — (A–B) Confocal images of control or mutant ddaC neurons at wL3 stage. ddaC somas are marked by dashed lines. Compared with control, Venus-Prd1 enriched on aberrant endosomes in Rab5DN (A) and Vps4DN (B) ddaC neurons. Similarly, GFP-α-Ada and GFP-Clc also accumulated on aberrant ubiquitin-positive endosomes in Rab5DN (A) and Vps4DN (B) ddaC neurons. Scale bar (A) represents 10 μm. The genotypes can be found in S1 Text. GFP-α-Ada, green fluorescent protein fused with α-Adaptin; GFP-Clc, green fluorescent protein fused with Clathrin light chain; Prd1, Pruning defect 1; Rab5DN, Rabaptin-5 dominant-negative form; Vps4DN, Vacuolar protein sorting-associated protein 4 dominant negative form; wL3, wandering third instar. (TIF) [file pbio.2004506.s004.tif]

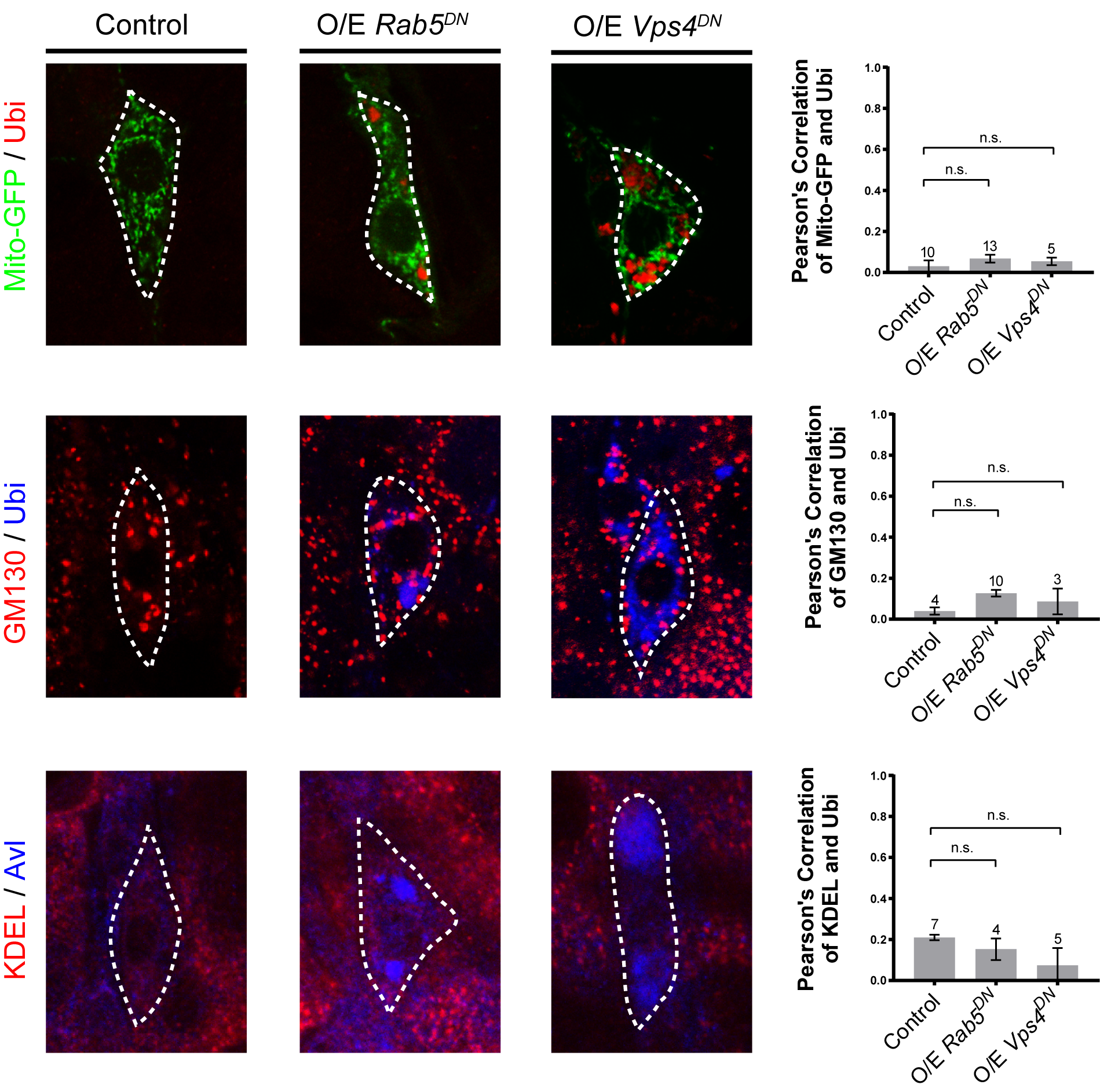

Supplement: S5 Fig — Confocal images of control or mutant ddaC neurons at wL3 stage. Distributions of Mito-GFP, GM130 and KDEL were similar to control ddaC neurons and absent from the aberrant endosomes in Rab5DN or Vps4DN ddaC neurons. Quantifications with Pearson’s correlation coefficients indicate no colocalization between endosomes and these cellular markers. n.s., not significant, as assessed by one-way ANOVA test. ddaC somas are marked by dashed lines. Scale bar represents 10 μm. The individual numerical values for panels can be found in S1 Data. The genotypes can be found in S1 Text. ESCRT, endosomal sorting complexes required for transport; Mito-GFP, mitochondria-GFP; Rab5, Rabaptin-5; wL3, wandering third instar. (TIF) [file pbio.2004506.s005.tif]

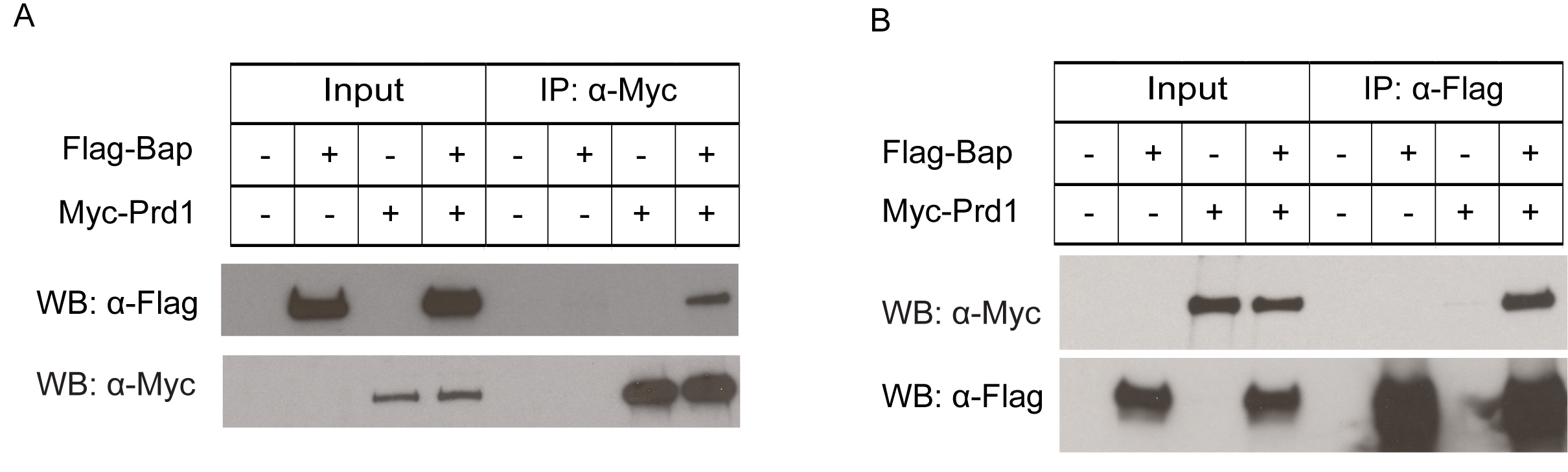

Supplement: S6 Fig — (A–B) Co-IP between Prd1 and Bap. Prd1 and Bap associated with each other in S2 cells co-transfected with Myc-Prd1 and Flag-Bap in co-IP experiments. Bap, β-Adaptin; co-IP, co-immunoprecipitation; Myc-Prd1, Myc-tagged Prd1; Prd1, pruning defect 1. (TIF) [file pbio.2004506.s006.tif]

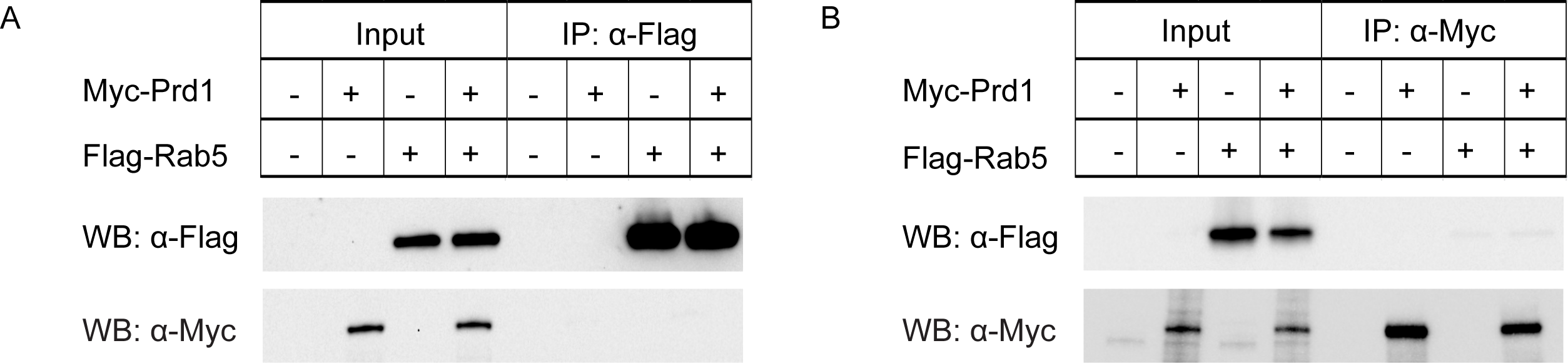

Supplement: S7 Fig — (A–B) Co-IP between Prd1 and Rab5. No interaction between Prd1 and Rab5 was observed in S2 cells transfected with Myc-Prd1 and Flag-Rab5. co-IP, co-immunoprecipitation; Myc-Prd1, Myc-tagged Prd1; Prd1, pruning defect 1; Rab5, Rabaptin-5. (TIF) [file pbio.2004506.s007.tif]

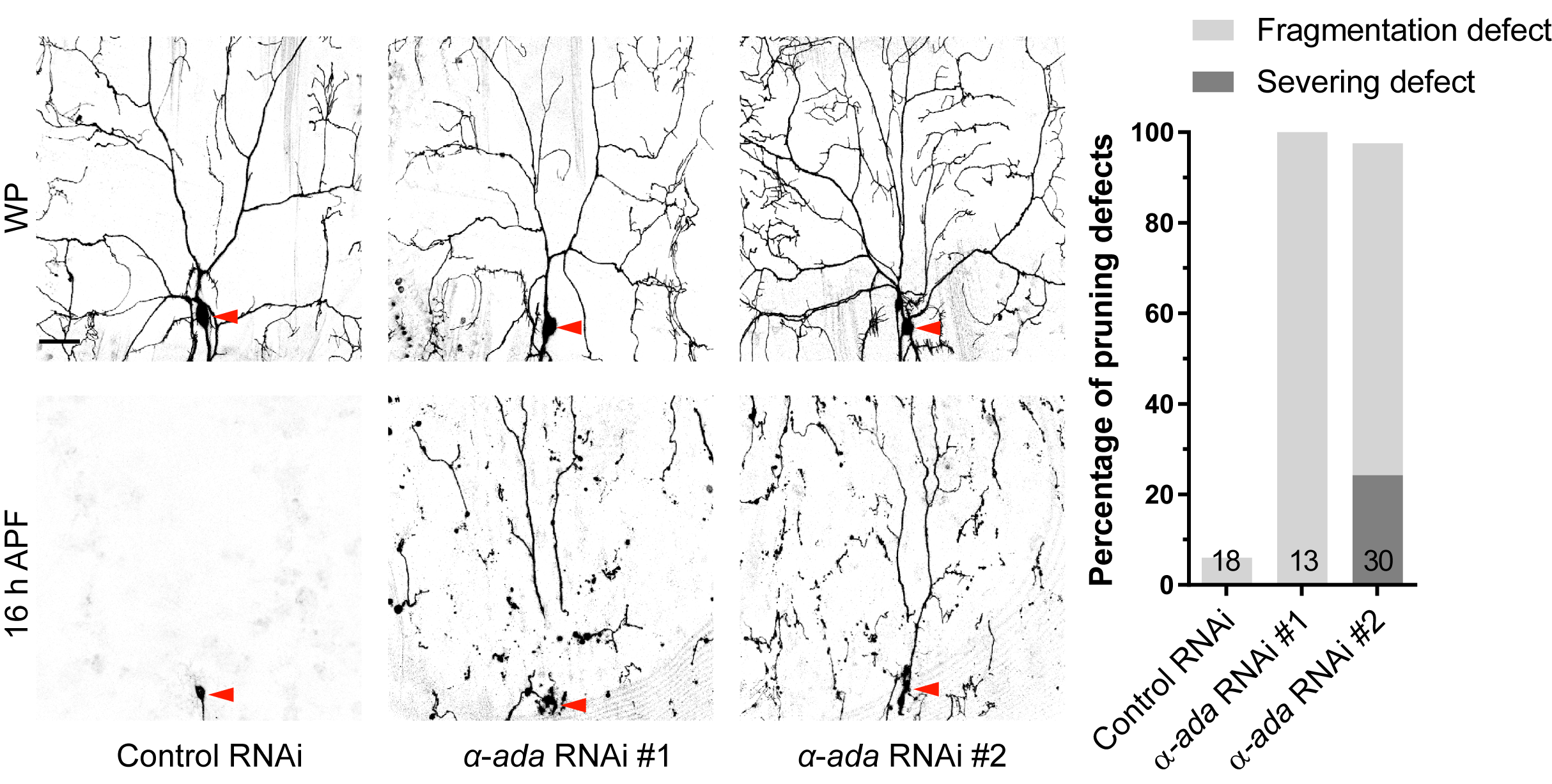

Supplement: S8 Fig — Live confocal images of ddaC neurons expressing mCD8-GFP at WP and 16 h APF. Red arrowheads indicate ddaC somas. All dendrites of control ddaC neurons are pruned at 16 h APF; however, dendrites failed to be pruned in α-ada RNAi ddaC neurons. The number of samples (n) in each group is shown on the bars. Scale bar represents 50 μm. Dorsal is up in all images. The individual numerical values for panels can be found in S1 Data. The genotypes can be found in S1 Text. α-ada, α-adaptin; APF, after puparium formation; mCD8-GFP, membrane-associated green fluorescent protein; RNAi, RNA interference; WP, white prepupal. (TIF) [file pbio.2004506.s008.tif]

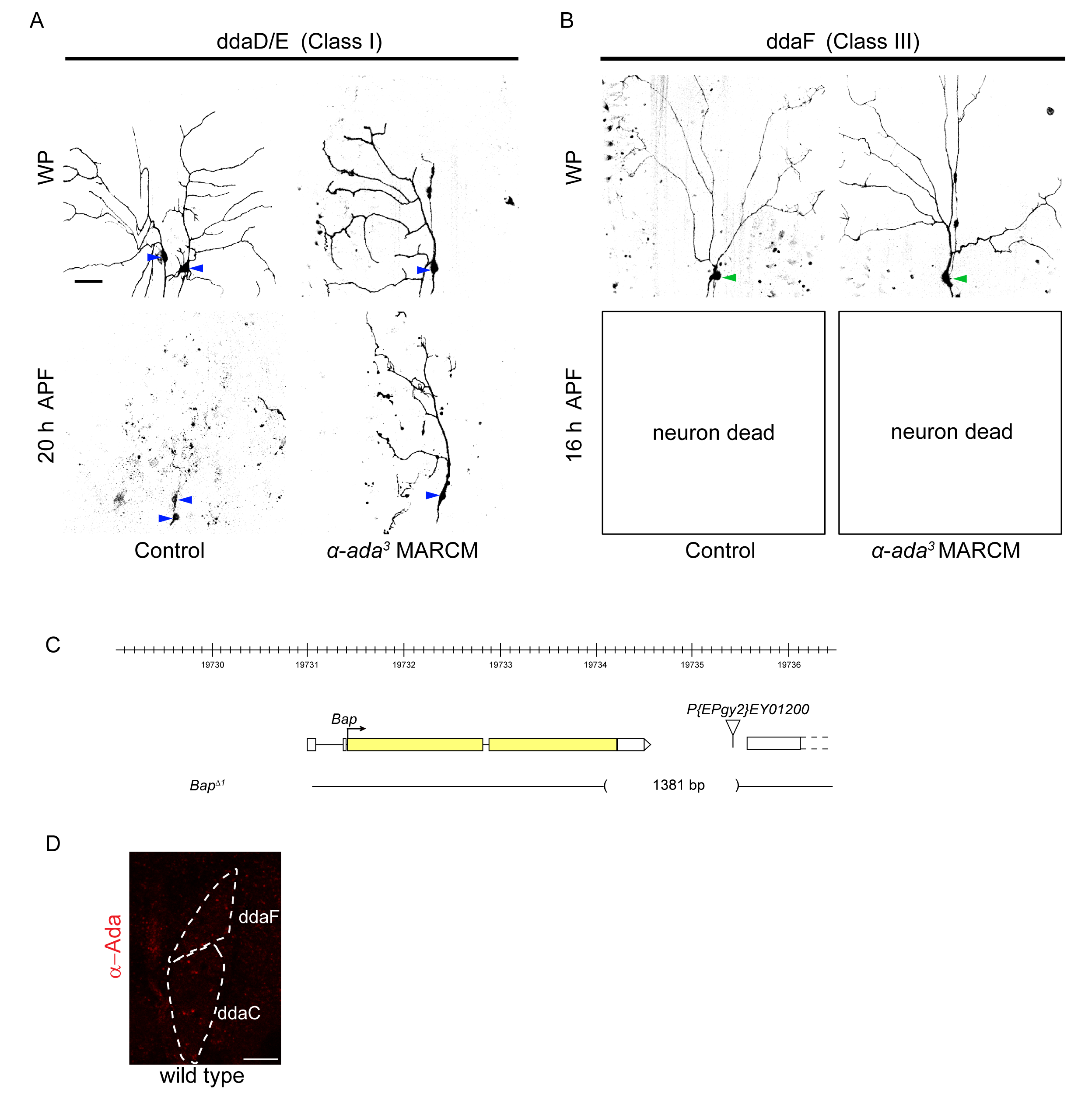

Supplement: S9 Fig — (A) Live confocal images of ddaD/E neurons at WP and 20 h APF. ddaD/E somas are marked by blue arrowheads. Control ddaD/E neurons pruned all dendrites, while some larval dendrites of α-ada3 ddaD/E clones remained attached to the somas at 20 h APF. (B) ddaF MARCM clones at WP and 16 h APF. ddaF somas are marked by green arrowheads. Similar to the control, α-ada3 ddaF MARCM clones undergo apoptosis during early metamorphosis. (C) A schematic diagram of the Bap gene and the deleted region of the BapΔ1 mutant. BapΔ1 was generated by the P-element insertion P{EPgy2}EY01200. (D) Expression of α-Ada protein in sensory neurons. ddaC and ddaF somas are marked by dashed lines. Scale bars (A) and (D) represent 50 μm and 10 μm, respectively. Dorsal is up in all images. The genotypes can be found in S1 Text. α-Ada, α-Adaptin; APF, after puparium formation; MARCM, mosaic analysis with a repressible cell marker; WP, white prepupal. (TIF) [file pbio.2004506.s009.tif]

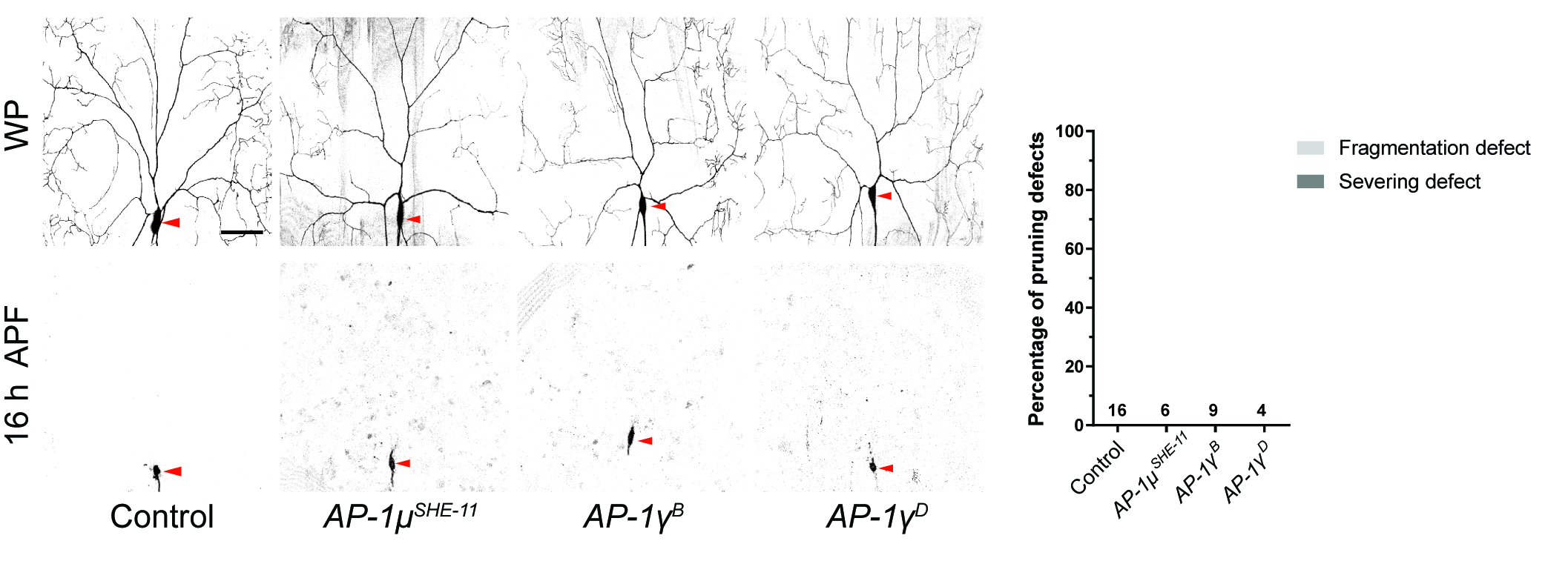

Supplement: S10 Fig — Live confocal images of control, AP-1μSHE-11, AP-1γB, and AP-1γD ddaC neurons at WP and 16 h APF. ddaC somas are marked by red arrowheads. Quantification analysis of percentage of severing defect and fragmentation defect in control and mutant ddaC neurons at 16 h APF. The number of samples (n) in each group is shown on the bars. Scale bar represents 50 μm. The individual numerical values for panel can be found in S1 Data. The genotypes can be found in S1 Text. AP-1μ, Adaptor protein-1 μ subunit; AP-1γ, Adaptor protein-1 γ subunit; APF, after puparium formation; WP, white prepupal. (TIF) [file pbio.2004506.s010.tif]

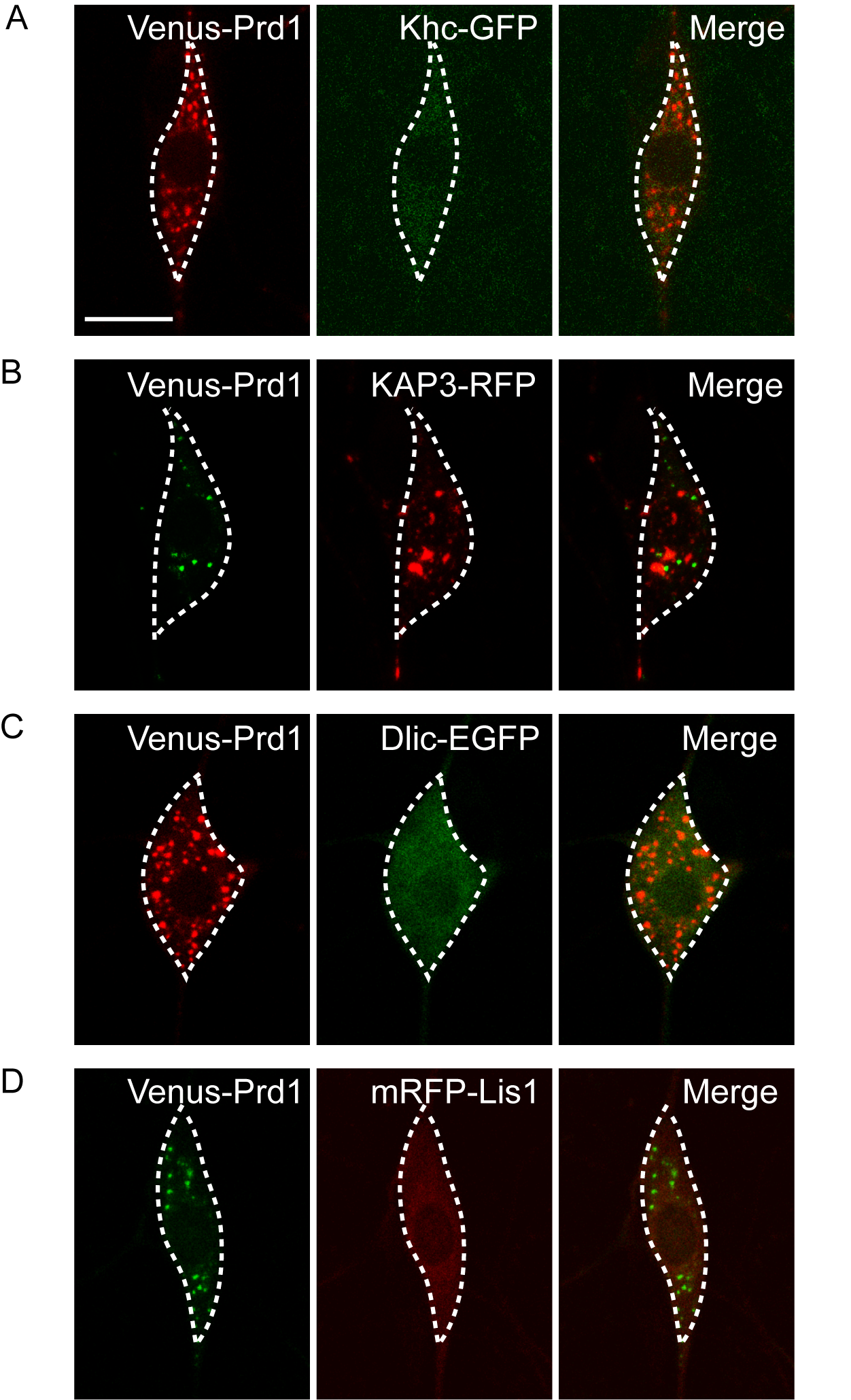

Supplement: S11 Fig — (A–D) Distribution of Prd1 and kinesin/dynein motors. Khc-GFP (A), Kap3-RFP (B), Dlic-EGFP (C), or mRFP-Lis1 (D) showed distinct pattern with Venus-Prd1 in ddaC neurons. ddaC somas are marked by dashed lines. Scale bar (A) represents 10 μm. Dorsal is up in all images. The genotypes can be found in S1 Text. Dlic-EGFP, Dlic fused with enhanced green fluorescent protein; Kap3-RFP, Kinesin associated protein 3 fused with red fluorescent protein; Khc-GFP, Kinesin heavy chain fused with green fluorescent protein; mRFP-Lis1, monomeric red fluorescent protein fused with Lis1; Prd1, Pruning defect 1. (TIF) [file pbio.2004506.s011.tif]

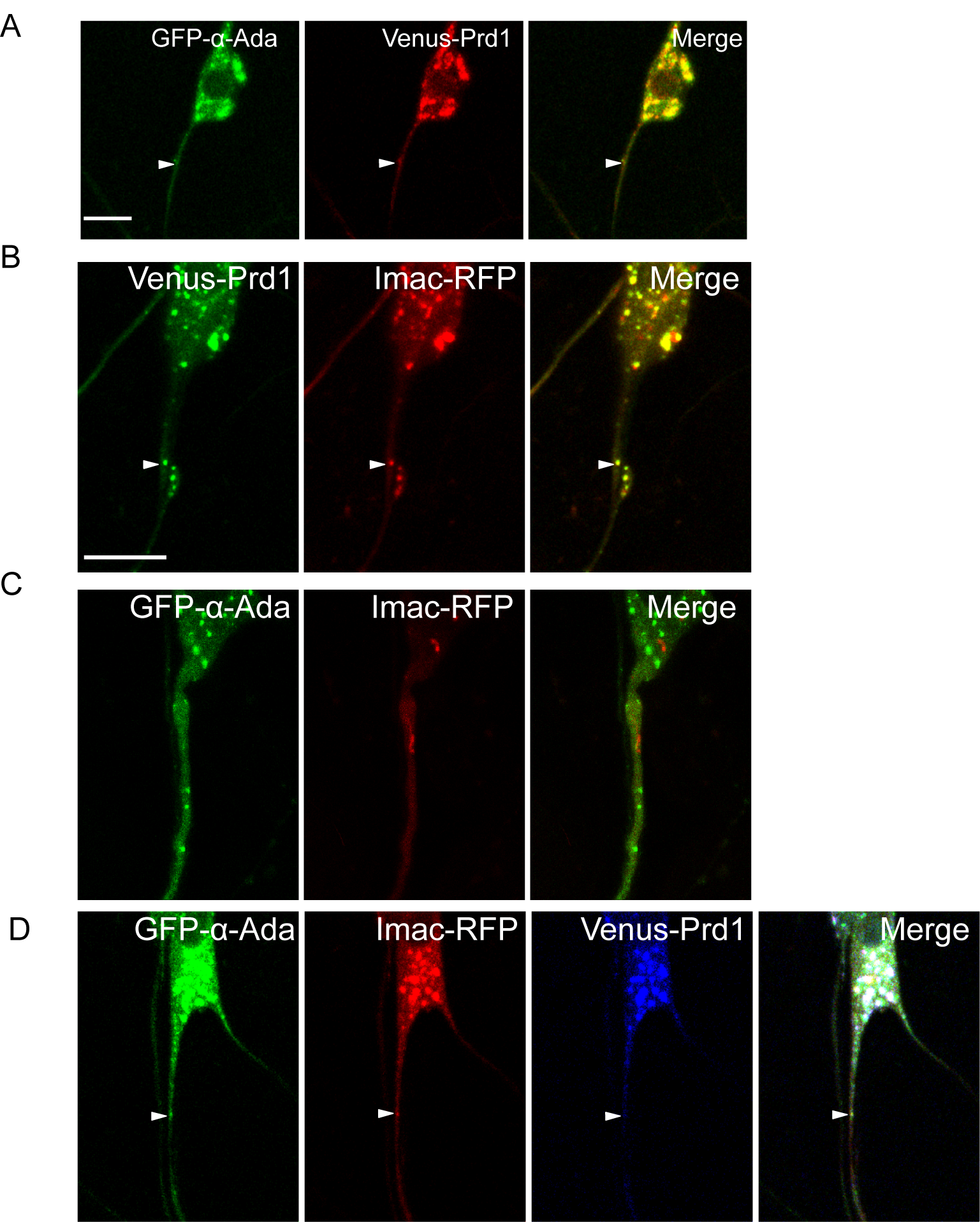

Supplement: S12 Fig — (A) Distribution of GFP-α-Ada and Venus-Prd1 in ddaC axons. Venus-Prd1 colocalized with GFP-α-Ada in the axons (arrowhead). (B) Distribution of Venus-Prd1 and Imac-RFP in ddaC axons. Venus-Prd1 colocalized with Imac-RFP in the axons (arrowhead). (C) Distribution of GFP-α-Ada and Imac-RFP in ddaC axons. GFP-α-Ada puncta did not overlap with Imac-RFP in the axons. (D) Distribution of Venus-Prd1, GFP-α-Ada, and Imac-RFP in ddaC axons. Venus-Prd1, GFP-α-Ada, and Imac-RFP colocalized in ddaC axons (arrowhead). Scale bars represent 10 μm. The genotypes can be found in S1 Text. α-Ada, α-Adaptin; GFP, green fluorescent protein; Imac-RFP, immaculate connections fused with red fluorescent protein; Prd1, Pruning defect 1. (TIF) [file pbio.2004506.s012.tif]

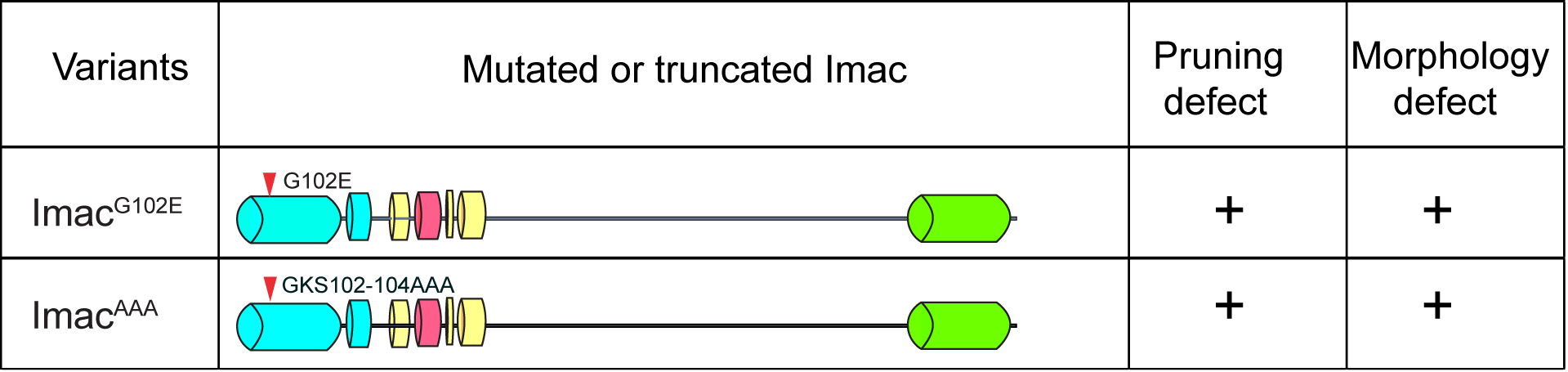

Supplement: S13 Fig — Imac contains a motor domain (blue), coiled coil domains 1–3 (yellow), FHA domain (pink), and a PH domain (green). FHA, forkhead-associated; imac, immaculate connections; PH, pleckstrin homology. (TIF) [file pbio.2004506.s013.tif]

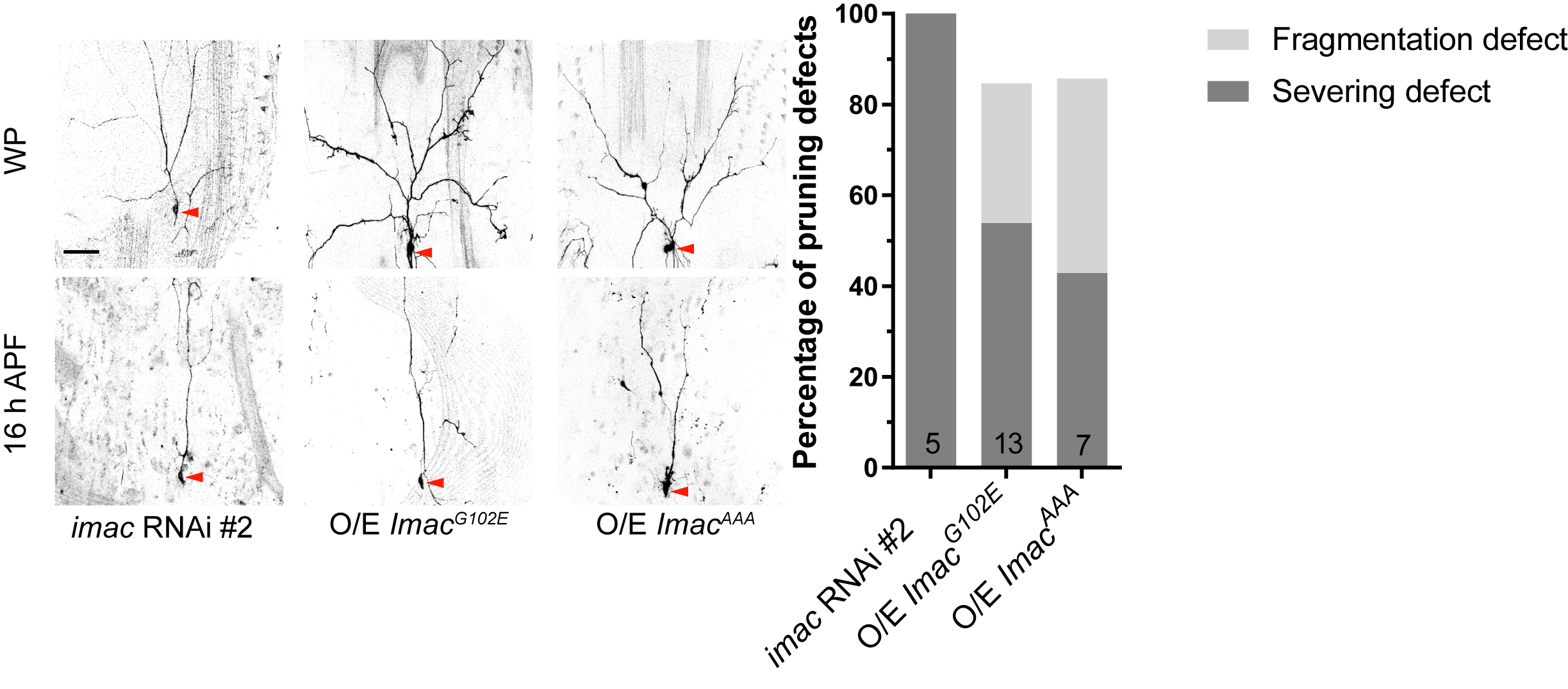

Supplement: S14 Fig — Live confocal images of ddaC neurons labeled by mCD8-GFP at WP and 16 h APF. ddaC somas are indicated by red arrowheads. Dendrites of imac RNAi #2, ImacG102E, or ImacAAA mutant ddaC neurons remained attached to their somas at 16 h APF. Quantification of severing defect and fragmentation defect in imac mutant ddaC neurons at 16 h APF. The number of samples (n) in each group is shown on the bars. Scale bar represents 50 μm. Dorsal is up in all images. The individual numerical values for panels can be found in S1 Data. The genotypes can be found in S1 Text. APF, after puparium formation; imac, immaculate connections; RNAi, RNA interference; WP, white prepupal. (TIF) [file pbio.2004506.s014.tif]

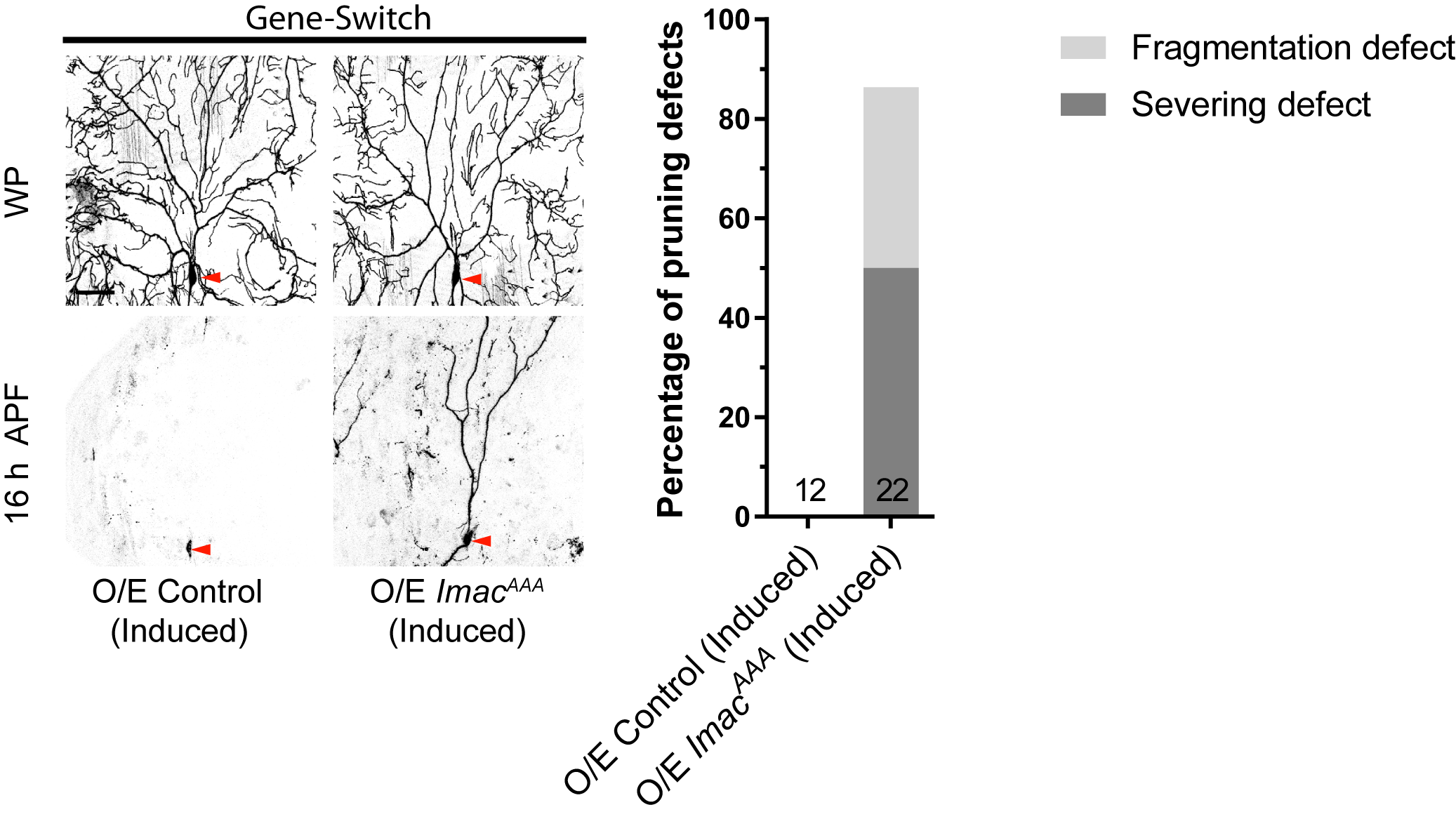

Supplement: S15 Fig — Using the Gene-Switch system, inducible expression of ImacAAA at the eL3 stage caused severe dendrite pruning defects at 16 h APF, whereas inducible expression of the control UAS transgene showed normal dendrite pruning. Quantification of severing defect and fragmentation defect in mutant ddaC neurons at 16 h APF. The number of samples (n) in each group is shown on the bars. Scale bar represents 50 μm. Dorsal is up in all images. The individual numerical values for panels can be found in S1 Data. The genotypes can be found in S1 Text. APF, after puparium formation; eL3, early third instar. (TIF) [file pbio.2004506.s015.tif]

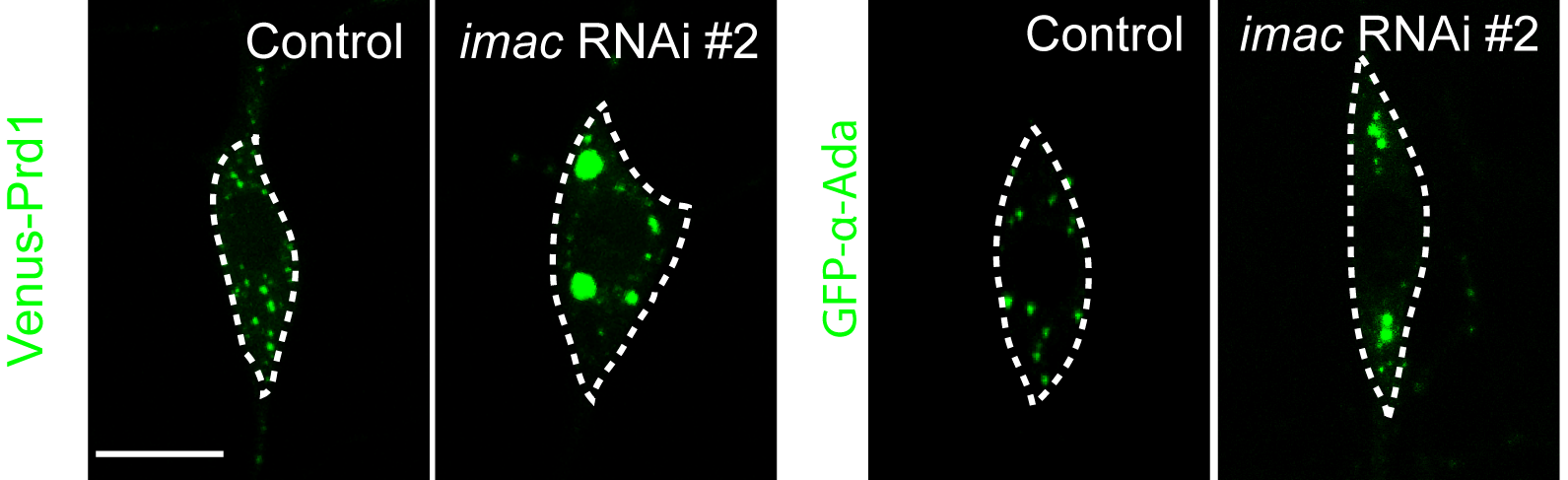

Supplement: S16 Fig — Distribution of Venus-Prd1 (in green) and GFP-α-Ada (in green) in control and imac RNAi (#2) ddaC neurons. ddaC somas are marked by dashed lines. Scale bar represents 10 μm. Dorsal is up in all images. The genotypes can be found in S1 Text. GFP-α-Ada, green fluorescent protein fused with α-Adaptin; imac, immaculate connections; RNAi, RNA interference; Venus-Prd1, Venus-Pruning defect 1. (TIF) [file pbio.2004506.s016.tif]

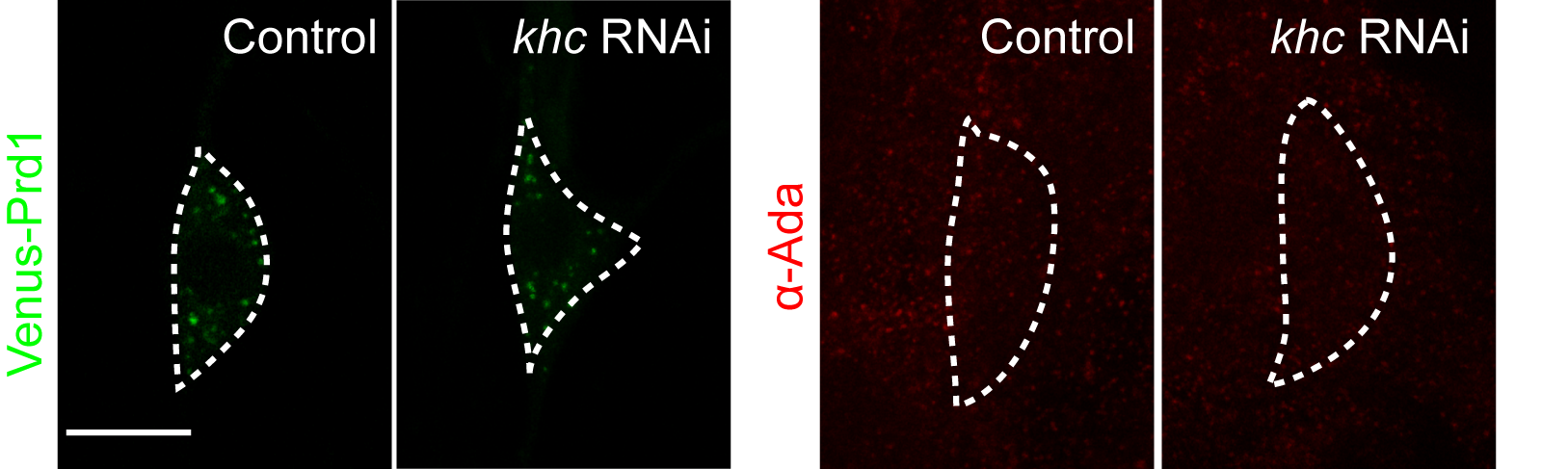

Supplement: S17 Fig — Distribution of Venus-Prd1 (in green) and α-Ada (in red) in control and khc RNAi ddaC neurons. ddaC somas are marked by dashed lines. Scale bar represents 10 μm. Dorsal is up in all images. The genotypes can be found in S1 Text. α-Ada, α-Adaptin; khc, kinesin heavy chain; Prd1, Pruning defect 1; RNAi, RNA interference. (TIF) [file pbio.2004506.s017.tif]

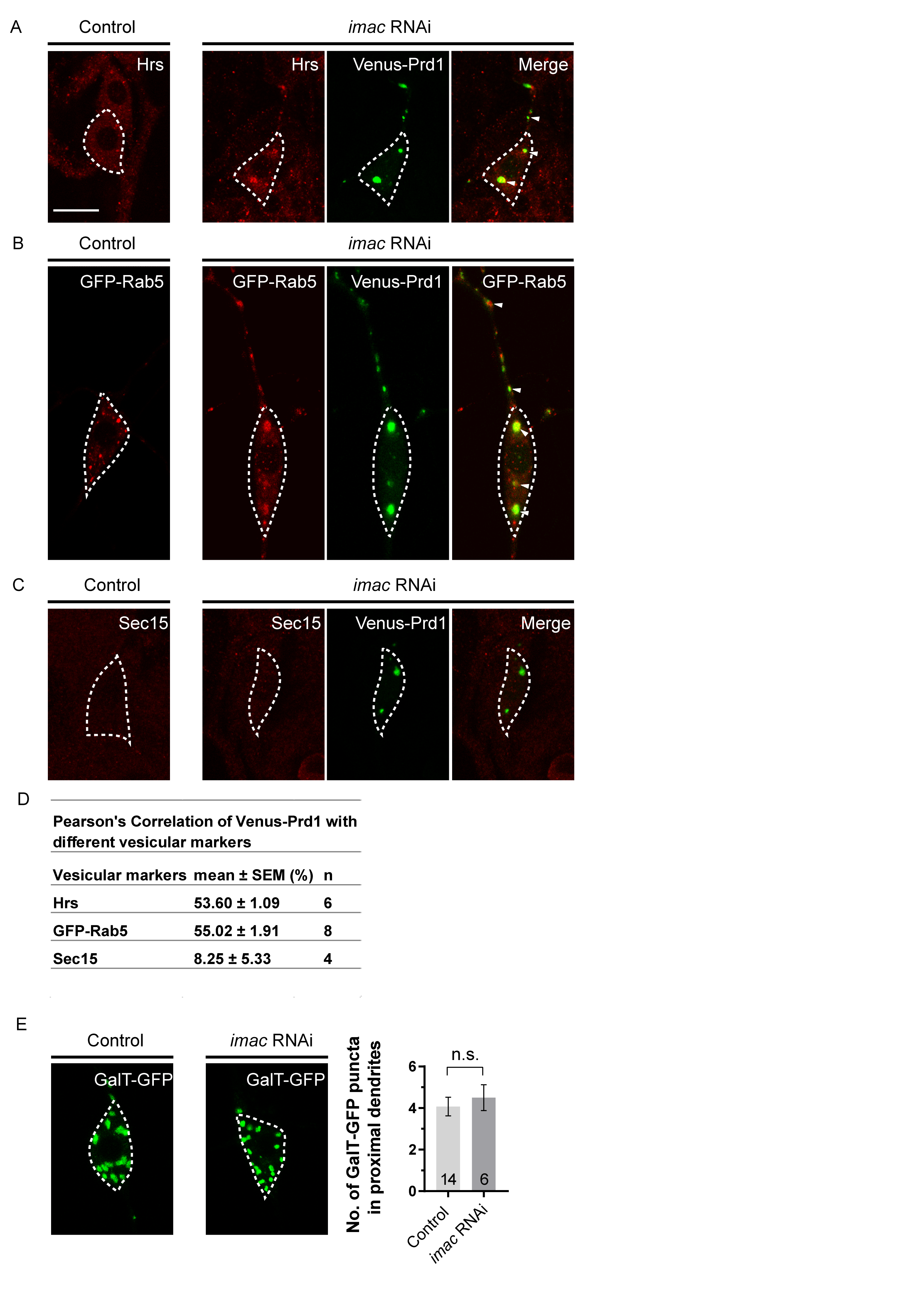

Supplement: S18 Fig — (A) Distribution of the endosomal marker Hrs (in red) in control and imac RNAi (#1) ddaC neurons overexpressing Venus-Prd1 (in green). (B) Distribution of the early endosomal marker GFP-Rab5 (in red) in control and imac RNAi (#1) ddaC neurons overexpressing Venus-Prd1 (in green). (C) Distribution of the secretory marker Sec15 (in red) in control and imac RNAi (#1) ddaC neurons overexpressing Venus-Prd1 (in green). (D) The table shows the colocalization ratios (Pearson’s correlation coefficients) of Venus-Prd1 with different vesicular markers in imac RNAi neurons. n represents the number of neurons examined in each group. (E) Distribution of GalT-GFP (in green) in control and imac RNAi (#1) ddaC neurons. ddaC somas are marked by dashed lines. Scale bar (A) represents 10 μm. Dorsal is up in all images. The individual numerical values for panels D and E can be found in S1 Data. The genotypes can be found in S1 Text. GalT-GFP, GalT fused with GFP; Hrs, hepatocyte growth factor-regulated tyrosine kinase substrate; Rab5, Rabaptin 5; RNAi, RNA interference. (TIF) [file pbio.2004506.s018.tif]

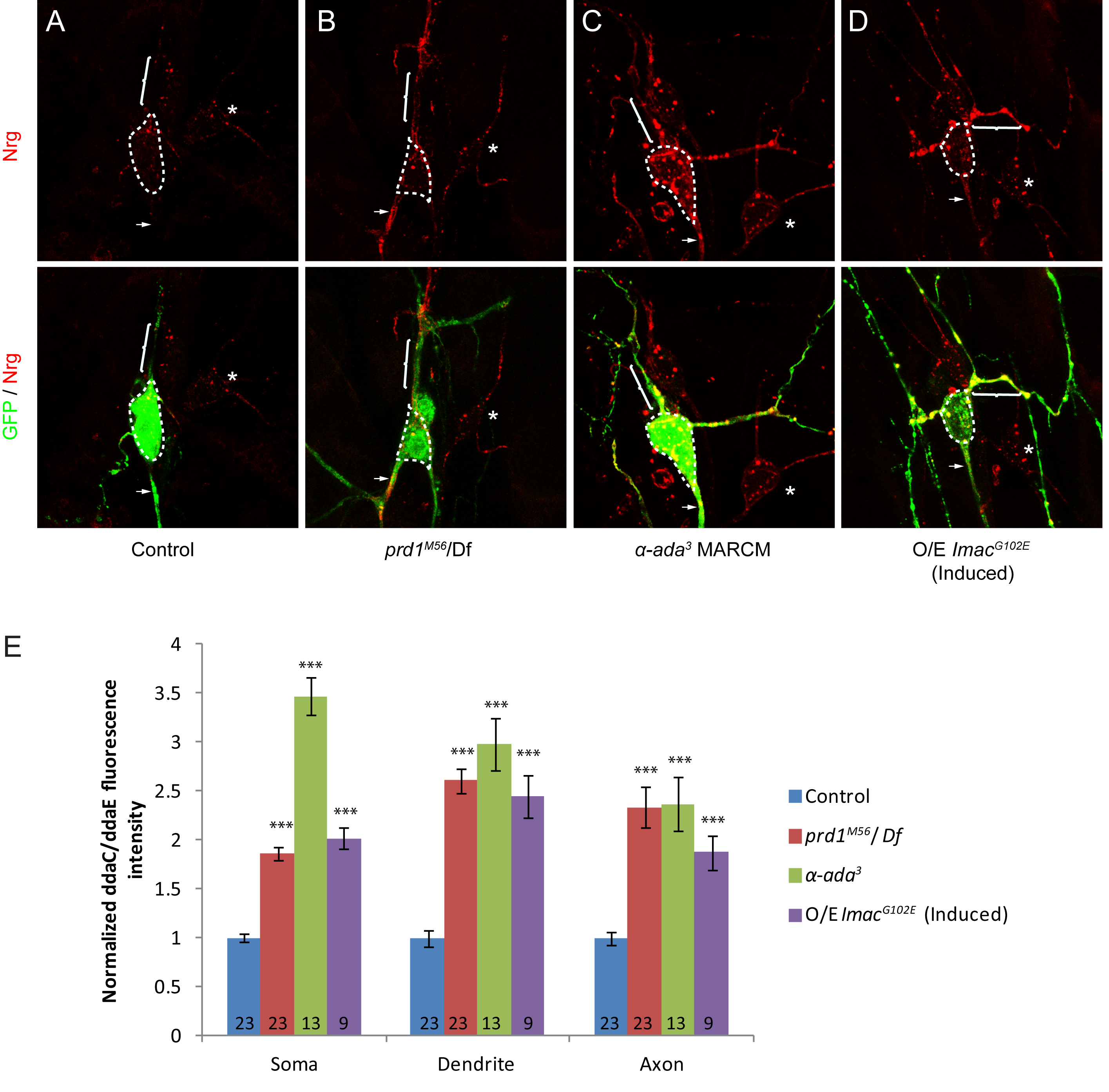

Supplement: S19 Fig — (A–D) The distribution of Nrg in control (A), prd1M56/Df (3R)Exel7310 (B), α-ada3 MARCM (C), or imacG102E (induced) (D) ddaC neurons at 6 h APF. ddaC somas are marked by dashed lines, axons by arrows, and proximal dendrites by curly brackets. ddaE somas are marked by asterisks. (E) Quantification of Nrg immunostaining intensity. The number of samples (n) in each group is shown on the bars. Error bars represent SEM. ***p < 0.001 as assessed by one-way ANOVA test. Dorsal is up in all images. The individual numerical values for panel E can be found in S1 Data. The genotypes can be found in S1 Text. α-ada, α-adaptin; APF, after puparium formation; imac, immaculate connections; MARCM, mosaic analysis with a repressible cell marker; Nrg, Neuroglian; prd1, pruning defect 1. (TIF) [file pbio.2004506.s019.tif]

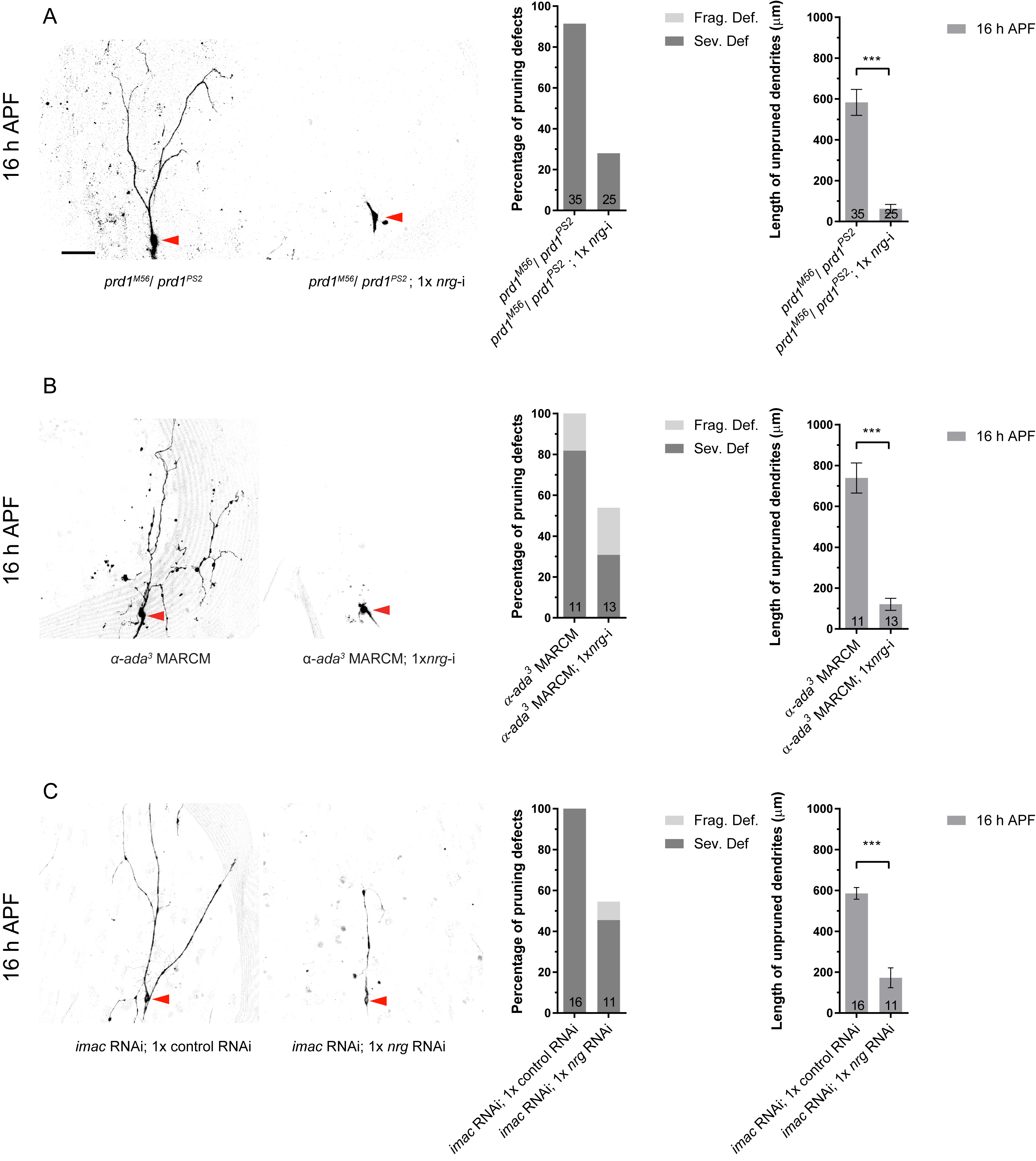

Supplement: S20 Fig — (A–C) Live confocal images of ddaC neurons expressing UAS-mCD8-GFP driven by ppk-Gal4 at 16 h APF. nrg RNAi knockdown significantly rescued pruning defects of prd1M56/prd1PS2 (A), α-ada3 MARCM (B), or imac RNAi (C) ddaC neurons at 16 h APF. ddaC somas are marked by red arrowheads. Quantification analysis of percentage of severing defect and fragmentation defect in control and mutant ddaC neurons at 16 h APF. Quantification of total length of unpruned ddaC dendrites at 16 h APF. The number of samples (n) in each group is shown on the bars. Error bars represent SEM. ***p < 0.001 as assessed by one-way ANOVA test. Scale bar represents 50 μm. The individual numerical values for panels A, B, and C can be found in S1 Data. The genotypes can be found in S1 Text. α-ada, α-adaptin; APF, after puparium formation; imac, immaculate connections; MARCM, mosaic analysis with a repressible cell marker; nrg, neuroglian; prd1, pruning defect 1; RNAi, RNA interference. (TIF) [file pbio.2004506.s020.tif]

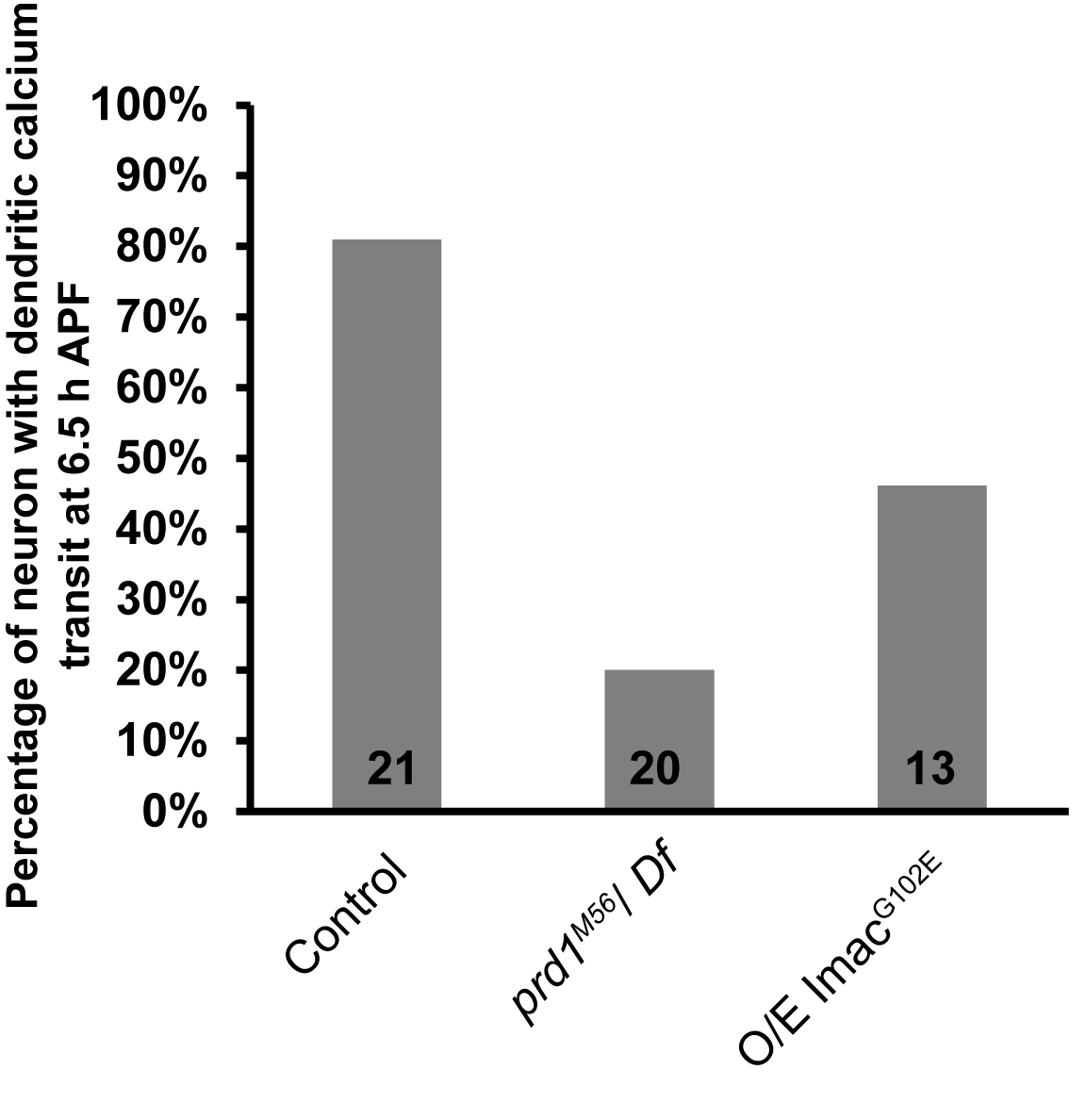

Supplement: S21 Fig — The percentage of neurons with dendritic calcium transients was reduced at 6.5 h APF in prd1M56/Df(3R)Exel7310 and ImacG102E mutant neurons, compared to the control neurons. The number of samples (n) in each group is shown on the bars. The individual numerical values for panels can be found in S1 Data. The genotypes can be found in S1 Text. APF, after puparium formation; imac, immaculate connections; prd1, pruning defect 1. (TIF) [file pbio.2004506.s021.tif]

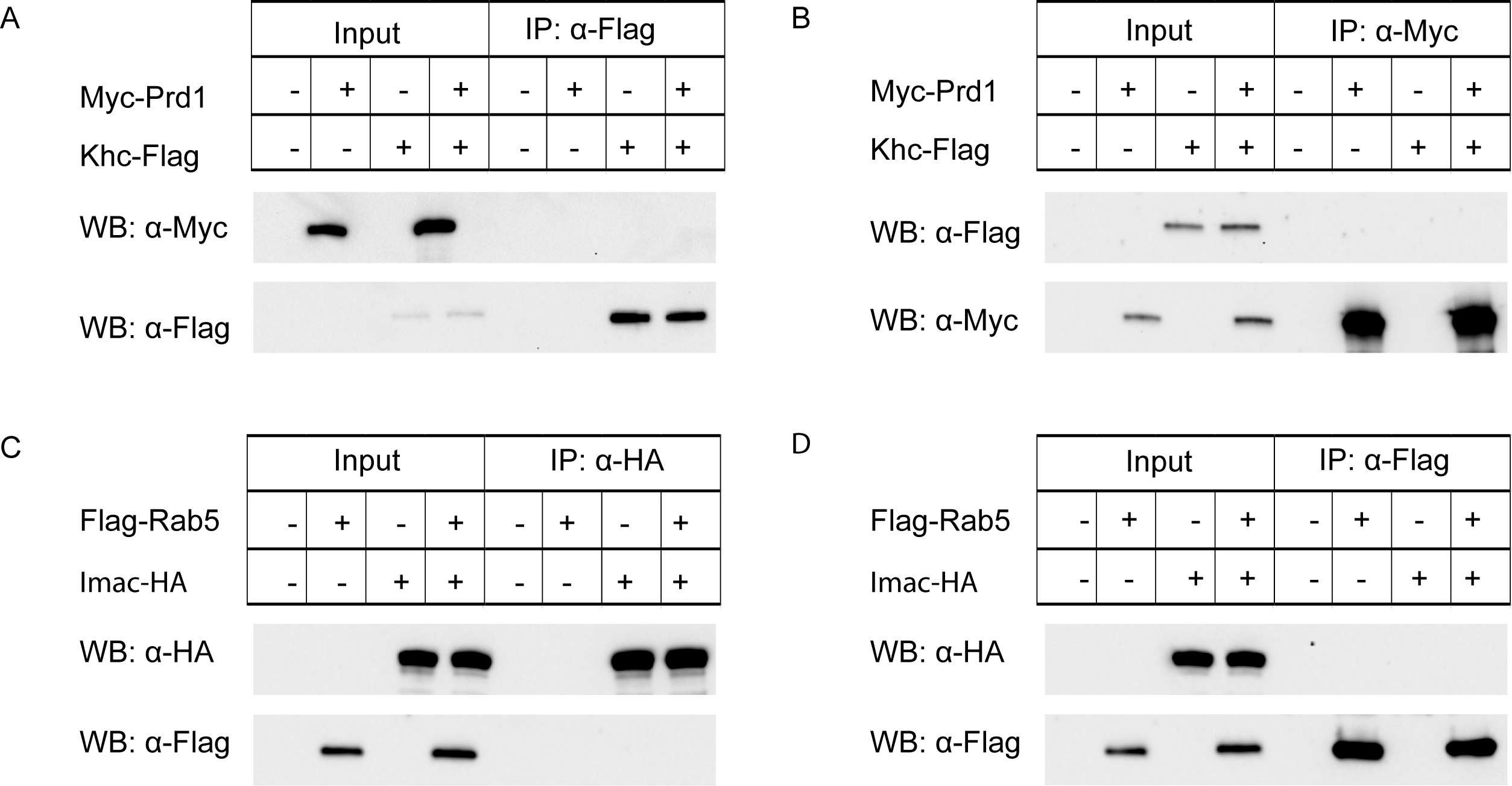

Supplement: S22 Fig — (A–B) Co-IP between Prd1 and Khc. Prd1 did not interact with Khc when S2 cell extracts were immunoprecipitated with anti-Flag (A) or anti-Myc (B) antibody. (C–D) Co-IP between Imac and Rab5. In S2 cell extracts co-transfected with Imac-HA and Flag-Rab5, no interaction was detected when immunoprecipitated with anti-HA (C) or anti-Flag (D) antibody. co-IP, co-immunoprecipitation; HA, HA tag; Imac, Immaculate connections; Khc, Kinesin heavy chain; Myc, Myc tag; Prd1, Pruning defect 1; Rab5, Rabaptin 5. (TIF) [file pbio.2004506.s022.tif]

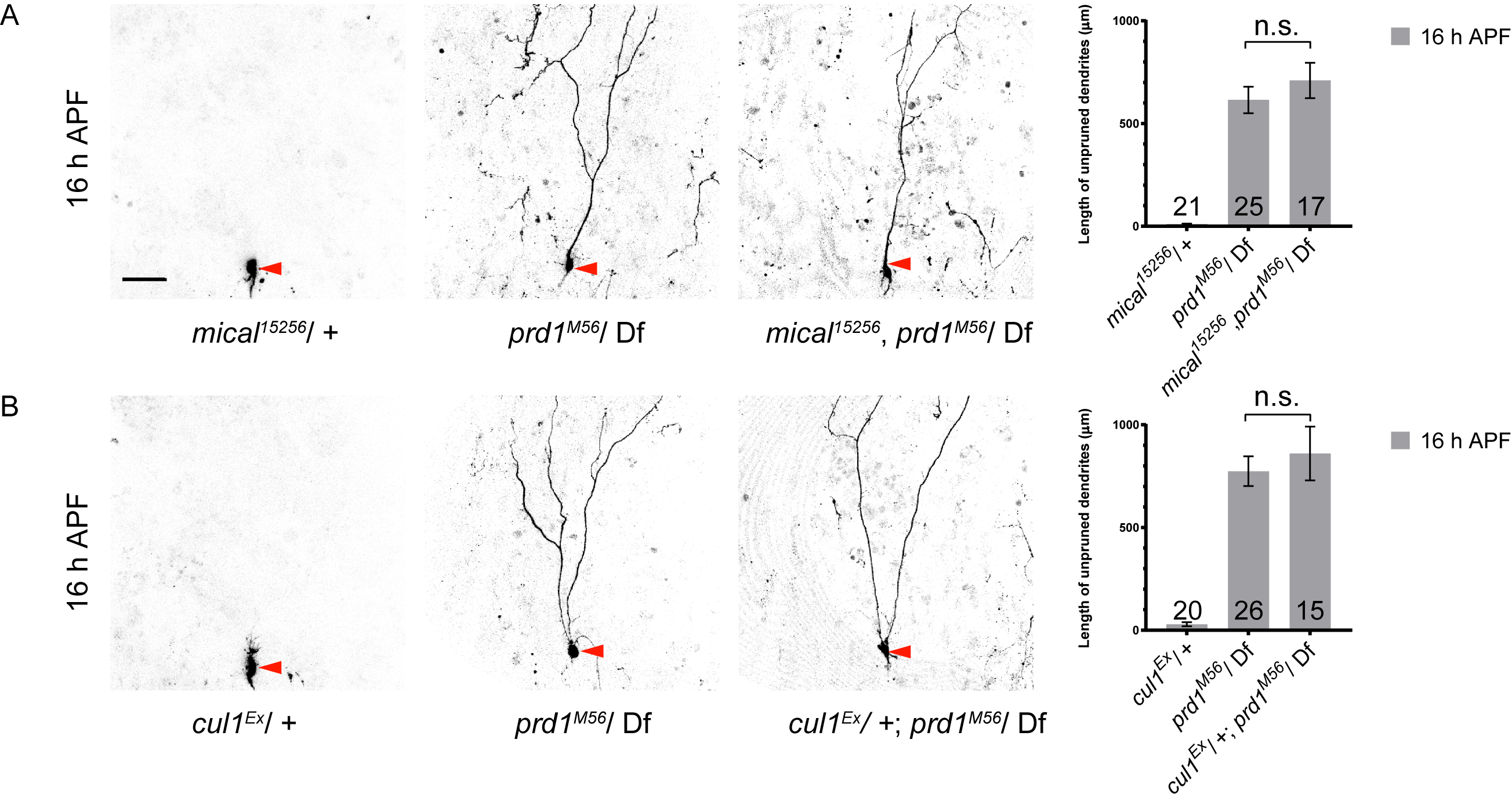

Supplement: S23 Fig — (A) Dendrites of mical15256/+, prd1M56/ Df(3R)Exel7310, and mical15256, prd1M56/ Df(3R)Exel7310 ddaC neurons at 16 h APF. (B) Dendrites of cul1Ex/+, prd1M56/Df(3R)Exel7310, and cul1Ex/+; prd1M56/Df(3R)Exel7310 ddaC neurons at 16 h APF. Quantification of total length of unpruned dendrites at 16 h APF. The number of samples (n) in each group is shown on the bars. Error bars represent SEM. Scale bar (A) represents 50 μm. Dorsal is up in all images. The individual numerical values for panels A and B can be found in S1 Data. The genotypes can be found in S1 Text. APF, after puparium formation; cul1, cullin1; mical, molecule interacting with CasL; n.s., not significant; prd1, pruning defect 1. (TIF) [file pbio.2004506.s023.tif]

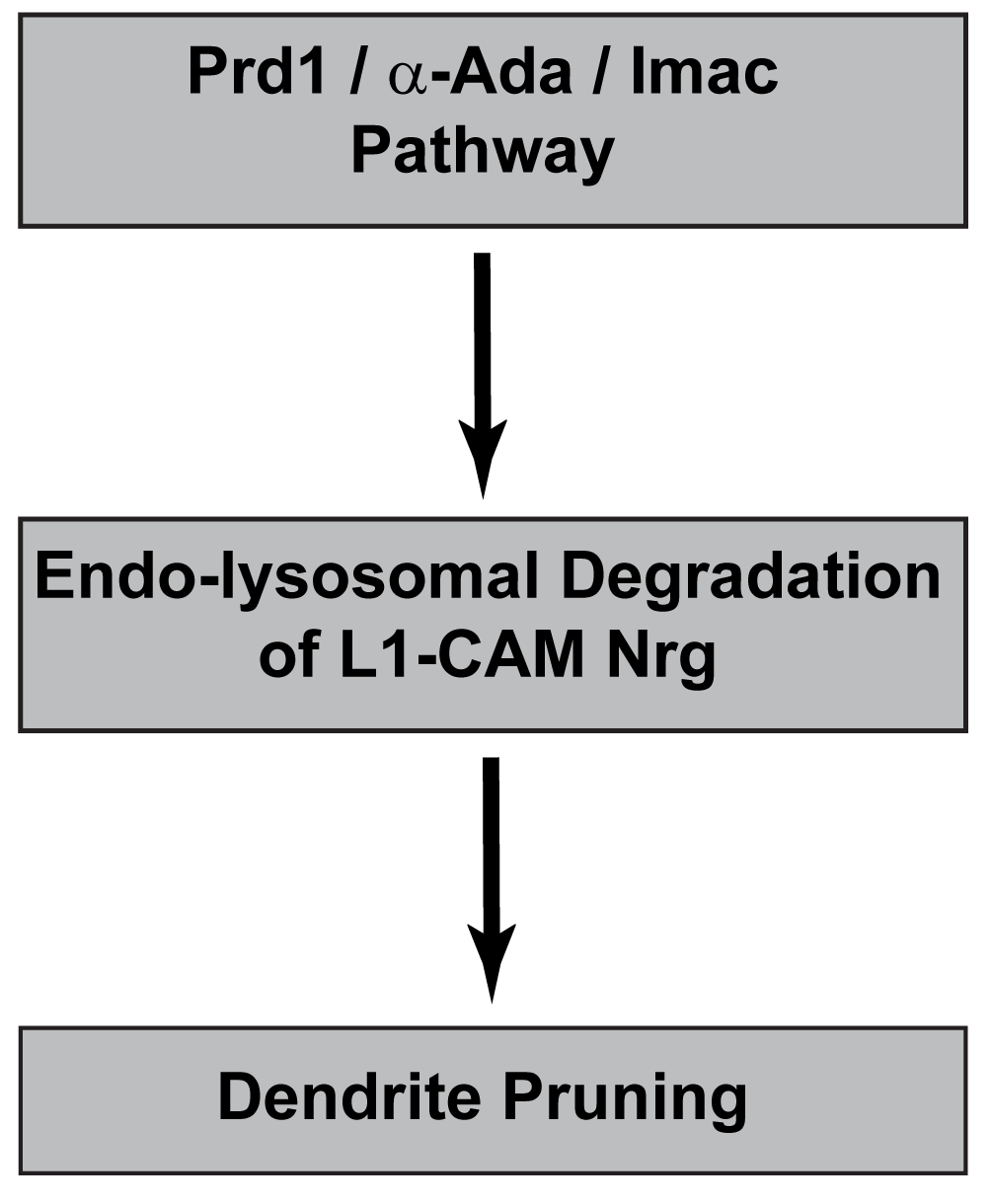

Supplement: S24 Fig — We propose that Prd1, α-Ada, and Imac act in the same pathway to promote dendrite pruning via endo-lysosomal degradation of the L1-CAM Nrg. Down-regulation of Nrg is required to trigger dendrite pruning in ddaC neurons during early metamorphosis. α-Ada, α-Adaptin; Imac, Immaculate connections; L1-CAM, L1-type cell adhesion molecule; Nrg, Neuroglian; Prd1, Pruning defect 1. (TIF) [file pbio.2004506.s024.tif]

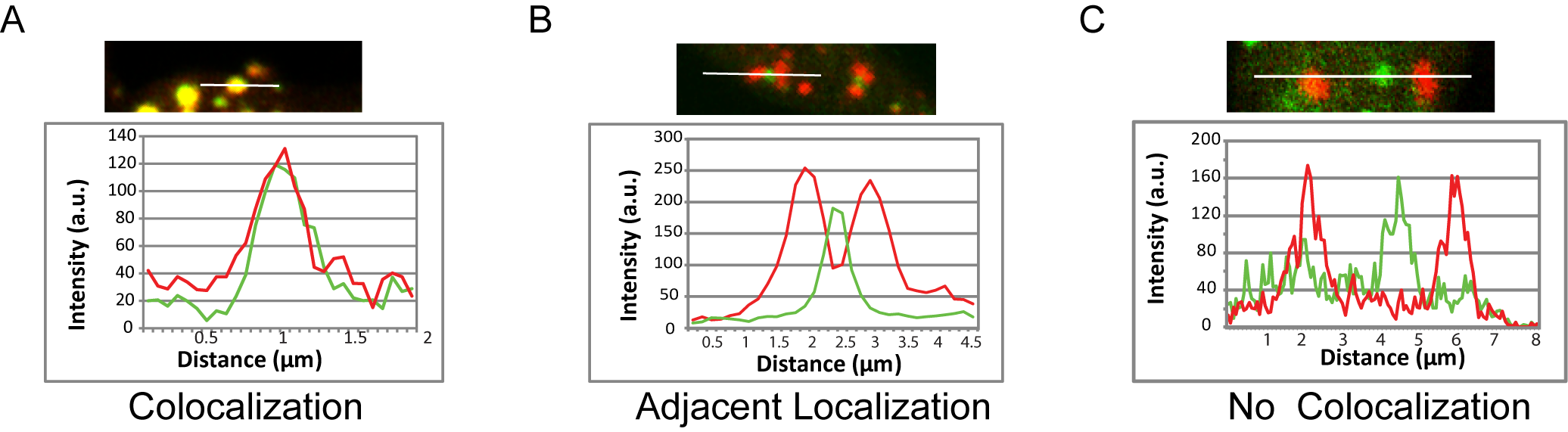

Supplement: S25 Fig — Based on the intensity profiles, the localization patterns were divided into three categories: (A) colocalization, (B) adjacent localization, (C) non-colocalization. The genotypes can be found in S1 Text. (TIF) [file pbio.2004506.s025.tif]
