# Supplementary material for: Prd1 associates with the clathrin adaptor α-Adaptin and the kinesin-3 Imac/Unc-104 to govern dendrite pruning in Drosophila
Source: PLoS Biol. 2018 Aug 24;16(8):e2004506. doi: 10.1371/journal.pbio.2004506 (PMC6126864; doi:10.1371/journal.pbio.2004506)
Supplement: S1 Text — (DOCX) [file pbio.2004506.s026.docx]

**Fig 1:** (B) *w*; ppk-Gal4, UAS-mCD8GFP* / *UAS-control RNAi; UAS-Dcr2* / *+.* (C) *w*; ppk-Gal4, UAS-mCD8GFP* / *UAS-prd1* RNAi #1*; UAS-Dcr2* / *+*. (D) *w*; ppk-Gal4, UAS-mCD8GFP* / +; *prd1^M56^* / *Df(3R)Exel7310*. (E) *w*; ppk-Gal4, UAS-mCD8GFP* / *UAS-Prd1*; *prd1^M56^*/ *Df(3R)Exel7310*. (F) *w*; ppk-Gal4, UAS-mCD8GFP* / *UAS-Venus-Prd1*; *prd1^M56^*/ *Df(3R)Exel7310*. (G) *Gal4^5-40^, UAS-Venus:pm, SOP-flp #42, w** / *elav-Gal4, UAS-mCD8GFP, hs-FLP, w*;; FRT82B, prd1^PS1^* / *FRT82B, tubP-Gal80.* (H) *Gal4^5-40^, UAS-Venus:pm, SOP-flp #42, w** / *elav-Gal4, UAS-mCD8GFP, hs-FLP, w*; UAS-Prd1/+; FRT82B, prd1^PS1^* / *FRT82B, tubP-Gal80.*

**Fig 2:** (A) *w**; *ppk-Gal4* / *UAS-GFP-Rab5; UAS-Venus-Prd1* / *+.* (B) *w**; *ppk-Gal4* / *+; UAS-Venus-Prd1* / *UAS-GFP-α-Ada.* (C) *w**; *ppk-Gal4* / *UAS-GFP-Clc; UAS-Venus-Prd1*/*+.* (D) *w**; *ppk-Gal4* / *UAS-mRFP-Chc; UAS-Venus-Prd1* / *+.* (E) *w**; *ppk-Gal4* / +*; UAS-Venus-Prd1* / *UAS-PLC-δ-PH-GFP.* (F) **Control RNAi:** *w*; ppk-Gal4, UAS-Dcr2* / *+; UAS-GFP-α-Ada* / *UAS-control RNAi.* ***prd1* RNAi #1:** *w*; ppk-Gal4,* *prd1* RNAi #1 / +*; UAS-GFP-α-Ada* / *UAS-Dcr2.* (G) **Control RNAi:** *w*; ppk-Gal4, UAS-Dcr2* / *+; UAS-Venus-Prd1* / *UAS-control RNAi.* ***α-ada* RNAi #1:** *w*; ppk-Gal4,UAS-Dcr2* / *+;UAS-Venus-Prd1* /*UAS-α-ada RNAi #1.*

**Fig 4:** (A) *w*; FRT40A* / *tub-Gal80, FRT40A; ppk-Gal4, UAS-mCD8GF, SOP-flp* / *+.* (B) *w*; α-ada^3^, FRT40A* / *tubP-Gal80, FRT40A; ppk-Gal4, UAS-mCD8­GFP, SOP-flp* / *+.* (C) *w*; α-ada^3^, FRT40A* / *tubP-Gal80, FRT40A; ppk-Gal4, UAS-mCD8­GFP, SOP-flp* / *UAS-GFP-α-Ada.* (D) *w*, Bap^Δ1^, FRT19A* / *w*, tub-Gal80, hs-FLP, FRT19A; ppk-Gal4, UAS-mCD8GFP, SOP-flp* / *+.* (E) *w*, Bap^Δ1^, FRT19A* / *w*, tub-Gal80, hs-FLP, FRT19A; ppk-Gal4, UAS-mCD8GF, SOP-flp* / *UAS-Bap.* (F) *w*; ppk-Gal4, UAS-mCD8­GFP, SOP-flp* / *+; FRT82B, AP-2µ^NN20^* / *FRT82B, tubP-Gal80.*

**Fig 5:** (A) *w**; *ppk-Gal4* / +*; UAS-Venus-Prd1* /*UAS-Imac-RFP.* (B) *w**; *ppk-Gal4* / *+; UAS-Imac-RFP* / *UAS-GFP-α-Ada.* (C) *w**; *ppk-Gal4* / *+; UAS-Imac-RFP, UAS-GFP-α-Ada* /*UAS-Venus-Prd1.* (D) *w**; *ppk-Gal4* / *UAS-GFP-Rab5; UAS-Imac-RFP* /*UAS-Venus-Prd1.*

**Fig 6:** (A) **Control:** *w*; ppk-Gal4, UAS-mCD8GFP* / *+; UAS-Dcr2* / *UAS-control RNAi.* (B) *w*; ppk-Gal4, UAS-mCD8GFP* / *+; UAS-Dcr2* / *UAS-imac RNAi #1.* (C) *w** / *elav-Gal4, UAS-mCD8GFP, hs-FLP, w*; FRT 42D, imac^170^* / *FRT 42D, tubP-Gal80; ppk-Gal4, UAS-mCD8­GFP, SOP-flp* / *+.* (D) *w** / *elav-Gal4, UAS-mCD8GFP, hs-FLP, w*; FRT 42D, imac^170^* / *FRT42D, tubP-Gal80; ppk-Gal4, UAS-mCD8­GFP, SOP-flp* / *UAS-Imac-RFP.* (E) **O/E *Imac^G102E^* (Non-induced):** *w*;* GSG2295-*Gal4, ppk-tdGFP/ +;* *+ / UAS-Imac^G102E^.* (F) **O/E *Imac^G102E^* (Induced):** *w*;* GSG2295-*Gal4, ppk-tdGFP/ +;* *+ / UAS-Imac^G102E^.*

**Fig 7:** (A) **Control:** *w*; FRT 42D* / *FRT 42D, tubP-Gal80; ppk-Gal4, UAS-mCD8­GFP, SOP-flp* / *+.* ***imac* RNAi:** *w*; ppk-Gal4, UAS-mCD8GFP* / *+; UAS-Dcr2* / *UAS-imac RNAi #1.* ***Imac^170^* MARCM:** *w** / *elav-Gal4, UAS-mCD8GFP, hs-FLP, w*; FRT 42D, imac^170^* / *FRT 42D, tubP-Gal80; ppk-Gal4, UAS-mCD8­GFP, SOP-flp* / *+.* (B) **Control RNAi:** *w**; *ppk-Gal4, UAS-Dcr2* / +*; UAS-Venus-Prd1* /*UAS-control RNAi.* ***imac* RNAi #1:** *w**; *ppk-Gal4, UAS-Dcr2* / +*; UAS-Venus-Prd1* /*UAS-imac RNAi #1.* (C) **Control:** *w*; FRT 42D* / *FRT 42D, tubP-Gal80; ppk-Gal4, UAS-mCD8­GFP, SOP-flp* / *+.* ***imac* RNAi:** *w*; ppk-Gal4,UAS-Dicer2+; UAS-Venus-Prd1* / *UAS-imac RNAi #1.* ***imac^170^* MARCM:** *w** / *elav-Gal4, UAS-mCD8GFP, hs-FLP, w*; FRT 42D, imac^170^* / *FRT 42D, tubP-Gal80; ppk-Gal4, UAS-mCD8­GFP, SOP-flp* / *+.* (D) **Control RNAi:** *w**; *ppk-Gal4, UAS-Dcr2* / UAS*-mRFP-Chc; +* /*UAS-control RNAi.* ***imac* RNAi:** *w**; *ppk-Gal4, UAS-Dcr2* / UAS*-mRFP-Chc; +* /*UAS-imac RNAi #1.* (E) **Control RNAi:** *w**; *ppk-Gal4, UAS-Dcr2* / +*; UAS-Venus-Prd1* / *UAS-control RNAi.* ***imac* RNAi:** *w**; *ppk-Gal4, UAS-Dcr2* / +*; UAS-Venus-Prd1* /*UAS-imac RNAi #1.* (F) **Control RNAi:** *w**; *ppk-Gal4, UAS-Dcr2* / +*; UAS-Venus-Prd1* / *UAS-control RNAi.* ***imac* RNAi:** *w**; *ppk-Gal4, UAS-Dcr2* / +*; UAS-Venus-Prd1* /*UAS-imac RNAi #1.*

**Fig 8:** (A) ***imac^170^/ +*:** *w*; FRT 42D, imac^170^* / *ppk-Gal4, UAS-mCD8­GFP*. ***prd1^M56^/ Df*:** *w*; ppk-Gal4, UAS-mCD8GFP* /+; *prd1^M56^*/ *Df(3R)Exel7310*. ***imac^170^/ +; prd1^M56^/ Df*:** *w*; FRT 42D, imac^170^* / *ppk-Gal4, UAS-mCD8­GFP; prd1^M56^*/ *Df(3R)Exel7310.* (B) ***imac^172^ /+*:** *w*; FRT 42D, imac^172^* / *ppk-Gal4, UAS-mCD8­GFP*. ***prd1^M56^/ Df*:** *w*; ppk-Gal4, UAS-mCD8GFP* /+; *prd1^M56^*/ *Df(3R)Exel7310*. ***imac^172^/ +; prd1^M56^/ Df*:** *w*; FRT 42D, imac^172^* / *ppk-Gal4, UAS-mCD8­GFP; prd1^M56^*/ *Df(3R)Exel7310*. (C) ***α-ada^3^/ +*:** *w*; α-ada^3^, FRT 40A* / *ppk-Gal4, UAS-mCD8­GFP*. ***prd1^M56^/ Df*:** *w*; ppk-Gal4, UAS-mCD8GFP* /+; *prd1^M56^*/ *Df(3R)Exel7310*. ***α-ada^3^/+; prd1^M56^/ Df*** **:** *w*; α-ada^3^, FRT 40A* / *ppk-Gal4, UAS-mCD8­GFP; prd1^M56^*/ *Df(3R)Exel7310*. (D)  ***prd1^M56^/ +*:** *w*; ppk-Gal4, UAS-mCD8­GFP/+;* *prd1^M56^/+*. ***α-ada* RNAi #2/+:** *w*; ppk-Gal4, UAS-mCD8GFP* /+; *UAS-α-ada RNAi #2/+*. ***prd1^M56^*/ *α-ada* RNAi #2:** *w*; ppk-Gal4, UAS-mCD8GFP* /+; *UAS-α-ada RNAi #2/ prd1^M56^*. (E) ***α-ada^3^/ +*:** *w*; α-ada^3^, FRT 40A* / *ppk-Gal4, UAS-mCD8­GFP, UAS-Dcr2*. ***imac* RNAi / +:** *w*; ppk-Gal4, UAS-mCD8GFP*,*UAS-Dcr2* /+; *UAS-imac RNAi #1/+*. ***α-ada^3^ /+; imac* RNAi / +:** *w*; α-ada^3^, FRT 40A / ppk-Gal4, UAS-mCD8GFP*,*UAS-Dcr2*; *UAS-imac RNAi #1/ +*.

**S1 Fig:** (C) **FRT82B control:** *w*; ppk-Gal4, UAS-mCD8­GFP, SOP-flp* / *+; FRT82B* / *FRT82B, tubP-Gal80.* ***prd1^M56^* MARCM:** *w*; ppk-Gal4, UAS-mCD8­GFP, SOP-flp* / *+; FRT82B, prd1^M56^* / *FRT82B, tubP-Gal80.* (D) **Control**: *w*; ppk-Gal4, UAS-mCD8GFP* / *UAS-control RNAi; UAS-Dcr2* / *+.* ***prd1^PS1^* MARCM:***Gal4^5-40^, UAS-Venus:pm, SOP-flp #42, w** / *elav-Gal4, UAS-mCD8GFP, hs-FLP, w*;; FRT82B, prd1^PS1^* / *FRT82B, tubP-Gal80.*

**S2 Fig:** (A) **Control:** *w*;; Gal4 ^2-21^, UAS-mCD8GFP* / *Gal4^2-21^, UAS-mCD8GFP.* ***prd1^M56^* MARCM:** *Gal4^5-40^, UAS-Venus:pm, SOP-flp #42, w** / *elav-Gal4, UAS-mCD8GFP, hs-FLP, w*;; FRT82B, prd1^M56^* / *FRT82B, tubP-Gal80.* (B) **FRT82B control:** *w*; ppk-Gal4, UAS-mCD8­GFP, SOP-flp* / *+; FRT82B* / *FRT82B, tubP-Gal80.* ***prd1^M56^* MARCM:** *w*; ppk-Gal4, UAS-mCD8­GFP, SOP-flp* / *+; FRT82B, prd1^M56^* / *FRT82B, tubP-Gal80.*

**S3 Fig:** (B) *w**; *ppk-Gal4* / *+; UAS-Venus-Prd1* / +. (C) *w**; *ppk-Gal4* / *+; UAS-Venus-Prd1* / *UAS-Arf79F-EGFP.*

**S4 Fig:** (A) **Control:** *w**; *ppk-Gal4* / +*; UAS-Venus-Prd1* / *+.* ***Rab5^DN^*:** *w**; *ppk-Gal4, UAS-Rab5^DN^* / +*; UAS-Venus-Prd1* / *+.* **Control:** *w**; *ppk-Gal4* / +*; UAS-GFP-α-Ada* / *+.* ***Rab5^DN^*:** *w**; *ppk-Gal4, UAS-Rab5^DN^* / *+; UAS-GFP-α-Ada* / *+.* **Control:** *w**; *ppk-Gal4* / *UAS-GFP-Clc; +* / *+.* ***Rab5^DN^*:** *w**; *ppk-Gal4, UAS-Rab5^DN^* / *UAS-GFP-Clc; +* / *+.* (B) **Control:** *w**; *ppk-Gal4* / +*; UAS-Venus-Prd1* / *+.* ***VPS4^DN^*:** *w**; *ppk-Gal4, UAS-VPS4^DN^* / +*; UAS-Venus-Prd1* / *+.* **Control:** *w**; *ppk-Gal4* / *+; UAS-GFP-α-Ada* / *+.* ***VPS4^DN^*:** *w**; *ppk-Gal4, UAS-VPS4^DN^* / +*; UAS-GFP-α-Ada* / *+.* **Control:** *w**; *ppk-Gal4* / *UAS-GFP-Clc; +* / *+.* ***VPS4^DN^*:** *w**; *ppk-Gal4, UAS-VPS4^DN^* / *UAS-GFP-Clc; +* / *+.*

**S5 Fig:** **Control** (Mito-GFP): *w*; ppk-Gal4* / *+; UAS-Mito-GFP* / *+.* ***Rab5^DN^*:** *w*;* *ppk-Gal4, UAS-Rab5^DN^*/ *+; UAS-Mito-GFP* / *+.* ***VPS4^DN^*:** *w*; ppk-Gal4, UAS-Vps4^DN^*/ *+; UAS-Mito-GFP* / *+.* **Control** (GM130 and KDEL): *w*; ppk-Gal4* / *+; ppk-Gal4, UAS-mCD8GFP* / *+.* ***Rab5^DN^***: *w*; ppk-Gal4, UAS-Rab5^DN^* / *+; ppk-Gal4, UAS-mCD8GFP* /*+.* ***Vps4^DN^***: *w*; ppk-Gal4, UAS-Vps4^DN^* / *+; ppk-Gal4, UAS-mCD8GFP* / *+.*

**S8 Fig: Control RNAi:** *w*; ppk-Gal4, UAS-mCD8GFP* / *+; UAS-Dcr2* / *UAS-control RNAi.* ***α-ada* RNAi #1:** *w*; ppk-Gal4, UAS-mCD8GFP* / *+; UAS-Dcr2* / *UAS-α-ada RNAi #1.* ***α-ada* RNAi #2:** *w*; ppk-Gal4* / *+; ppk-Gal4, UAS-mCD8GFP* / *UAS-α-ada RNAi #2.*

**S9 Fig:** (A) **Control:** *w*;; Gal4 ^2-21^, UAS-mCD8GFP* / *Gal4^2-21^, UAS-mCD8GFP.* ***α-ada^3^* MARCM:** *Gal4^5-40^, UAS-Venus:pm, SOP-flp #42, w** / *elav-Gal4, UAS-mCD8GFP, hs-FLP, w*; α-ada^3^, FRT40A* / *tubP-Gal80, FRT40A*. (B) **Control:** *w*; ppk-Gal4, UAS-mCD8­GFP, SOP-flp* / *+; FRT40A* / *tubP-Gal80, FRT40A.* ***α-ada^3^* MARCM:** *w*; ppk-Gal4, UAS-mCD8­GFP, SOP-flp* / *+; α-ada^3^, FRT40A* / *tubP-Gal80, FRT40A.* (D) **Wild type:** *w*;; ppk-Gal4, UAS-mCD8GFP / ppk-Gal4, UAS-mCD8GFP.*

**S10 Fig: Control:** *w*;; ppk-Gal4, UAS-mCD8GFP / ppk-Gal4, UAS-mCD8GFP.*  ***AP-1μ^SHE-11^***: *w*; ppk-Gal4, UAS-mCD8­GFP, SOP-flp* / *+; FRT82B, AP-1μ^SHE-11^* / *FRT82B, tubP-Gal80.* ***AP-1γ^B^*:** *AP-1γ^B^, FRT19A / tub-Gal80, hs-FLP, FRT19A, w*; ppk-Gal4, UAS-mCD8GFP, SOP-flp / +.* ***AP-1γ^D^*:** *AP-1γ^D^, FRT19A / tub-Gal80, hs-FLP, FRT19A, w*; ppk-Gal4, UAS-mCD8GFP, SOP-flp / +.*

**S11 Fig:** (A) *w**; *ppk-Gal4* / +*; UAS-Venus-Prd1* /*UAS-Khc-GFP.* (B) *w**; *ppk-Gal4* / +*; UAS-Venus-Prd1* /*UAS-Kap3-RFP*. (C) *w**; *ppk-Gal4* / +*; UAS-Venus-Prd1* / *UAS-Dlic-EGFP.* (D) *w**; *ppk-Gal4* / +*; UAS-Venus-Prd1* / *UAS-mRFP-Lis1.*

**S12 Fig:** (A) *w**; *ppk-Gal4* / *+; UAS-Venus-Prd1* / *UAS-GFP-α-Ada.* (B) *w**; *ppk-Gal4* / +*; UAS-Venus-Prd1* /*UAS-Imac-RFP.* (C) *w**; *ppk-Gal4* / *+; UAS-Imac-RFP* / *UAS-GFP-α-Ada.* (D) *w**; *ppk-Gal4* / *+; UAS-Imac-RFP, UAS-GFP-α-Ada* /*UAS-Venus-Prd1.*

**S14 Fig: *imac* RNAi #2:** *w*; ppk-Gal4, UAS-mCD8GFP* / *+; UAS-Dcr2* / *UAS-imac RNAi #2.* **O/E *Imac^G102E^*:** *w*; ppk-Gal4* / *+; ppk-Gal4, UAS-mCD8GFP* / *UAS-Imac^G102E^.* **O/E *Imac^AAA^*:** *w*; ppk-Gal4* / *+; ppk-Gal4, UAS-mCD8GFP* / *UAS-Imac^AAA^.*

**S15 Fig:** **O/E Control (Induced):** *w*;* GSG2295-*Gal4, ppk-tdGFP / +;* *UAS-Mical^NT^/+****.* O/E *Imac^AAA^* (Induced):** *w*;* GSG2295-*Gal4, ppk-tdGFP / +;* *+/UAS-Imac^AAA^****.***

**S16 Fig:** **Control RNAi (Venus-Prd1):** *w**; *ppk-Gal4, UAS-Dcr2* / +*; UAS-Venus-Prd1* /*UAS-control RNAi.* ***imac* RNAi (Venus-Prd1):** *w**; *ppk-Gal4, UAS-Dcr2* / +*; UAS-Venus-Prd1* /*UAS-imac RNAi #2.* **Control RNAi (GFP-α-Ada):** *w**; *ppk-Gal4, UAS-Dcr2* / +*; UAS-GFP-α-Ada* /*UAS-control RNAi.* ***imac* RNAi (GFP-α-Ada):** *w**; *ppk-Gal4, UAS-Dcr2* / +*; UAS-GFP-α-Ada* /*UAS-imac RNAi #2.*

**S17 Fig:** **Control (Venus-Prd1)**: *w*; ppk-Gal4, UAS-mCD8GFP, UAS-Dcr2* / *+; UAS-control RNAi* / *UAS-Venus-Prd1.* ***khc* RNAi (Venus-Prd1):** *w*; ppk-Gal4, UAS-mCD8GFP, UAS-Dcr2* / *+; UAS-khc RNAi* / *UAS-Venus-Prd1.* **Control (α-Ada):** *w*; ppk-Gal4, UAS-mCD8GFP* / *ppk-Gal4, UAS-mCD8GFP; UAS-Dcr2* / *UAS-Dcr2.* ***khc* RNAi (α-Ada):** *w*; ppk-Gal4, UAS-mCD8GFP, UAS-Dcr2* / *ppk-Gal4, UAS-mCD8GFP, UAS-Dcr2; UAS-khc RNAi* / *UAS-khc RNAi.*

**S18 Fig:** (A) **Control RNAi:** *w**; *ppk-Gal4, UAS-Dcr2* / +*; UAS-Venus-Prd1* /*UAS-control RNAi.* ***imac* RNAi:** *w**; *ppk-Gal4, UAS-Dcr2* / +*; UAS-Venus-Prd1* /*UAS-imac RNAi #1.* (B) **Control RNAi:** *w**; *ppk-Gal4, UAS-Dcr2* / *UAS-GFP-Rab5; UAS-Venus-Prd1* / *UAS-control RNAi.* ***imac* RNAi:** *w**; *ppk-Gal4, UAS-Dcr2* / *UAS-Rab5-GFP; UAS-Venus-Prd1* /*UAS-imac RNAi #1.* (C) **Control:** *w**; *ppk-Gal4, UAS-Dcr2* / +; *UAS-Venus-Prd1/UAS-control RNAi.* ***imac* RNAi:** *w**; *ppk-Gal4, UAS-Dcr2* / *+; UAS-Venus-Prd1* /*UAS-imac RNAi #1.* (E) **Control:** *w**; *ppk-Gal4, UAS-Dcr2* / +; *UAS-GalT-GFP/ UAS-control RNAi.* ***imac* RNAi:** *w**; *ppk-Gal4, UAS-Dcr2* / *+; UAS-GalT-GFP* /*UAS-imac RNAi #1.*

**S19 Fig:** (A) **Control:** *w*;; ppk-Gal4, UAS-mCD8GFP / ppk-Gal4, UAS-mCD8GFP* (B) *w*; ppk-Gal4, UAS-mCD8GFP* / +; *prd1^M56^* / *Df(3R)Exel7310*. (C) *w*; α-ada^3^, FRT40A* / *tubP-Gal80, FRT40A; ppk-Gal4, UAS-mCD8­GFP, SOP-flp* / *+.* (D) **O/E *Imac^G102E^* (Induced):** *w*;* GSG2295-*Gal4, ppk-tdGFP / +;* *+/UAS-Imac^G102E^****.***

**S20 Fig**: (A) ***prd1^M56^ / prd1^PS2^*:** *w*; ppk-Gal4, UAS-mCD8GFP* / +; *prd1^M56^* / *prd1^PS2^*. ***prd1^M56^ / prd1^PS2^*;1x *nrg-i*:** *w*; ppk-Gal4, UAS-mCD8GFP* / *UAS-nrg* RNAi; *prd1^M56^* / *prd1^PS2^*. (B) ***α-ada^3^* MARCM:** *w*; α-ada^3^, FRT40A* / *tubP-Gal80, FRT40A; ppk-Gal4, UAS-mCD8­GFP, SOP-flp* / *+.* ***ada^3^* MARCM; 1 x *nrg-i*:** *w*; α-ada^3^, FRT40A, UAS-nrg* RNAi / *tubP-Gal80, FRT40A; ppk-Gal4, UAS-mCD8­GFP, SOP-flp* / *+.* (C) ***imac* RNAi; 1x control RNAi:** *w*; ppk-Gal4, UAS-mCD8GFP* / *UAS-control RNAi; UAS-Dcr2* / *UAS-imac RNAi #1.* ***imac* RNAi; 1x *nrg* RNAi:** *w*; ppk-Gal4, UAS-mCD8GFP* / *UAS-nrg RNAi; UAS-Dcr2* / *UAS-imac RNAi #1.*

**S21 Fig: Control:** *w**; *ppk-Gal4, UAS-GCaMP3* / +*; UAS-Mical^N-ter^*. ***prd1^M56^/ Df*:** *w*; ppk-Gal4, UAS-GCaMP3* / *+;* *prd1^M56^*/ *Df(3R)Exel7310*. ***O/E Imac^G102E^*:** *w*;* GSG2295-*Gal4 / +;* *UAS-GCaMP6 / UAS-Imac^G102E^.*

**S23 Fig:** (A) ***mical^15256^*/+:** *w*; ppk-Gal4, UAS-mCD8­GFP/+;FRT82B,mical^15256^/+*. ***prd1^M56^/Df*:** *w*; ppk-Gal4, UAS-mCD8GFP* /+; *prd1^M56^*/ *Df(3R)Exel7310*. ***mical^15256^, prd1^M56^/Df*:** *w*; ppk-Gal4, UAS-mCD8­GFP/+; FRT82B,* *mical^15256^, prd1^M56^*/ *Df(3R)Exel7310*. (B) ***cul1^Ex^*/+:** *w*; FRT G13,* *cul1^Ex^/ ppk-Gal4, UAS-mCD8­GFP;+/+*. ***prd1^M56^/Df*:** *w*; ppk-Gal4, UAS-mCD8GFP* /+; *prd1^M56^*/ *Df(3R)Exel7310*. ***cul1^Ex^*/+; *prd1^M56^/Df*:** *w*; FRT G13,* *cul1^Ex^/ ppk-Gal4, UAS-mCD8­GFP; prd1^M56^*/ *Df(3R)Exel7310*.

**S25 Fig:** (A) *w**; *ppk-Gal4* / +*; UAS-Venus-Prd1* /*UAS-Imac-RFP.* (B) *w**; *ppk-Gal4* / *UAS-GFP-Rab5; UAS-Venus-Prd1* / *+.* (C) *w**; *ppk-Gal4* / *+; UAS-Imac-RFP* / *UAS-GFP-α-Ada.*
